# Versatile rare earth hexanuclear clusters for the design and synthesis of highly-connected **ftw**-MOFs†
†Electronic supplementary information (ESI) available: Materials and methods, synthesis of ligands, NMR spectra, synthesis of MOFs, additional structural figures, PXRD, TGA, low and high pressure gas adsorption isotherms, *Q*_st_ analysis and IAST calculation. CCDC 1050062–1050064. For ESI and crystallographic data in CIF or other electronic format see DOI: 10.1039/c5sc00614g


**DOI:** 10.1039/c5sc00614g

**Published:** 2015-04-15

**Authors:** Ryan Luebke, Youssef Belmabkhout, Łukasz J. Weseliński, Amy J. Cairns, Mohamed Alkordi, George Norton, Łukasz Wojtas, Karim Adil, Mohamed Eddaoudi

**Affiliations:** a Functional Materials Design, Discovery & Development Research Group (FMD3) , Advanced Membranes & Porous Materials Center , Division of Physical Sciences and Engineering , King Abdullah University of Science and Technology (KAUST) , Thuwal 23955-6900 , Kingdom of Saudi Arabia . Email: mohamed.eddaoudi@kaust.edu.sa; b Department of Chemistry , University of South Florida , 4202 East Fowler Avenue , Tampa , Florida 33620 , USA

## Abstract

A series of highly porous MOFs were deliberately targeted to contain a 12-connected rare earth hexanuclear cluster and quadrangular tetracarboxylate ligands. The resultant MOFs have an underlying topology of **ftw**, and are thus (4,12)-c **ftw**-MOFs. This targeted rare earth **ftw**-MOF platform offers the potential to assess the effect of pore functionality and size, *via* ligand functionalization and/or expansion, on the adsorption properties of relevant gases. Examination of the gas adsorption properties of these compounds showed that the **ftw**-MOF-2 analogues, constructed from rigid ligands with a phenyl, naphthyl, or anthracene core exhibited a relatively high degree of porosity. The specific surface areas and pore volumes of these analogs are amongst the highest reported for RE-based MOFs. Further studies revealed that the Y-**ftw**-MOF-2 shows promise as a storage medium for methane (CH_4_) at high pressures. Furthermore, Y-**ftw**-MOF-2 shows potential as a separation agent for the selective removal of normal butane (*n*-C_4_H_10_) and propane (C_3_H_8_) from natural gas (NG) as well as interesting properties for the selective separation of *n*-C_4_H_10_ from C_3_H_8_ or isobutane (iso-C_4_H_10_).

## Abstract 2

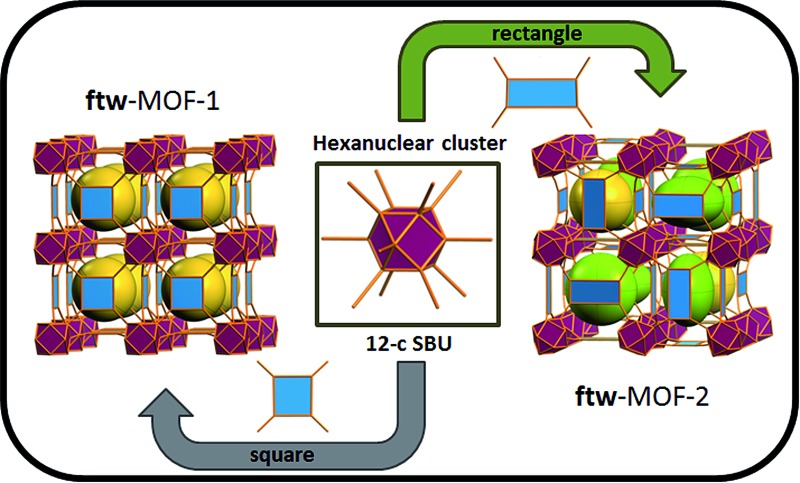
A targeted rare earth **ftw**-MOF platform offers the potential to assess the effect of pore functionality and size on gas adsorption *via* ligand functionalization and/or expansion.

## Introduction

**Fig. 1:**
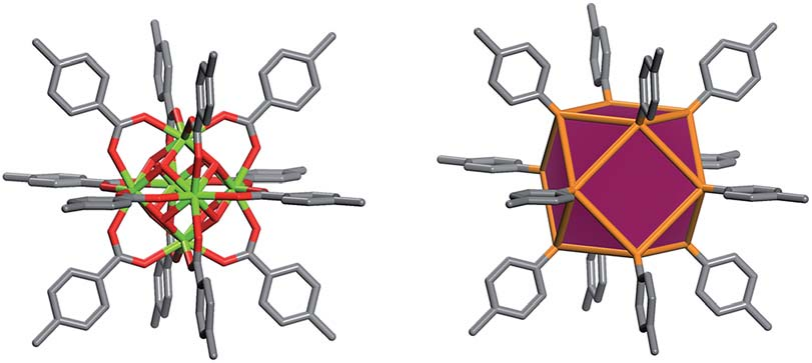
Left, the 12-connected rare earth hexanuclear cluster, [RE_6_(μ_3_-OH)_8_(O_2_C–)_12_].^11^ Right, illustration of the MBB coordination geometry: note the positions of the carbon atom of the carboxylates match the vertices of the cuboctahedron.

Metal Organic Frameworks (MOFs), a unique and emerging class of highly tunable porous materials, have attracted wide interest in recent years due to their unprecedented tunability and high degree of porosity.^1^ The ability to introduce desired functionality during MOF assembly^2^ or post-synthetically^3^ contributes to their great potential in addressing technological challenges in areas of gas storage,^4^,^5^ gas separation,^6^,^7^ catalysis,^8^ and chemical sensing.^9^ Various design strategies offer pathways to synthesize materials for specific applications through exploitation of the modular nature of MOF materials. The molecular building block (MBB) approach offers the potential to design MOFs where structural information is included into the building blocks (*i.e.* the organic ligands and inorganic clusters). Successful implementation of reticular chemistry and the MBB approach requires isolation of the synthetic conditions that promote the formation of a desired inorganic MBB to minimize the number of possible resulting framework topologies. Our group, among others, seeks to leverage the knowledge that MOFs – composed of highly-connected building blocks which limits the number of possible topological outcomes and thus leads to a greater degree of predictability in structure^10^ – are suitable platforms to purposely tune the resultant materials properties. Recently researchers have identified reaction conditions conducive to the formation of a hexanuclear Zr/Hf based cluster. This has resulted in a diversity of examples of robust functional MOFs.^12^–^14^ Our recent work has elucidated the reaction conditions (*i.e.* incorporation of fluorine containing ligands or using 2-fluorobenzoic acid as a reaction modulator) necessary to promote the formation of a similar highly connected (12-connected) rare earth based hexanuclear MBB (Fig. 1).^15^,^16^ Uniquely, this RE cluster presents advantages over the Zr/Hf cluster as it offers potential to tune the resulting material properties^15^ through use of different rare earth metals. It was shown that linking these 12-connected RE-MBBs through linear ligands results in the formation of MOFs with **fcu** topology.^15^ Specifically, in the hexanuclear [RE_6_(OH)_8_(O_2_C–)_12_] carboxylate-based cluster the carboxylate carbon atoms of the coordinated ligands act as points of extension and correspond to the vertices of the **fcu**-a net (vertex figure of the **fcu** net with a cuboctahedron geometry (Fig. 1)).

**Fig. 2:**
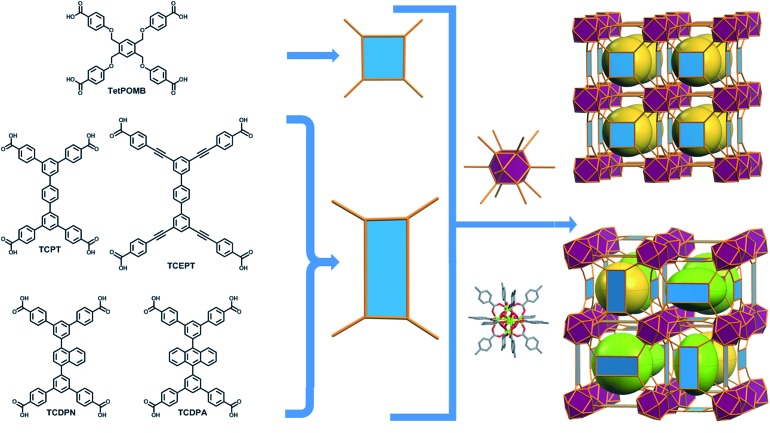
Schematic showing the assembly of **ftw**-MOFs by combining 4-connected ligands (TetPOMB, TCPT, TCEPT, TCDPN, and TCDPA) with 12-connected rare earth hexanuclear MBBs (with a square or rectangle combined with a cuboctahedron vertex figure respectively), resulting in the **ftw** topology shown here as the augmented **ftw** net.

**Fig. 3:**
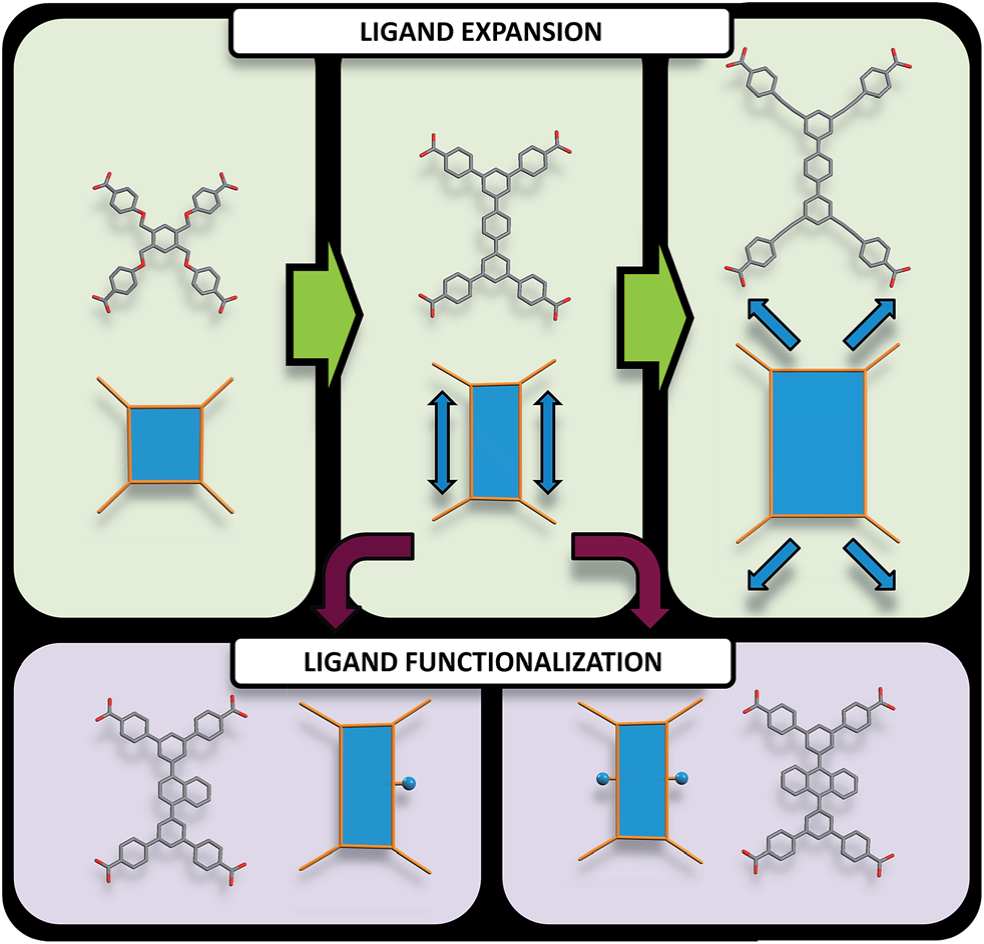
Pathways to modify quadrangular ligands for the synthesis of **ftw**-MOFs, including examples of ligands and their vertex figures. (Top) Modification of the organic quadrangular MBB through ligand expansion. (Bottom) Modification of the quadrangular organic MBB through ligand functionalization.

The aforementioned RE-based hexanuclear cluster is a suitable MBB for the rational design of highly-connected MOFs as its inherent geometry and high connectivity permit only a limited number of possible topological outcomes for subsequent assembly, making it ideal for the effective practice of reticular chemistry. In this work, the focus is on utilizing 4-connected quadrangular carboxylate-based ligands (Fig. 2 and 3) to synthesize rare earth MOFs having both 12-c and 4-c nodes. There are a total of three known (4,12)-c edge transitive binodal nets, all of which are constructed from one kind of edge and therefore offer the prospect of constructing MOFs with such topologies through use of a single type of symmetrical ligand. Of the three (4,12)-c nets (**shp**, **ftw**, **ith**),^10^ the **ftw** topology is the only one composed of 12-connected nodes matching the cuboctahedron vertex figure in the 12-c RE hexanuclear MBB, [RE_6_(OH)_8_(O_2_C–)_12_], (Fig. 1).

Accordingly RE-MOFs with an underlying topology of **ftw** (**ftw**-MOFs) have been purposely targeted^13^,^14^,^17^–^19^ by employing 4-connected quadrangular tetracarboxylate ligands in combination with the 12-c cuboctahedron MBB. This targeted RE **ftw**-MOF platform offers the potential to systematically assess the effect of pore functionality and size *via* ligand functionalization and/or expansion (Fig. 3) by investigating the adsorption properties of relevant gases.

Herein we report a successful reticular chemistry method, where the newly isolated 12-c RE MBB allows the synthesis of a series of highly porous RE-MOFs including Y-**ftw**-MOF-2, which to the best of our knowledge has the highest specific surface area ever reported for a RE-MOF. Specifically, a tetratopic carboxylate-based ligand with a square-like geometry was reacted with a variety of rare earth metal salts under appropriate reaction conditions – including the use of a fluorinated modulator^15^ – to predictably form **ftw**-MOFs (**ftw**-MOF-1). Furthermore, ligand expansion was achieved *via* elongating the central core and/or extending the four arms, resulting in two relatively more open **ftw**-derived MOFs (**ftw**-MOF-2 and **ftw**-MOF-3, see Fig. 2 and 3). The versatility of this platform was evidenced by the construction of **ftw**-MOFs where the central core of the ligand was functionalized/decorated (*i.e.* three related ligands having a central phenyl (**ftw**-MOF-2), naphthalene (**ftw**-MOF-2 (Naphth)), or anthracene (**ftw**-MOF-2 (Anth)) based core, see Fig. 2 and 3).

## Results and discussion

### **ftw**-MOF based on a geometrically square ligand

In our efforts to target the first example of a RE **ftw**-MOF, we designed a flexible tetracarboxylate ligand, 1,2,4,5-tetrakis[(4-carboxy)phenoxymethyl]benzene (TetPOMB) (Fig. 2), which can act as a square/quadrangular MBB. The selection of TetPOMB was based on the recognized versatility of similar ligands with a central phenyl ring as the core, with ether linkages to benzene carboxylic acid moieties.^20^–^22^ The flexibility of this type of ligand permits adoption of the necessary geometry (a square) to allow the formation of a MOF with the ideal **ftw** topology.

As anticipated, based on previous work from our group, the *in situ* formation of the 12-c rare earth MBB was facilitated by the addition of excess 2-fluorobenzoic acid (2-FBA), a modulator and a directing agent for the *in situ* formation of highly-connected polynuclear carboxylate-based clusters.^15^ This allowed for the synthesis of yttrium, terbium, and ytterbium **ftw**-MOF-1 analogs. Y-**ftw**-MOF-1 was structurally characterized by single-crystal X-ray diffraction (SCXRD) and phase purity of the Y, Tb and Yb analogs was confirmed by Whole Profile Pattern Matching using the Le Bail method (Fig. S4a, ESI†, Table 1).^23^

**Table 1:** Crystallographic data and structural refinement

Compound	Y-**ftw**-MOF-1	Y-**ftw**-MOF-2	Y-**ftw**-MOF-3
Formula	C_114_H_86_O_52_Y_6_	C_138_H_78_O_35_Y_6_	C_162_H_78_O_32_Y_6_
FW	2821.29	2829.46	3069.70
Crystal system	Cubic	Cubic	Cubic
Space group	*Pm*3*m*	*Im*3	*Im*3
*a* (Å)	19.3042(5)	40.048(3)	48.111(16)
*V* (Å^3^)	7193.8(3)	64 229(13)	111 362(112)
*Z*, *D*_cal_ (g cm^–3^)	1	8	8
*θ*_max_ (°)	131.92	88.92	72.688
*R*_int_	0.0577	0.1045	n/a^*a*^
*R*_1_ (*I* > 2*σ*(*I*_0_))	0.0834	0.0983	0.0779
w*R*_2_ (all data)	0.2626	0.2493	0.2196
GOF	1.189	1.174	0.871
Δ*ρ*_max_/Δ*ρ*_min_ (e Å^–3^)	0.60/–0.88	2.27/–0.83	1.01/–0.63

^*a*^ *R*_int_ is missing due to the fact that de-twinned data have been imported from an FCF file (containing merged data) generated by Shelxl-2013 with LIST 8 instruction.

**Fig. 4:**
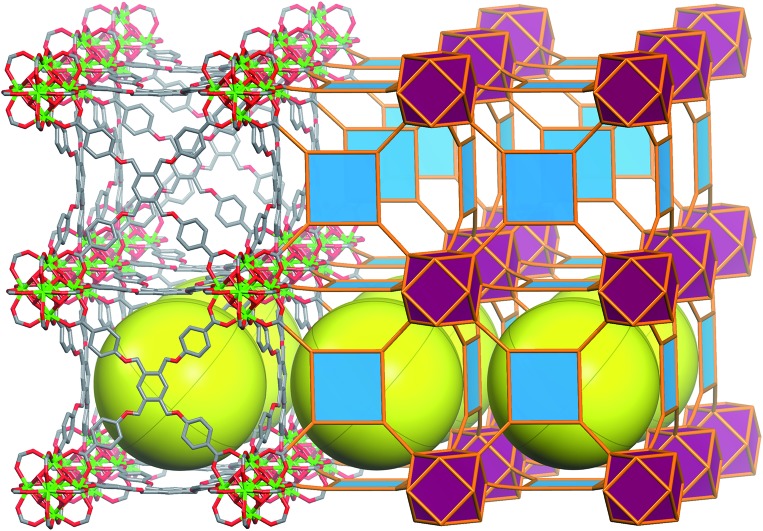
Molecular and vertex figure representation of **ftw**-MOF-1, yellow spheres denote the open space within each cage; (top left) TetPOMB ligand coordinated to four 12-connected hexanuclear clusters and (bottom right) vertex figure representation.^11^

SCXRD studies revealed that **ftw**-MOF-1 crystallized in the cubic *Pm*3*m* space group and was formulated as |(DMA)_2_|[(Y_6_(μ_3_-OH)_8_(H_2_O)_6_)(TetPOMB)_3_]·(solv)_*x*_; DMA = dimethylammonium cations, solv = solvent. Topological analysis confirmed that the material had the expected **ftw** topology (Fig. S1, ESI†). The **ftw**-MOF-1 structure can be viewed as a primitive cubic packing of the 12-c rare earth MBBs, which are positioned in the center of the unit cell. The 12-c rare earth MBBs are connected through 12 bis-monodentate carboxylates from the 12 separate TetPOMB ligands (Fig. 4), thus forming one central cubic cage ∼19 Å in diameter that is delimited by six TetPOMB ligands (Fig. 4). Due to the flexibility of the ether linkages of the ligand, its central phenyl core is disordered over four positions in the crystal structure. Preliminary CO_2_ and N_2_ adsorption studies revealed that **ftw**-MOF-1 was not permanently porous using conventional activation procedures. In fact, numerous attempts to activate the **ftw**-MOF-1 samples for gas sorption analysis resulted in loss of crystallinity. We attributed this lack of robustness to the flexibility in the ligand, which plausibly resulted in the collapse of the material upon guest molecule removal.

### **ftw**-MOF based on rigid and expanded rectangular ligand

Further structural examination revealed that a MOF with **ftw** topology could potentially be accessed using rectangular ligands. A rotation of the inorganic 12-c MBB, as well as alternation in the orientation of the 4-c ligand (the organic MBB), appeared to be required in order to accommodate the lower symmetry rectangular ligands for the construction of **ftw**-MOFs (Fig. 2 and S1–S3, ESI†). A similar phenomenon was reported recently in structurally related Zr **ftw**-MOFs.^13^ Convincingly, in order to synthesize a more rigid and permanently porous **ftw**-MOF, a rigid expanded analog of the TetPOMB linker was synthesized: 3,3′′,5,5′′-tetrakis(4-carboxyphenyl)-*p*-terphenyl (TCPT, Fig. 2 and 3). Under similar reaction conditions, including the use of 2-FBA, crystalline yttrium, ytterbium, and terbium **ftw**-MOF-2 analogs were synthesized. Y-**ftw**-MOF-2 was characterized by SCXRD, and the phase purity of the Y, Tb, and Yb analogs was confirmed by Whole Profile Pattern Matching using the Le Bail method (Fig. S4a and S4b, ESI†).^23^ SCXRD studies revealed that Y-**ftw**-MOF-2 crystallized in the cubic *Im*3 space group, and was formulated as |(DMA)_2_|[(Y_6_(μ_3_-OH)_8_(H_2_O)_6_)(TCPT)_3_]·(solv)_*x*_. Topological analysis confirmed that the material was based on an augmented **ftw** net (**ftw**-a), the expected (4,12)-c **ftw** topology when considering the ligand as a 4-c node. Alternatively, and from a purely topological point of view, the resultant structure can be described as a trinodal (3,3,12)-c net with transitivity [3443], given that the 4-c ligand comprises two 3-c triangular nodes.^24^ Consequently, the employment of a quadrangular 4-c ligand, based on linked 3-c triangular building units, permits us to disclose a novel (3,3,12)-c net derived from the parent **ftw**-net with a new topology yet to be recognized (Fig. S3, ESI†).^10^,^13^ Accordingly, we assigned the three letter code **kle** (KAUST Luebke Eddaoudi) to the **ftw**-derived net.^10^

**Fig. 5:**
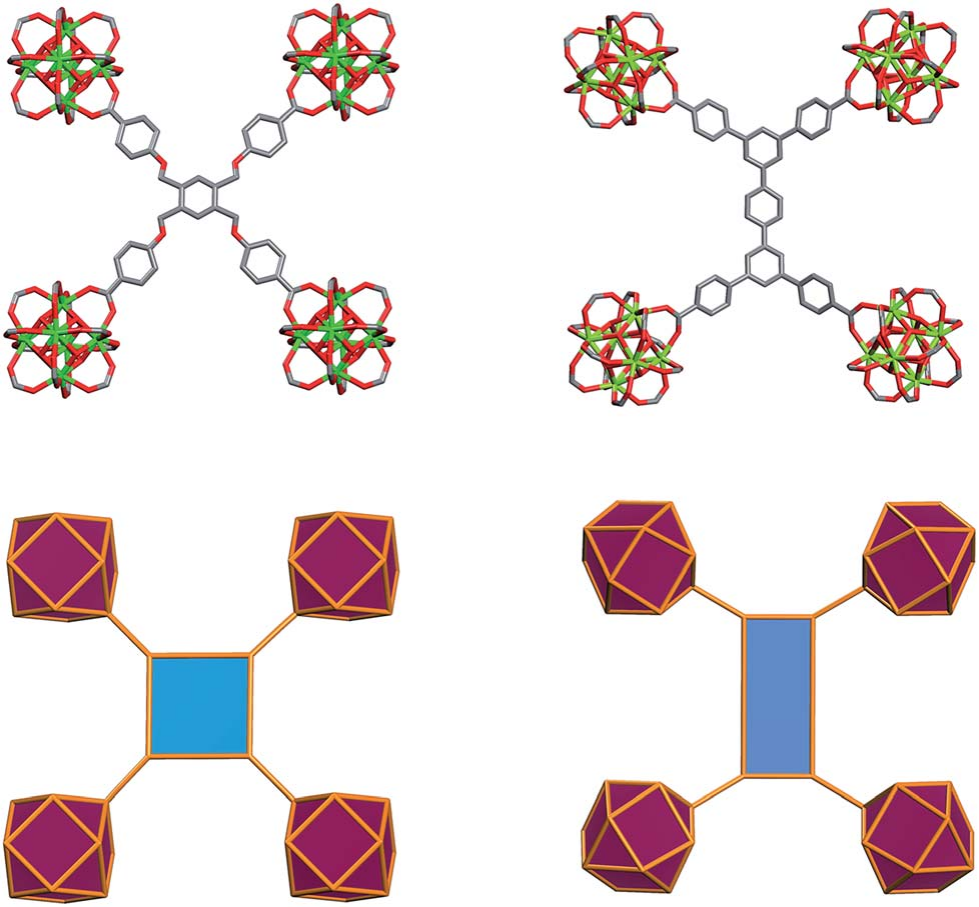
Illustration of the ligand geometry effect on the orientation of the 12-c cluster in **ftw**-fragment of **ftw**-MOF-1 (left) and **ftw**-MOF-2 (right).^11^

**Fig. 6:**
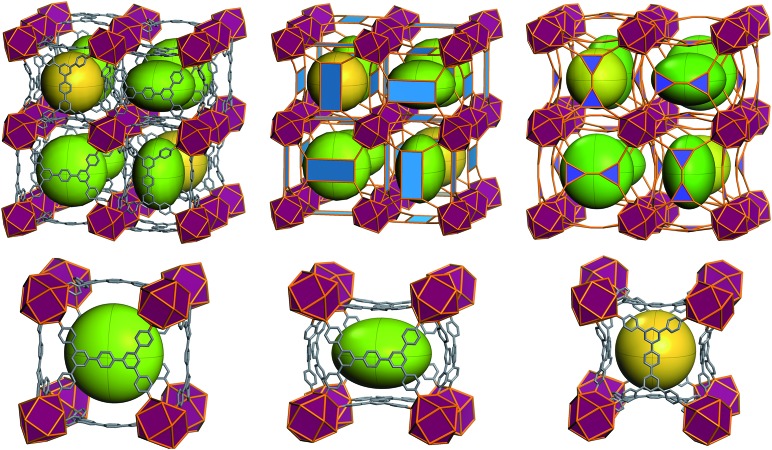
(Top) Representation of the **ftw**-MOF-2 framework: green ellipsoids represent the larger asymmetrical cage; yellow spheres represent the smaller symmetrical cages. Purple polyhedra represent the rare earth hexanuclear MBB; blue polygons represent the 4-connected quadrangular organic MBB. (Bottom left and center) Diagram of the asymmetrical cage along two axes; (bottom right) diagram of the symmetrical cage.

The **ftw**-MOF-2 structure is composed of ligands with a *para*-terphenyl core covalently linked in the 3,3′′,5,5′′ positions to the *para* position of benzoate moieties, thus forming a rectangular 4-c MBB (Fig. 5). A cubic cage ∼12 Å in diameter, with *T*_h_ symmetry, is delimited by six ligands: the carboxylates coordinate to the four 12-c RE MBBs positioned at the vertices of the cube face on which the ligand is situated. Compared to the parent **ftw**-MOF-1, the 12-c MBBs (the hexanuclear [RE_6_(OH)_8_(O_2_C–)_12_] carboxylate-based clusters) are rotated ∼15° out of perfect alignment along all three axes (*i.e.* in the *x*, *y* and *z* directions), adapting the orientation to allow for the accommodation of the rectangular ligand (Fig. 5). Consequently, the ligands on the faces of the central cage are all puckered inward. The ligands in the *yz*-plane are oriented lengthwise in the *z*-direction; the ligands in the *xy*-plane are oriented lengthwise in the *y*-direction; and the ligands in the *xz*-plane are oriented lengthwise in the *x*-direction (Fig. 6 and S9, ESI†). Adjacent to, and sharing one face of the cubic cage, are six larger cages with *D*_2h_ symmetry. This larger cage (21 Å × 21 Å × 12 Å) is a distorted cube delimited by six ligands. The ligands on any two parallel faces are oriented in the same direction with respect to one another and puckered inward; the remaining four faces are puckered outward and the ligands are oriented end to end lengthwise (Fig. 6 and S10, ESI†).

Evidently, from a structural perspective, further isoreticular expansion of the **ftw**-MOF platform could be accomplished through extension of the arms of the TCPT ligand. Indeed, Y-**ftw**-MOF-3 was synthesized from 3,3′′,5,5′′-tetrakis[2-(4-ethoxycarbonylphenyl)ethynyl]-*p*-terphenyl (TCEPT) with alkyne moieties extending the four arms of the TCPT ligand (Fig. 2 and 3). The SCXRD studies revealed that Y-**ftw**-MOF-3 crystallized in the cubic *Im*3 space group and was formulated as |(DMA)_2_|[(Y_6_(μ_3_-OH)_8_(H_2_O)_6_)(TCEPT)_3_]·(solv)_*x*_. Structural analysis revealed that connectivity and ligand orientation in the isoreticular Y-**ftw**-MOF-3 was similar to the aforementioned Y-**ftw**-MOF-2. The minimum diameter of the central highly symmetric cubic cage was ∼19 Å (Fig. S11, ESI†) and the cuboidal cage had dimensions of approximately 23 Å × 22 Å × 18 Å (Fig. S12, ESI†). Notably, the cages in Y-**ftw**-MOF-3 were relatively less distorted than in Y-**ftw**-MOF-2, as the geometry of the TCEPT ligand is closer to a square than TCPT. This resulted in less strain and torsion as well as a slightly less pronounced rotation of the 12-c MBB. In comparison to the parent **ftw**-MOF-1, the MBB was rotated only by ∼10° out of perfect alignment along all three axes (*i.e.* in the *x*, *y* and *z* directions).

### **ftw**-MOF functionalization *via* ligand decoration

To illustrate the tunability of the **ftw**-MOF platform, and subsequently investigate the impact of additional aromatic functionality on the associated sorption properties, naphthalene and anthracene functionalized analogs of **ftw**-MOF-2 were synthesized (Y-**ftw**-MOF-2 (Naphth) and Y-**ftw**-MOF-2 (Anth)). In these analogs, the central 1,4-substituted phenyl ring of TCPT was replaced with a 1,4-substituted naphthalene core and a 9,10-substituted anthracene core (Fig. 2 and 3).

SCXRD studies revealed that Y-**ftw**-MOF-2 (Naphth) crystallized in the cubic *Im*3 space group. A modeled structure, based on PXRD and a unit cell determination from single crystal data, was formulated as |(DMA)_2_|[(Y_6_(μ_3_-OH)_8_(H_2_O)_6_)(TCDPN)_3_]·(solv)_*x*_. Experimental PXRD patterns matched the calculated PXRD from the modeled structure. This confirmed the attainment of the anticipated isoreticular structure, Y-**ftw**-MOF-2 (Naphth). The structure of the Y-**ftw**-MOF-2 (Naphth) analogue differs from the parent **ftw**-MOF-2 with regard to the orientation of the bicyclic central core of the ligands: the bulkier naphthalene core is rotated 90° out of plane (Fig. S13 and S14, ESI†). Similarly, the resultant functionalized **ftw**-MOFs enclose two distinct cages. Namely, a highly symmetric cubic cage, with *T*_h_ symmetry, delimited by six ligands which are puckered outward with the bulk of the naphthalene core pointed toward the center of the cage, and with a minimum cage diameter of ∼9.5 Å. The 12-c MBBs are rotated ∼13° out of perfect alignment along all three axes (*i.e.* in the *x*, *y* and *z* directions). Adjacent to, and sharing one face of, the cubic cage, are six symmetry related cuboidal cages with *D*_2h_ symmetry. These cages, with minimum diameters of approximately 14 Å, 13 Å and 10 Å, are delimited by six ligands, two of which are puckered outward having the bulk of the naphthalene core pointing inward, and the other four ligands are puckered inward with the bulk of the naphthalene core pointing outward into the adjacent cage.

Confirmation of the successful synthesis of Y-**ftw**-MOF-2 (Anth) was only possible by unit cell indexing from a poor quality highly twinned crystal and further comparing the experimental powder pattern to a modeled and geometry optimized Y-**ftw**-MOF-2 (Anth) structure (Fig. S7, ESI†). The determined structure was consistent with the Y-**ftw**-MOF-2 (Naphth) structure with regard to connectivity and ligand geometry. Accordingly, the Y-**ftw**-MOF-2 (Anth) structure was modeled based on Y-**ftw**-MOF-2 (Naphth), and further functionalized and geometry optimized in Materials Studio. The Y-**ftw**-MOF-2 (Anth) encloses two unique cages. The first is a highly symmetric cubic cage, with *T*_h_ symmetry and a minimum diameter of ∼7 Å (Fig. S15, ESI†). This cage is delimited by six ligands which are puckered inward; the anthracene core is rotated perpendicular to the plane of ligand. The 12-c MBBs are oriented similarly to Y-**ftw**-MOF-2 (Naphth), *i.e.* rotated ∼13° out of perfect alignment along all three axes (*i.e.* in the *x*, *y* and *z* directions). The second cage is a cuboidal cage with *D*_2h_ symmetry (Fig. S16, ESI†), and minimum diameters of approximately 14 Å, 12 Å and 5 Å. It is delimited by six ligands, two of which are puckered inward while the other four ligands are puckered outward: the anthracene core is rotated and adopts an orientation perpendicular to the plane of the ligand.

### Gas sorption analysis

As envisioned, the inherent rigidity of the TCPT-based ligands led to the attainment of **ftw**-MOFs with permanent porosity. Ar adsorption studies at 87 K showed that Y-**ftw**-MOF-2, Y-**ftw**-MOF-2 (Naphth) and Y-**ftw**-MOF-2 (Anth) exhibited a fully reversible Type-I isotherm (Fig. S20a, S21a and S22a, ESI†), characteristic of microporous materials. Table 2 summarizes the apparent BET and Langmuir surface areas and pore volumes estimated from the Ar adsorption isotherms. The pore size distribution (PSD) for each of the Y-**ftw**-MOF-2 analogs (Fig. S20b, S21b and S22b, ESI†) was determined and found to be in good agreement with pore size derived from the corresponding structures.

**Table 2:** Porosity information derived from Ar low pressure adsorption data for **ftw**-MOF-2 compounds at 87 K

Compound	Y **ftw**-MOF-2	Y-**ftw**-MOF-2 (Naphth)	Y-**ftw**-MOF-2 (Anth)
BET (m^2^ g^–1^)	3690	3040	2100
Langmuir (m^2^ g^–1^)	3740	3100	2500
Free volume	72%	68%	66%
Experimental PV (cm^3^ g^–1^)	1.26	1.05	0.79
Crystallographic PV (cm^3^ g^–1^)	1.26	1.11^*a*^	1.03^*a*^

^*a*^Based on modeled structure from unit cell parameters.

Similar to the recently reported rare earth based (3,18)-connected **gea**-MOF-1,^25^ Y-**ftw**-MOF-2 maintained its crystallinity up to 400 °C (Fig. S5a, ESI†) and retained its optimal porosity upon heating beyond 200 °C *in vacuo*, indicative of a high degree of thermal stability. It is worth mentioning that most other examples of highly thermally stable MOFs are not often supported by porosity measurements after such high temperature treatment.

In order to assess the structure–properties relationship for Y-**ftw**-MOF-2, gas adsorption measurements were undertaken for CO_2_, N_2_, CH_4_ and H_2_ at 298 K at low and high pressures. H_2_ and CO_2_ showed weak interactions with Y-**ftw**-MOF-2, as evidenced by their relatively low isosteric heats of adsorption, *Q*_st_: 5.7 and 27 kJ mol^–1^ for H_2_ and CO_2_, respectively, at low loading. The *Q*_st_ values were determined from variable temperature adsorption isotherms at 258, 268 and 278 K for CO_2_ (Fig. S23 and S24, ESI†) and at 77 K and 87 K for H_2_ (Fig. S27, ESI†).

The CO_2_ adsorption isotherm at high pressure (Fig. S30a and S31a, ESI†) indicated a nearly full saturation of the pore system at 25 bar and 273 K (24.62 mmol g^–1^). This corresponds to a pore volume of 1.17 cm^3^ g^–1^ that is in close agreement with the theoretical pore volume calculated from the Ar isotherm at 87 K (1.26 cm^3^ g^–1^). The volumetric CO_2_ uptake at 25 bar and 298 K (278 cm^3^ (STP) cm^–3^) was slightly lower (∼7%) than UTSA-20 (ref. 7) (300 cm^3^ (STP) cm^–3^) under similar conditions.

Examination of absolute gravimetric (mmol g^–1^) and volumetric (cm^3^ (STP) cm^–3^) CH_4_ uptake at intermediate and high pressures revealed that Y-**ftw**-MOF-2 adsorbed 32 and 174 cm^3^ (STP) cm^–3^ of CH_4_ at 5 and 50 bar, respectively (Fig. S30b, ESI†). The resultant CH_4_ working storage capacity, assuming 50 bar as the highest adsorption pressure and 5 bar as the lowest desorption pressure (following the requirement of the engine methane injection pressure),^26^ is *ca.* 142 cm^3^ (STP) cm^–3^ (Table S4, ESI†). This volumetric working capacity, based on the crystallographic density of Y-**ftw**-MOF-2, is significantly higher than UTSA-20 (ref. 7) (80 cm^3^ (STP) cm^–3^) and higher than PCN-14 (ref. 5) (130 cm^3^ (STP) cm^–3^) but still lower than the working CH_4_ storage capacity calculated for NU-125 (ref. 26) (165 cm^3^ (STP) cm^–3^), **tbo**-MOF-2 (ref. 20) (152 cm^3^ (STP) cm^–3^) and other recently reported MOFs^4^,^27^,^28^ (Table S4, ESI†). The CO_2_/N_2_ and CO_2_/CH_4_ selectivity, determined using ideal adsorption solution theory (IAST), was moderate in comparison to other best performing MOFs and other classes of CO_2_ separation agents (Fig. S30b, ESI†).

**Fig. 7:**
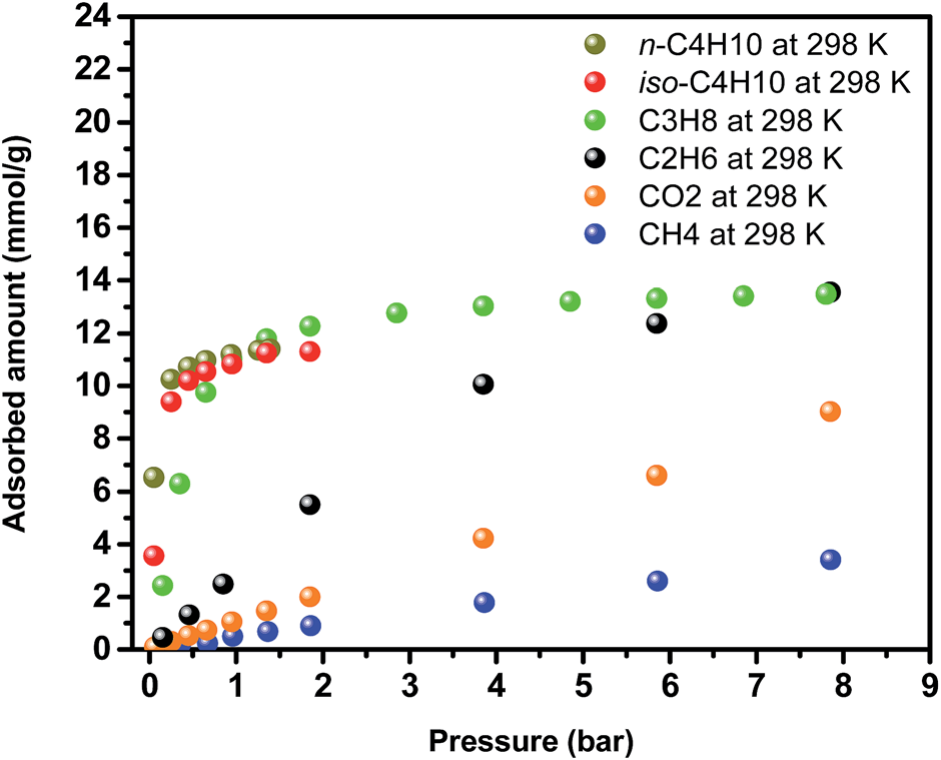
Single-gas adsorption isotherms for CO_2_, CH_4_, C_2_H_6_, C_3_H_8_, *n*-C_4_H_10_ and iso-C_4_H_10_ on Y-**ftw**-MOF-2.

Encouraged by the higher CH_4_ uptake of Y-**ftw**-MOF-2, adsorption of larger and relatively highly polarizable probe molecules such as C_2_H_6_, C_3_H_8_ and *n*-C_4_H_10_ and iso-C_4_H_10_ (C_2+_) were also investigated (Fig. 7). The results were compared with the corresponding CO_2_ and CH_4_ adsorption data and subsequently the C_2+_/CH_4_ and *n*-C_4_H_10_/iso-C_4_H_10_ separation factors were derived (Fig. S32, ESI†). Interestingly, C_2+_ adsorption isotherms were much steeper at low pressures than for CH_4_ and CO_2_, indicative of a plausibly higher affinity of Y-**ftw**-MOF-2 for C_2+_. Examination of single adsorption isotherm data using IAST confirmed the high C_2+_/CH_4_ selectivities, namely at 1 bar the selectivity was *ca.* 50 for C_3_H_8_/CH_4_ (5/95) and *ca.* 450 for *n*-C_4_H_10_/CH_4_ (5/95) (Fig. S32a, ESI†). In addition, Y-**ftw**-MOF-2 exhibited selectivities of 6 and 2.5 in 50/50 mixtures of *n*-C_4_H_10_/C_3_H_8_ and *n*-C_4_H_10_/iso-C_4_H_10_, respectively, at 1 bar and 298 K (Fig. S32b, ESI†). Accordingly, Y-**ftw**-MOF-2 offers promise as a separation agent, particularly for *n*-C_4_H_10_ in CH_4_ containing gas streams.

Similar to Y-**ftw**-MOF-2, weak CO_2_ and H_2_ interactions were observed for Y-**ftw**-MOF-2 (Naphth) and Y-**ftw**-MOF-2 (Anth), where the *Q*_st_ values at low loading were 25 and 22 kJ mol^–1^ CO_2_ and 6.4 and 6.9 kJ mol^–1^ for H_2_, respectively (Fig. S25, S26, S28 and S29, ESI†).

In order to assess the effect of the bulkier naphthalene core on the CO_2_ and C_2+_ uptakes, gas sorption studies were performed on Y-**ftw**-MOF-2 (Naphth). Based on single component adsorption isotherm data, the added functionality (bulkier core, reduced pore size) had a minimal impact on the adsorption properties and selectivity toward CO_2_ and C_2+_ (Fig. S33–S37, ESI†). It is foreseeable that the inclusion of aliphatic or polarized groups may offer more promising results.^15^ Accordingly, further experiments are ongoing to construct **ftw**-MOFs decorated with suitable functionalities for gas separation. Regarding **ftw**-MOF-3, work is in progress to determine the optimum activation conditions prior to performing detailed sorption studies.

## Conclusion

A newly isolated 12-c RE MBB, RE_6_(μ_3_-OH)_8_(O_2_C–)_12_, was successfully employed to construct a (4,12)-c MOF platform based on **ftw** topology, illustrating the importance of highly-connected building blocks for the practice of reticular chemistry where the assembly of desired MOFs is guided by the limited number of related highly-connected nets. This unique RE-based **ftw**-MOF platform is amenable to functionalization and/or expansion, as illustrated by the synthesis of five isoreticular **ftw**-MOFs. **ftw**-MOF-1, based on a square-like ligand, was synthesized and its adaptability to various rare earth metals was evidenced by the formation of its isostructures. Extending the central core, through use of a rigid expanded tetracarboxylate, permitted the synthesis of permanently porous isoreticular **ftw**-MOFs. Specifically, the Y-**ftw**-MOF-2 based on the TCPT ligand exhibited a record-high specific surface area (*e.g.* 3690 m^2^ g^–1^) for RE-based MOFs; it also offers the potential for sorption based separation of hydrocarbons. Further expansion *via* lengthening the arms of TCPT lead to a theoretically more open **ftw**-MOF structure (**ftw**-MOF-3) based on a relatively more symmetrical ligand. Finally the **ftw**-MOF structural features permitted additional functionalization in the central core, as evidenced by the successful intra-framework introduction of naphthalene and anthracene groups.

## Experimental

### Synthesis of compounds

#### Synthesis of Y-**ftw**-MOF-1

To a 23 ml glass scintillation vial containing TetPOMB (11.5 mg, 0.017 mmol) was added 0.5 ml 0.068 M Y(NO_3_)_3_·6H_2_O in DMF (0.034 mmol), 0.5 ml 1 M 2-fluorobenzoic acid (0.5 mmol) in DMF and 1.0 ml DMF. The vial was sealed and placed into a preheated oven at 115 °C for 2 days. Colorless cubic block shaped crystals were obtained. Yield: 4.2 mg.

#### Synthesis of Yb-**ftw**-MOF-1

To a 23 ml glass scintillation vial containing TetPOMB (11.5 mg, 0.017 mmol) was added 0.5 ml 0.068 M Yb(NO_3_)_3_·6H_2_O in DMF (0.034 mmol), 0.5 ml 1 M 2-fluorobenzoic acid (0.5 mmol) in DMF and 1.0 ml DMF. The vial was sealed and placed into a preheated oven at 115 °C for 2 days. Colorless cubic block shaped crystals were obtained. Yield: 5.6 mg.

#### Synthesis of Tb-**ftw**-MOF-1

To a 23 ml glass scintillation vial containing TetPOMB (11.5 mg, 0.017 mmol) was added 0.5 ml 0.068 M Tb(NO_3_)_3_·5H_2_O in DMF (0.034 mmol), 0.5 ml 1 M 2-FBA (0.5 mmol) in DMF and 1.0 ml DMF. The vial was sealed and placed into a preheated oven at 115 °C for 2 days. Colorless cubic block shaped crystals were obtained. Yield: 6.4 mg.

#### Synthesis of Y-**ftw**-MOF-2

To a 23 ml glass scintillation vial containing TCPT (8 mg, 0.011 mmol) was added 0.5 ml 0.068 M Y(NO_3_)_3_·6H_2_O in DMF (0.034 mmol), 1.0 ml 2 M 2-FBA (2.0 mmol) in DMF and 1.0 ml DMF. The vial was sealed and placed into a preheated oven at 115 °C for 24 h. Colorless cubic block shaped crystals were obtained. Yield: 8.3 mg.

#### Synthesis of Yb-**ftw**-MOF-2

To a 23 ml glass scintillation vial containing TCPT (8 mg, 0.011 mmol) was added 0.5 ml 0.068 M Yb(NO_3_)_3_·6H_2_O in DMF (0.034 mmol), 1.0 ml 2 M 2-FBA (2.0 mmol) in DMF and 1.0 ml DMF. The vial was sealed and placed into a preheated oven at 115 °C for 24 h. Colorless cubic block shaped crystals were obtained. Yield: 5.6 mg.

#### Synthesis of Tb-**ftw**-MOF-2

To a 23 ml glass scintillation vial containing TCPT (8 mg, 0.011 mmol) was added 0.5 ml 0.068 M Tb(NO_3_)_3_·5H_2_O in DMF (0.034 mmol), 1.0 ml 2 M 2-FBA (2.0 mmol) in DMF and 1.0 ml DMF. The vial was sealed and placed into a preheated oven at 115 °C for 24 h. Colorless cubic block shaped crystals were obtained. Yield: 5.8 mg.

#### Synthesis of Y-**ftw**-MOF-2 (Naphth)

To a 23 ml glass scintillation vial containing TCDPN (8.5 mg, 0.011 mmol) was added 0.5 ml 0.068 M Y(NO_3_)_3_·6H_2_O in DMF (0.034 mmol), 1.0 ml 2 M 2-FBA (2.0 mmol) in DMF and 0.5 ml DMF. The vial was sealed and placed into a preheated oven at 115 °C for 24 h. Colorless cubic block shaped crystals were obtained. Yield: 8.5 mg.

#### Synthesis of Y-**ftw**-MOF-2 (Anth)

To a 23 ml glass scintillation vial containing TCDPA (9 mg, 0.011 mmol) was added 0.5 ml 0.068 M Y(NO_3_)_3_·6H_2_O in DMF (0.034 mmol), 1.0 ml 2 M 2-FBA (2.0 mmol) in DMF and 0.5 ml DMF. The vial was sealed and placed into a preheated oven at 115 °C for 24 h. Pale yellow cubic block shaped crystals were obtained. Yield: 8.0 mg.

#### Synthesis of Y-**ftw**-MOF-3

To a 23 ml glass scintillation vial containing TCEPT (9 mg, 0.011 mmol) was added 0.5 ml 0.068 M Y(NO_3_)_3_·6H_2_O in DMF (0.034 mmol), 0.5 ml 2 M 2-FBA (2.0 mmol) in DMF and 2.0 ml DMF. The vial was sealed and placed into a preheated oven at 115 °C for 24 h. The as synthesized pale yellow cubic block shaped crystals were obtained. The product was washed with copious amounts of DMF to remove impurities. Yield: 4.3 mg.

## Supplementary Material

Supplementary information

Crystal structure data
