# Supplementary material for: Versatile rare earth hexanuclear clusters for the design and synthesis of highly-connected ftw-MOFs
Source: Chem Sci. 2015 Apr 15;6(7):4095–102. doi: 10.1039/c5sc00614g (PMC5707464; doi:10.1039/c5sc00614g)
Supplement: Supplementary file 1 [file SC-006-C5SC00614G-s001.pdf]

Electronic Supplementary Material (ESI) for Chemical Science.
This journal is © The Royal Society of Chemistry 2015

Supporting Information

Versatile Rare Earth Hexanuclear Clusters for the Design and Synthesis of Highly-connected **ftw**-MOFs

Ryan Luebke,[a] Youssef Belmabkhout,[a] Łukasz J. Weseliński,[a] Amy J. Cairns,[a] Mohamed Alkordi,[a] George Norton,[b] Łukasz Wojtas,[b] Karim Adil[a] and Mohamed Eddaoudi[a]

[a] Functional Materials Design, Discovery & Development Research Group (FMD^3), Advanced Membranes & Porous Materials Center, Division of Physical Sciences and Engineering, King Abdullah University of Science and Technology (KAUST), Thuwal 23955-6900, Kingdom of Saudi Arabia

[b] Department of Chemistry, University of South Florida, 4202 East Fowler Avenue, Tampa, Florida 33620, United States

Table of Contents

Topological Analysis: 2
(4,12)-Connected **ftw**-MOFs 2
Topological analyses were performed using TOPOS.[1] 5
X-ray Crystallography and Structural Analysis: 7
Powder X-ray Diffraction 7
Single Crystal X-ray Crystallography 11
Structure Analysis and Ligand Geometry: 13
Geometric and Molecular Representation of Cages: 13
Diagrams of the cages in **ftw**-MOF-2: 13
Diagrams of the cages in **ftw**-MOF-3: 14
Diagrams of the cages in **ftw**-MOF-2 (Naphth): 15
Diagrams of the modelled cages in **ftw**-MOF-2 (Anth): 16
Ligand Geometry Analysis: 17
Thermal Gravimetric Analysis: 18
Gas Sorption Analysis: 20
Low Pressure Gas Adsorption Measurements: 20
High-Pressure Gas Adsorption Measurements 25
Organic Synthesis: 31

Topological Analysis:

(4,12)-Connected ftw-MOFs

The MOF described as **ftw**-MOF-1 is composed of 4 and 12-connected molecular building blocks (MBBs) (Figure S1). The flexibility of the methoxy linkages allows the ligand to adopt a geometrically square conformation. This geometrically square ligand combined with the 12-connected metal cluster results unambiguously in a MOF with the **ftw**-a net.

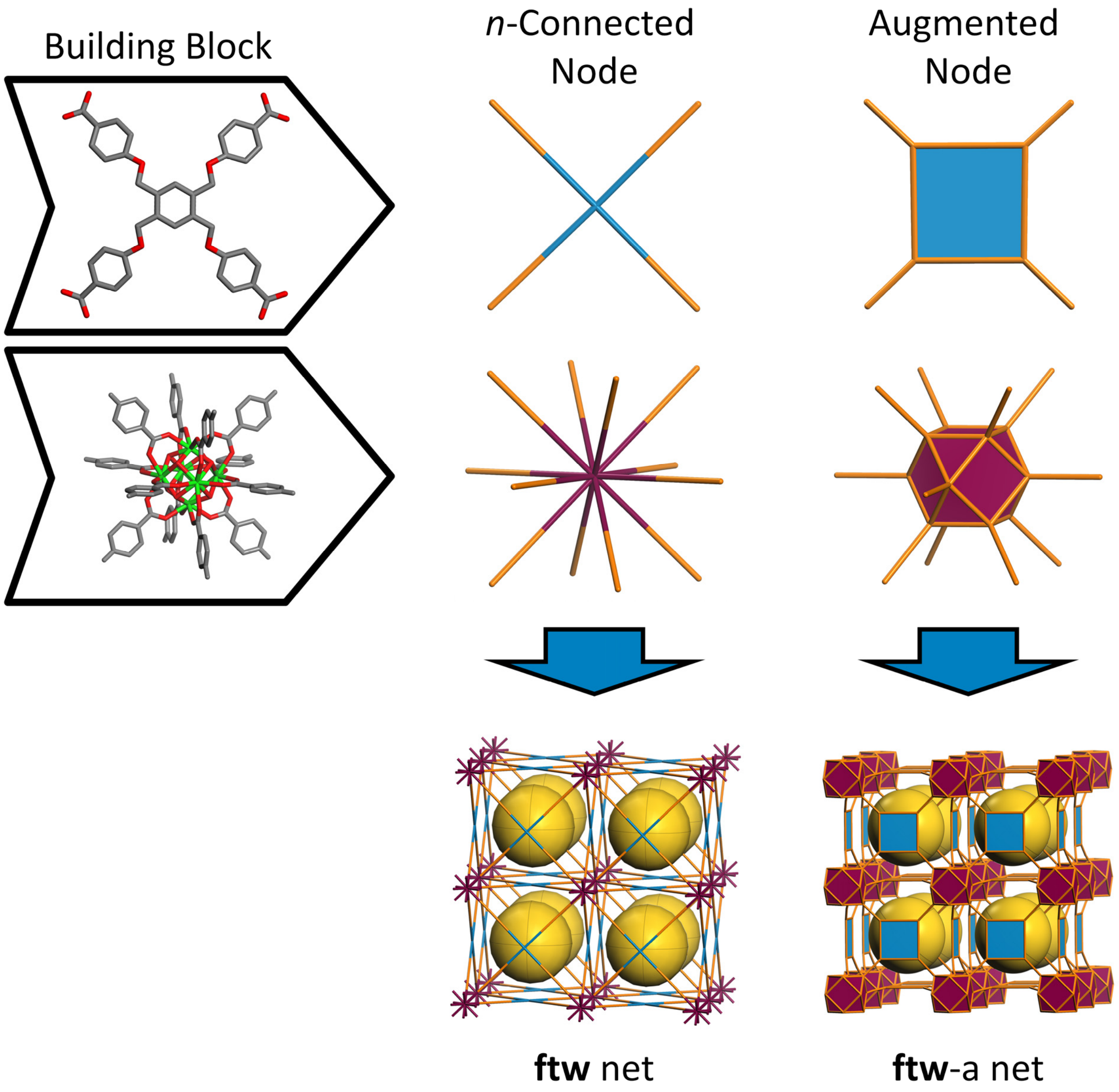

Figure S1: Molecular Building Blocks (MBBs) and geometric representation of **ftw**-MOF-1 resulting in **ftw** and **ftw-**a nets.

In contrast, **ftw**-MOF-2 is composed of an elongated quadrangular ligand, which can be interpreted in several ways (Figure S2). The first interpretation is as a single 4-connected node, which similar to **ftw**-MOF-1, results in the **ftw**-a net. Alternatively and from a purely topological point of view, the resultant structure can be described as a topology, which we designate as **kle,** based on a trinodal (3,3,12)-c net given that the 4-c ligand comprises two 3-c triangular nodes.. This network topology is most closely related to **ttv**, in which the orientation of the linked three connected nodes is in a single orientation in a crystallographic plane, whereas, in the present **kle** topology, the orientation alternates between two orientations.

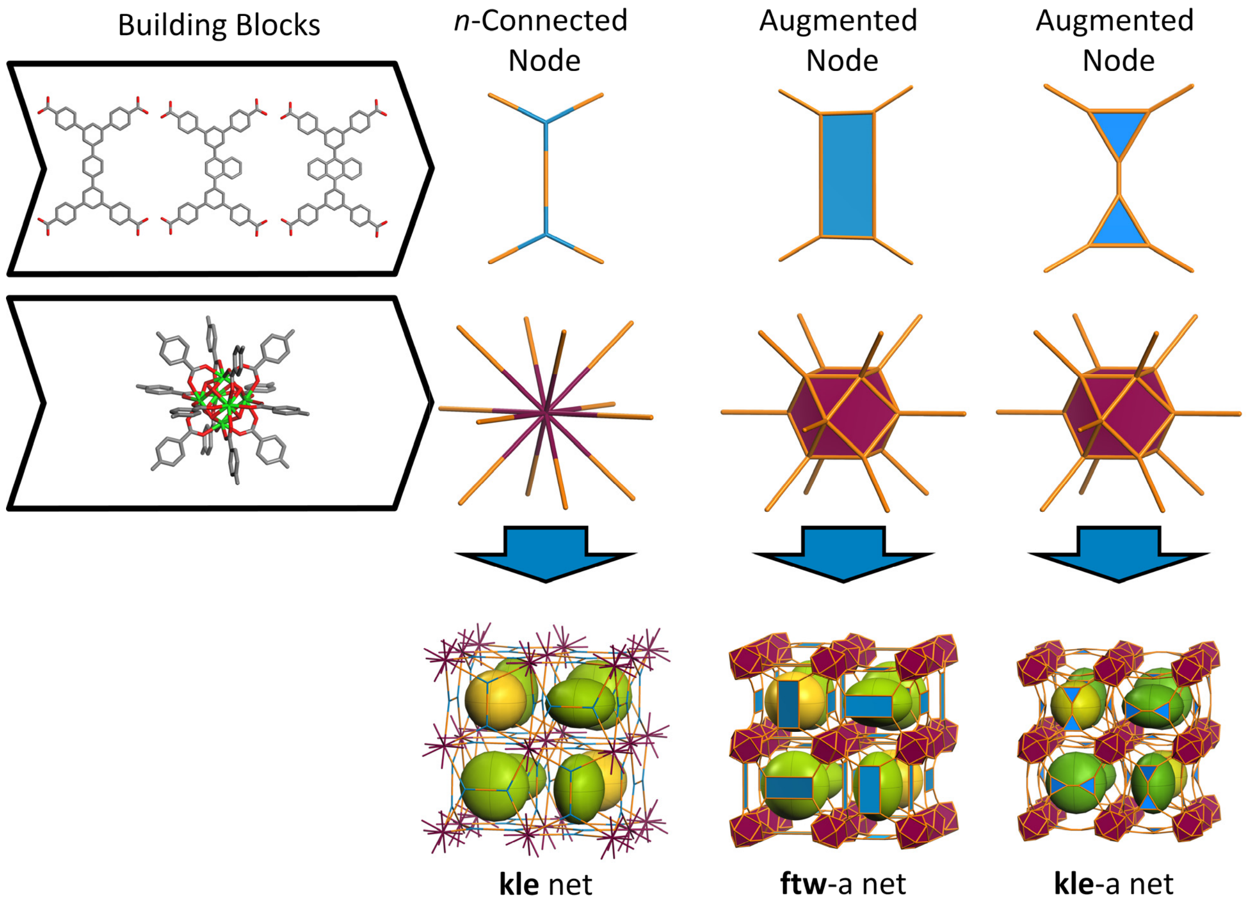

Figure S2: Molecular Building Blocks (MBBs) and geometric representation of the **ftw**-MOF-2 analogues resulting in **kle** and **ftw**-a nets.

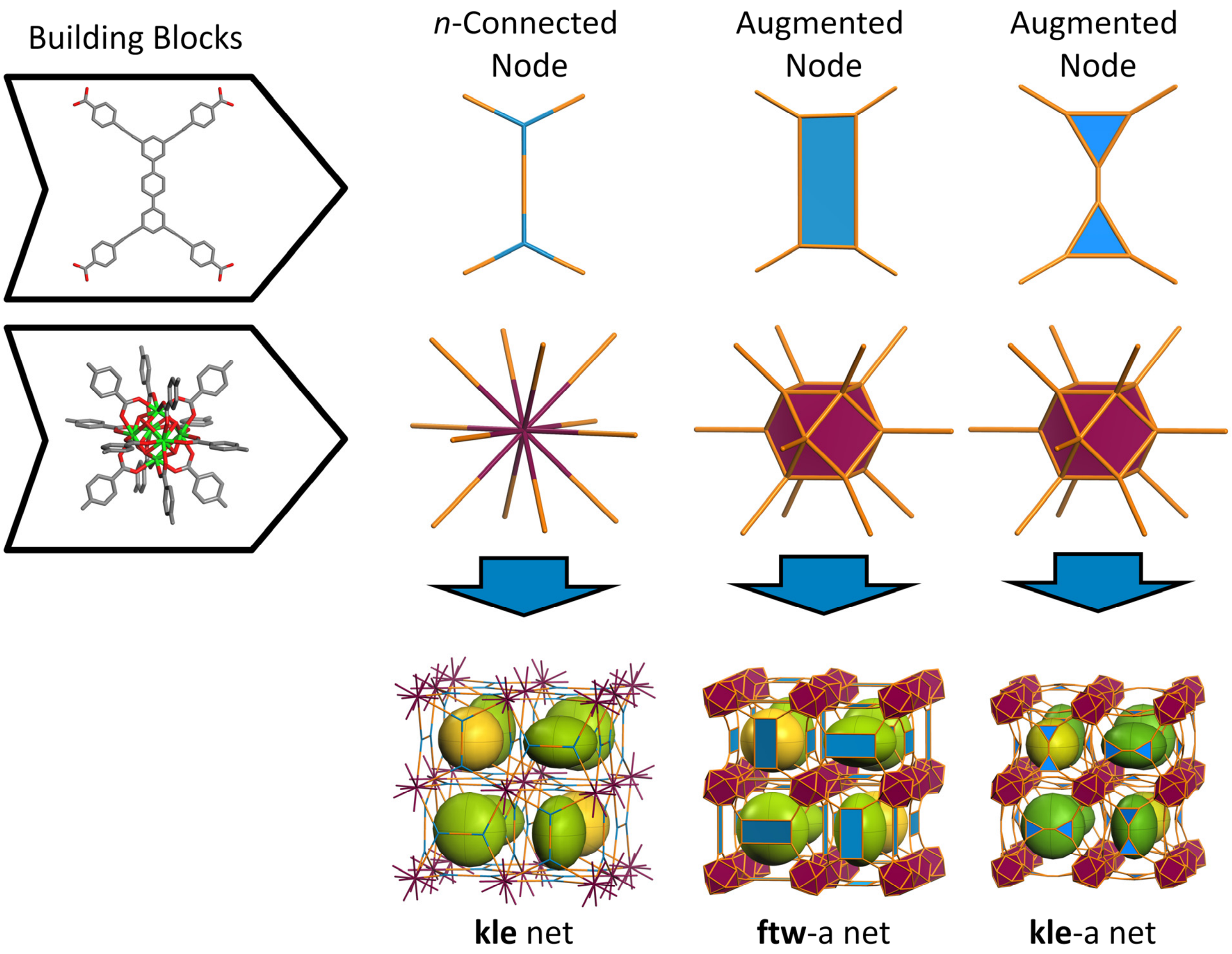

Figure S3: Molecular Building Blocks (MBBs) and geometric representation of **ftw**-MOF-3 resulting in **kle** and **ftw**-a nets.

Topological analyses were performed using TOPOS.[1]

ftw-**MOF-1:**

n-connected nodes: ftw

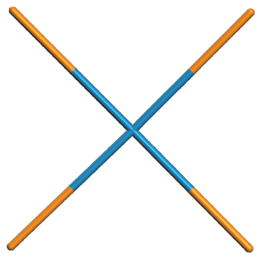 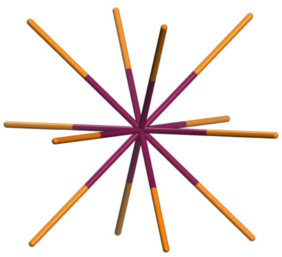

Coordination sequences:

**C1:**	**1**	**2**	**3**	**4**	**5**	**6**	**7**	**8**	**9**	**10**
**Number:**	4	32	36	178	108	442	220	826	372	1330
**Cumulative:**	5	37	73	251	359	801	1021	1847	2219	3549
**C1:**	**1**	**2**	**3**	**4**	**5**	**6**	**7**	**8**	**9**	**10**
**Number:**	12	18	108	74	324	170	660	306	1116	482
**Cumulative:**	13	31	139	213	537	707	1367	1673	2789	3271

TD10=3479

Point symbol for net: {4^36.6^30}{4^4.6^2}3

4,12-c net with stoichiometry (4-c)3(12-c); 2-nodal net

augmented nodes: ftw-a

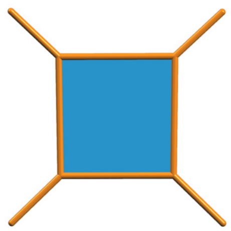 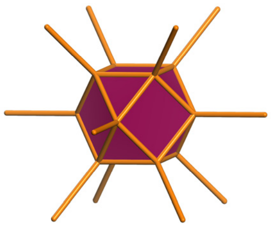

Coordination sequences:

**C1:**	**1**	**2**	**3**	**4**	**5**	**6**	**7**	**8**	**9**	**10**
**Number:**	5	12	18	30	48	75	118	143	160	208
**Cumulative:**	6	18	36	66	114	189	307	450	610	818
**C1:**	**1**	**2**	**3**	**4**	**5**	**6**	**7**	**8**	**9**	**10**
**Number:**	3	7	19	35	47	63	90	131	189	233
**Cumulative:**	4	11	30	65	112	175	265	396	585	818

TD10=818

Point symbol for net: { 3^2.4^2.5^2.8^4 }{ 4.8^2 }

3,5-c net with stoichiometry (3-c)(5-c); 2-nodal net

ftw-**MOF-2,** ftw-**MOF-2 (Naphth),** ftw-**MOF-2 (Anth) and** ftw-**MOF-3:**

Augmented nodes: ftw-a

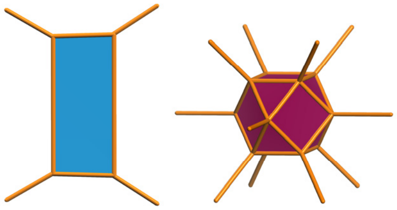

Coordination sequences:

**C1:**	**1**	**2**	**3**	**4**	**5**	**6**	**7**	**8**	**9**	**10**
**Number:**	5	12	18	30	48	75	118	143	160	208
**Cumulative:**	6	18	36	66	114	189	307	450	610	818
**C1:**	**1**	**2**	**3**	**4**	**5**	**6**	**7**	**8**	**9**	**10**
**Number:**	3	7	19	35	47	63	90	131	189	233
**Cumulative:**	4	11	30	65	112	175	265	396	585	818

TD10=818

Point symbol for net: {3^2.4^2.5^2.8^4}{4.8^2}
3,5-c net with stoichiometry (3-c)(5-c); 2-nodal net

***n*-connected nodes: New Topology (kle) (3,3,12-c Trinodal)**

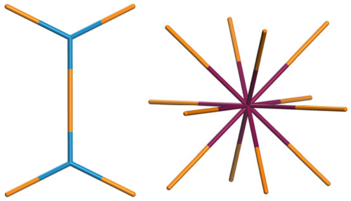

Coordination sequences:

**C1:**	**1**	**2**	**3**	**4**	**5**	**6**	**7**	**8**	**9**	**10**
**Number:**	12	18	60	114	140	234	348	378	524	714
**Cumulative:**	12	31	91	205	345	579	927	1305	1829	2543
**C2:**	**1**	**2**	**3**	**4**	**5**	**6**	**7**	**8**	**9**	**10**
**Number:**	3	23	40	88	159	211	293	420	500	612
**Cumulative:**	4	27	67	155	314	525	818	1238	1738	2350
**C3:**	**1**	**2**	**3**	**4**	**5**	**6**	**7**	**8**	**9**	**10**
**Number:**	3	23	40	88	159	211	293	420	500	612
**Cumulative:**	4	27	67	155	314	525	818	1238	1738	2350

TD10=2377

Point symbol for net: {4.5^2}6{4^6.5^24.6^6.7^18.8^12}
3,12-c net with stoichiometry (3-c)6(12-c); 2-nodal net
(NOTE: Using Systre, the correct designation of 3-nodal net is calculated)

X-ray Crystallography and Structural Analysis:

Powder X-ray Diffraction

Powder X-ray Diffraction (PXRD) measurements were collected at room temperature on a PANalytical X′Pert PRO MPD X-ray diffractometer at 45 kV, 40 mA for Cu Kα(λ = 1.5418 Å) with a scan speed of 1° min^{-1} and a step size of 0.02° in 2ϴ. A Whole Profile Pattern Matching was performed using the Le Bail methodology in order to verify the purity of each material.

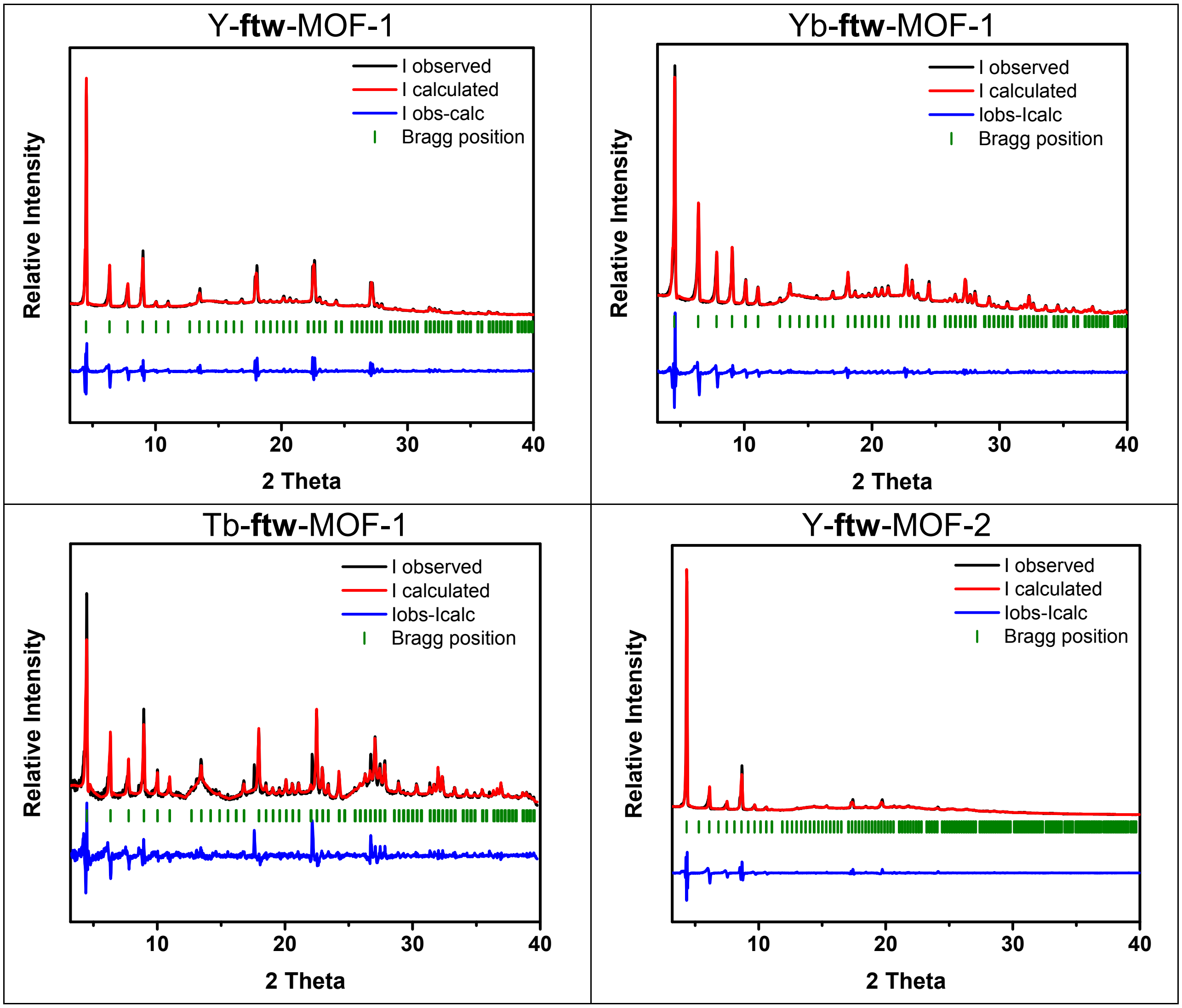

Figure S4a. (a) Le Bail refinement of Y, Yb, Tb-**ftw**-MOF-1 and Y- **ftw**-MOF-2.

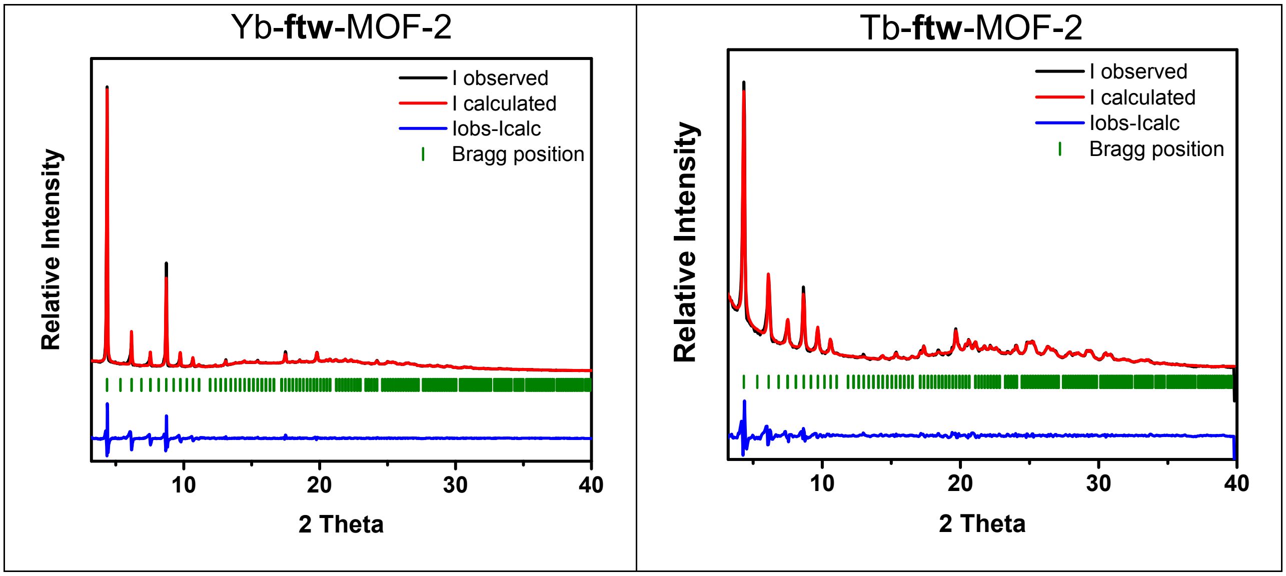

Figure S4b. (a) Le Bail refinement of Yb, Tb-**ftw**-MOF-2.

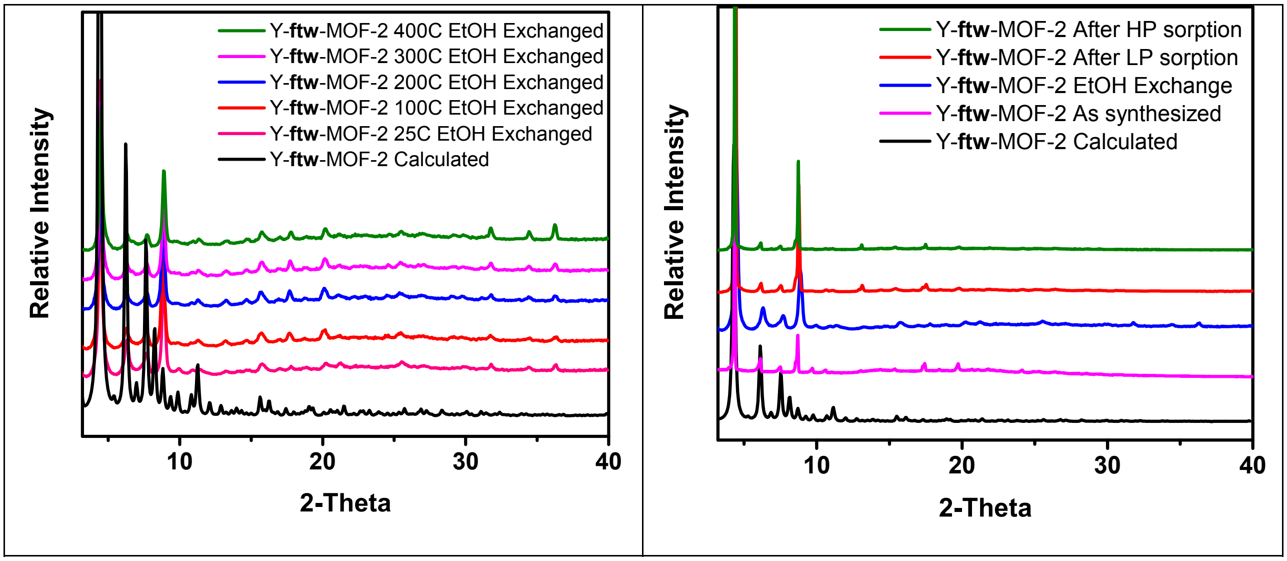

Figure S5. (left) Variable temperature PXRD of EtOH exchanged Y-**ftw**-MOF-2. (right) PXRD comparison of Y-**ftw**-MOF-2, EtOH exchanged and samples after sorption.

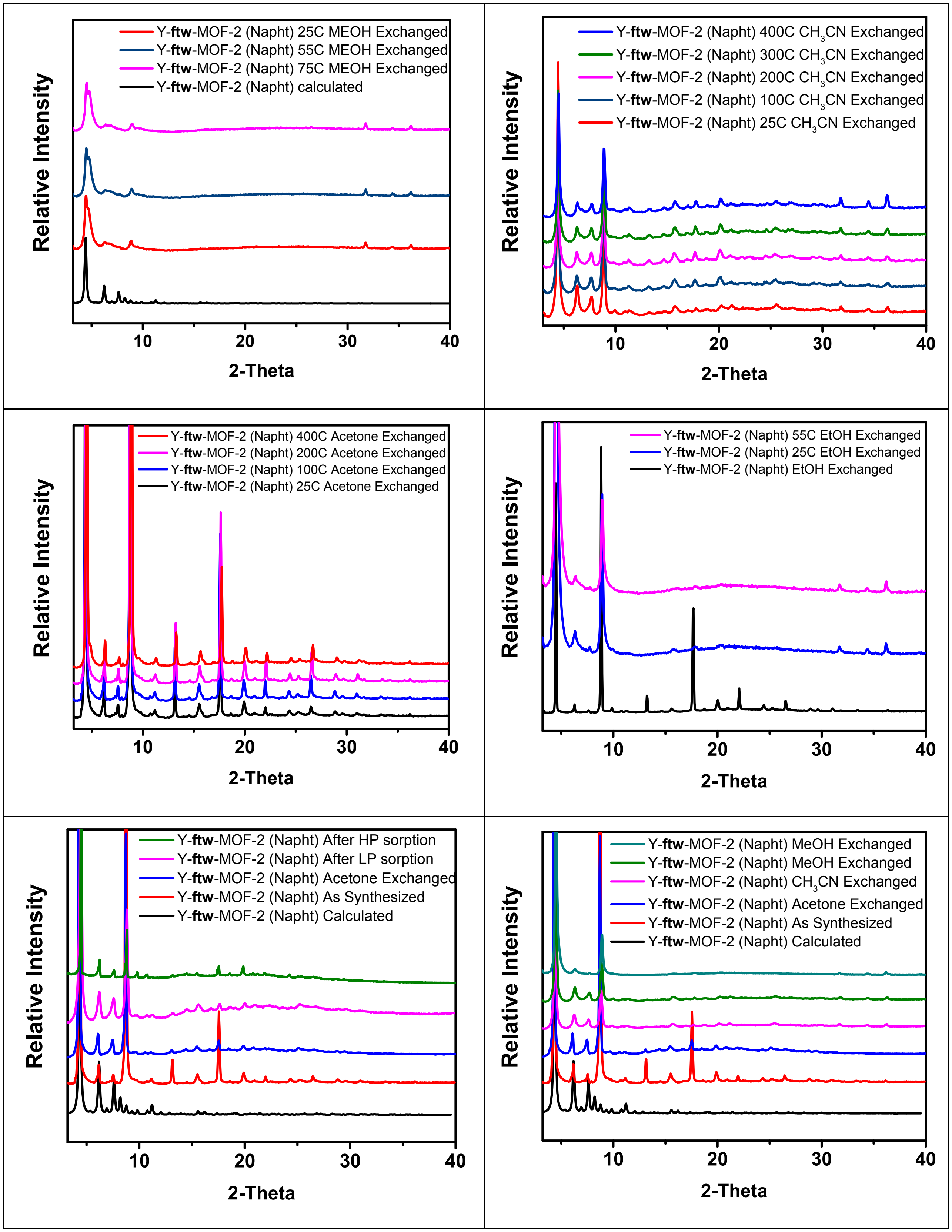

Figure S6. (a) Variable temperature PXRD of MeOH exchanged Y-**ftw**-MOF-2 (Naphth). (b) Variable temperature PXRD of CH_3CN exchanged Y-**ftw**-MOF-2 (Naphth). (c) Variable temperature PXRD of Acetone exchanged Y-**ftw**-MOF-2 (Naphth). (d) Variable temperature PXRD of EtOH exchanged Y- **ftw**-MOF-2 (Naphth). (e) PXRD comparison of Y-**ftw**-MOF-2 (Naphth), As Synthesized, Acetone exchanged and samples after sorption. (f) Comparison of the solvent exchanged Y-**ftw**-MOF-2 (Naphth).

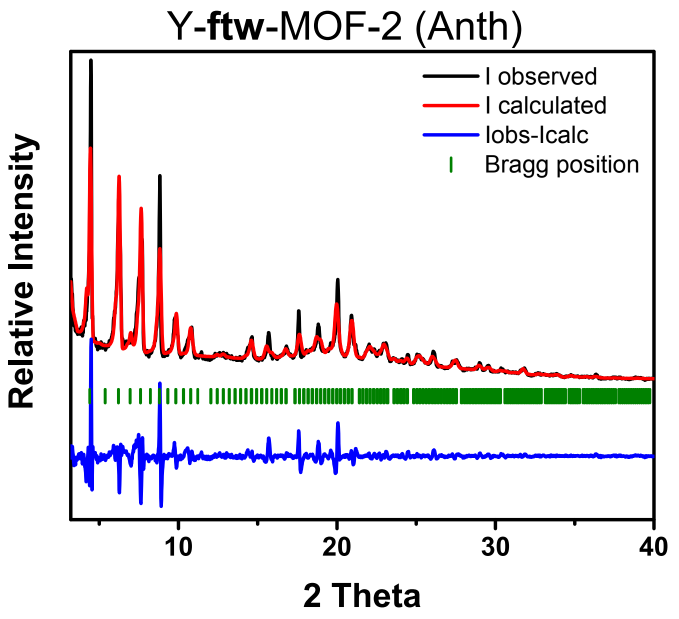

Figure S7. PXRD comparison of experimental and calculated Y-**ftw**-MOF-2 (Anth).

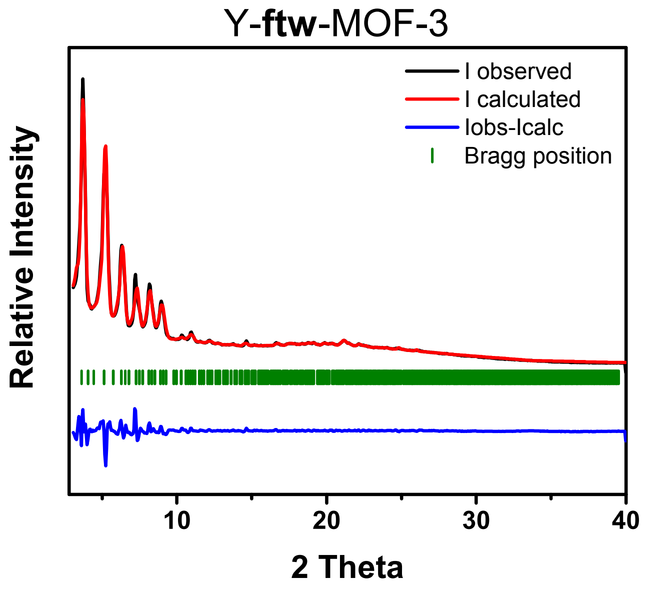

Figure S8. PXRD comparison of experimental and calculated Y-**ftw**-MOF-3.

Single Crystal X-ray Crystallography

The X-Ray diffraction data were collected using Bruker X8 PROSPECTOR APEX2 CCD diffractometer using CuKα (λ = 1.54178 Å). Indexing was performed using APEX2 (Difference Vectors method).[2] Data integration and reduction were performed using SaintPlus 6.01.[3] Absorption correction was performed by multi-scan method implemented in SADABS.[4] Space group was determined using XPREP implemented in APEX2.[2] Structure was solved using Direct Methods (SHELXS-97) and refined using SHELXL-97 (full-matrix least-squares on F2) contained in APEX2, WinGX v1.70.01[5,6] and OLEX2 programs packages.[7]

For all compounds the ligand moiety was disordered and atoms were refined using geometry restraints. Restraints were also used to refine anisotropic displacement parameters of disordered atoms. Disordered cations and solvent molecules were refined isotropically. Hydrogen atoms were placed in geometrically calculated positions and included in the refinement process using riding model with isotropic thermal parameters: Uiso (H) = 1.2 Ueq(−OH,−CH). Distance restraints have been used to refine disordered benzene rings. Disordered atoms have been refined isotropically. For the compounds the contribution of heavily disordered solvent molecules was treated as diffuse using Squeeze procedure implemented in Platon program.[8] Crystal data and refinement conditions are shown in Table S1 (Supporting Information).

Additionally, crystal of Y-**ftw**-MOF-2 was initially refined as merohedral twin with (0 1 0 1 0 0 0 0 -1 twinning law, BASF~0.5) and subsequently the data was detwinned using Shelxl-2013. Detwinned data have been treated with Squeeze procedure to account for disordered solvent.

Additionally, crystal of Y-**ftw**-MOF-3 was initially refined as merohedral twin with (0 1 0 1 0 0 0 0 -1 twinning law) and subsequently the data was detwinned using Shelxl-2013. Detwinned data have been treated with Squeeze procedure to account for disordered solvent.

**Table S1:** Crystal data and structure refinement for **ftw**-MOFs			
Identification code	Y-**ftw**-MOF-1	Y-**ftw**-MOF-2	Y-**ftw**-MOF-3
Empirical formula	$C_{114}H_{86}O_{52}Y_6$	$C_{138}H_{78}O_{35}Y_6$	$C_{162}H_{78}O_{32}Y_6$
Formula weight	2821.29	2829.46	3069.70
Temperature/K	100(2)	296.15	284(2)
Crystal system	Cubic	Cubic	Cubic
Space group	Pm-3m	Im-3	Im-3
a/Å	19.3042(5)	40.048(3)	48.111(16)
b/Å	19.3042(5)	40.048(3)	48.111(16)
c/Å	19.3042(5)	40.048(3)	48.111(16)
α/°	90	90	90
β/°	90	90	90
γ/°	90	90	90
Volume/Å^3	7193.8(3)	64229(13)	111362(112)
Z	1	8	8
ρ_{calc}mg/mm^3	0.651	0.585	0.366
m/mm^{-1}	1.889	1.655	0.964
F(000)	1420.0	11360.0	12320.0
Crystal size/mm^3	0.08 × 0.06 × 0.05	0.01 × 0.01 × 0.01	0.05 × 0.04 × 0.03
2Θ range for data collection	10.24 to 131.92°	7.646 to 88.922	2.596 to 72.688
Index ranges	-22 ≤ h ≤ 21, -22 ≤ k ≤ 17, -21 ≤ l ≤ 21	-28 ≤ h ≤ 29, -39 ≤ k ≤ 15, -38 ≤ l ≤ 16	-29 ≤ h ≤ 33, -36 ≤ k ≤ 33, -32 ≤ l ≤ 36
Reflections collected	21081	4464	4688
Independent reflections	1288[R(int) = 0.0577]	4464 [R_{int} = ?, R_{sigma} = 0.0803] Rint is missing due to the fact, that detwinned data have been imported from FCF file (containing merged data) generated by SHelxl-2013 with LIST 8 instruction. R(int) = 0.1045 for data refined with TWIN law before detwinning procedure has been applied	4688 [R_{int} = ?, R_{sigma} = 0.0592]. Rint is missing due to the fact, that detwinned data have been imported from FCF file (containing merged data) generated by SHelxl-2013 with LIST 8 instruction.
Data/restraints/parameters	1288/63/86	4464/346/280	4688/764/493
Goodness-of-fit on F^2	1.189	1.174	0.871
Final R indexes [I>=2σ (I)]	R_1 = 0.0834, wR_2 = 0.2599	R_1 = 0.0983, wR_2 = 0.2380	R_1 = 0.0779, wR_2 = 0.2006
Final R indexes [all data]	R_1 = 0.0922, wR_2 = 0.2626	R_1 = 0.1233, wR_2 = 0.2493	R_1 = 0.1040, wR_2 = 0.2196
Largest diff. peak/hole / e Å^{-3}	0.60/-0.88	2.27/-0.83	1.01/-0.63

Structure Analysis and Ligand Geometry:

Geometric and Molecular Representation of Cages:

Diagrams of the cages in **ftw**-MOF-2:

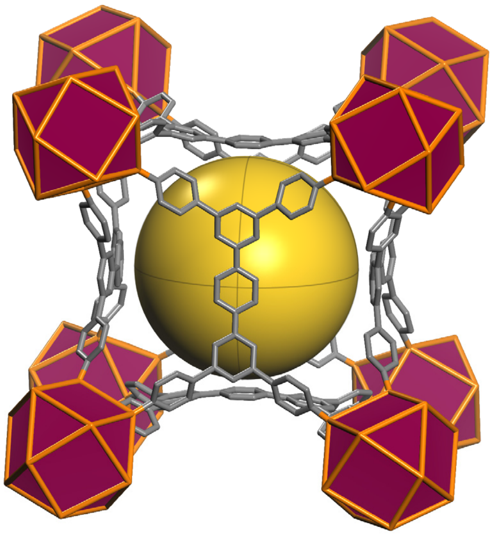

Figure S9: Symmetric cage in **ftw**-MOF-2. Hexanuclear MBBs are represented as purple polyhedra.

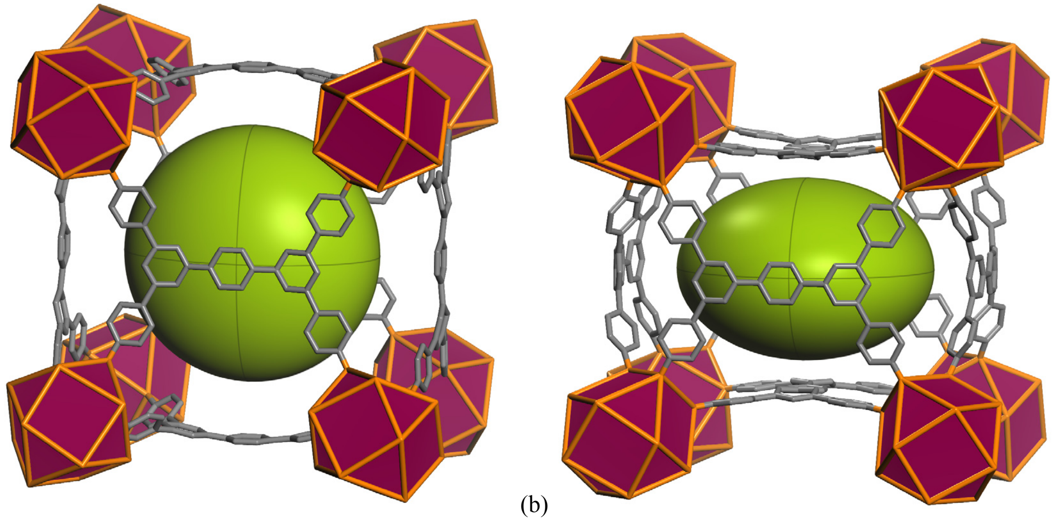

(a) (b)

Figure S10: Asymmetric cage in **ftw**-MOF-2. Hexanuclear MBBs are represented as purple polyhedra. (a) View 1 (along X axis) (b) View 2 (along Y or Z axis).

Diagrams of the cages in **ftw**-MOF-3:

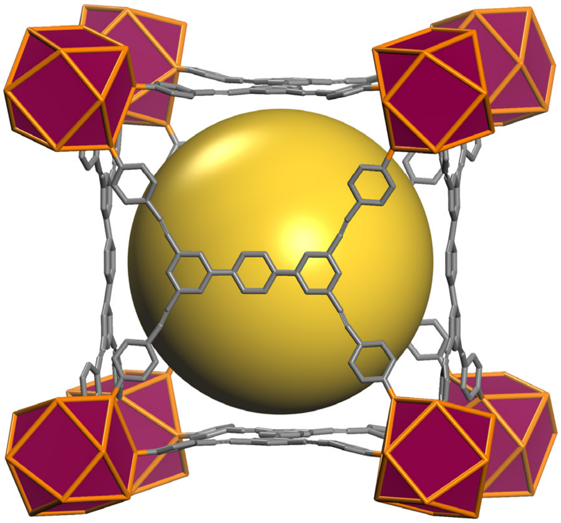

Figure S11: Symmetric cage in **ftw**-MOF-3. Hexanuclear MBBs are represented as purple polyhedra.

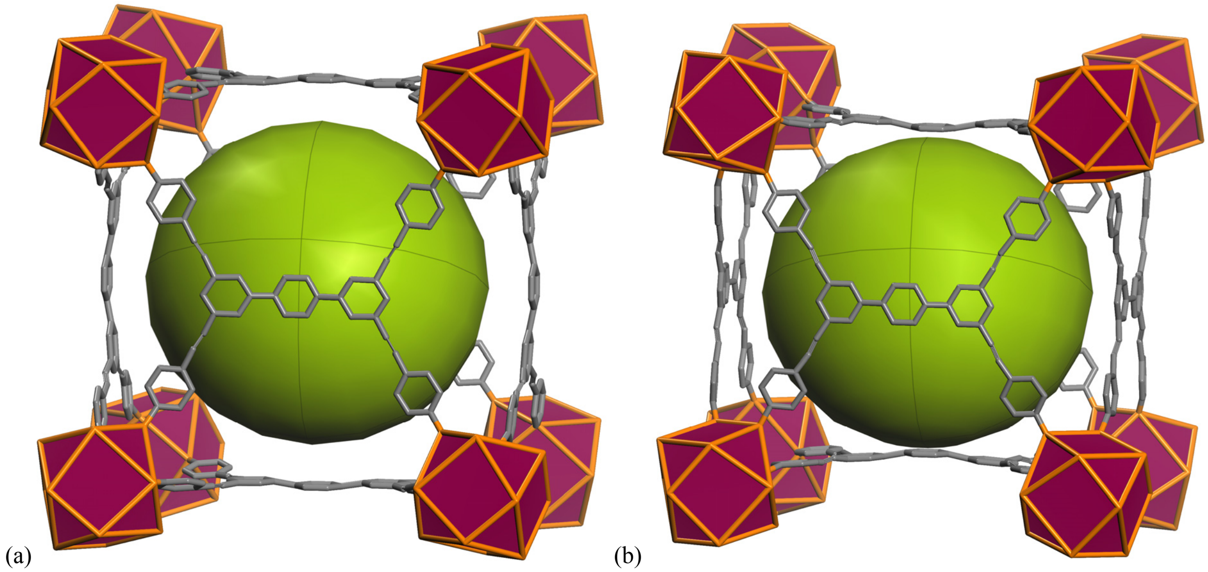

Figure S12: Asymmetric cage in **ftw**-MOF-3. Hexanuclear MBB are represented as purple polyhedra. (a) View 1 (along x-axis) (b) View 2 (along Y or Z axis).

Diagrams of the cages in **ftw**-MOF-2 (Naphth):

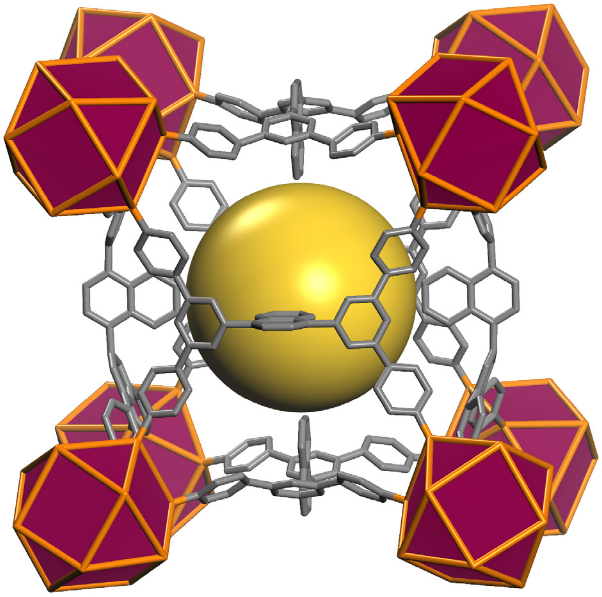

Figure S13: Symmetric cage in **ftw**-MOF-2 (Naphth). Hexanuclear MBBs are represented as purple polyhedra.

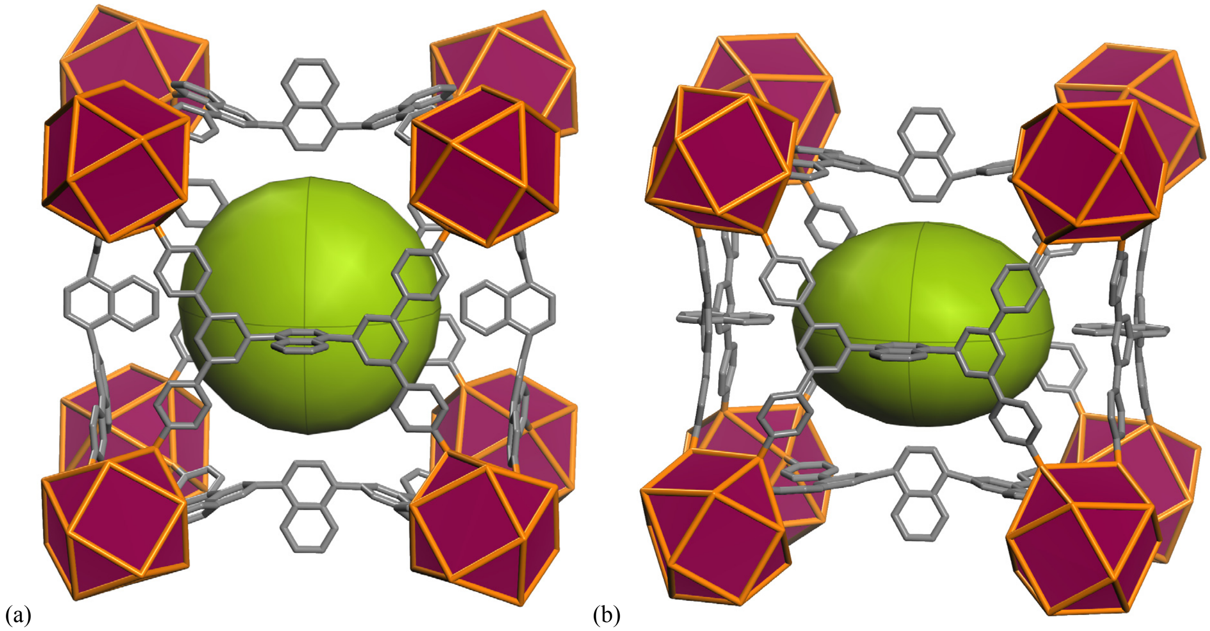

Figure S14: Asymmetric cage in **ftw**-MOF-2 (Naphth). Hexanuclear MBBs are represented as purple polyhedra. (a) View 1 (along X axis) (b) View 2 (along Y or Z axis).

Diagrams of the modelled cages in **ftw**-MOF-2 (Anth):

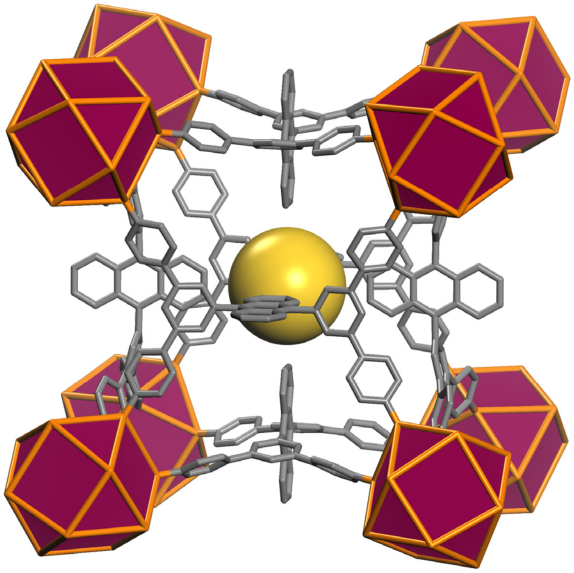

Figure S15: Symmetric cage in **ftw**-MOF-2 (Anth). Hexanuclear MBBs are represented as purple polyhedra.

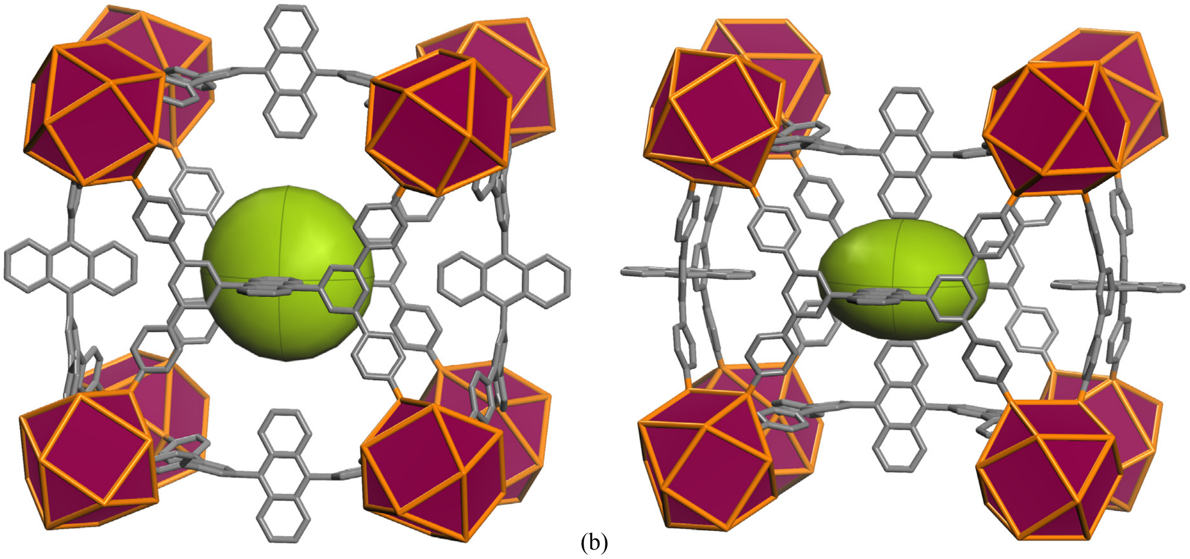

(a) (b)

Figure S16: Asymmetric cage in **ftw**-MOF-2 (Anth). Hexanuclear MBBs are represented as purple polyhedra. (a) View 1 (along X axis) (b) View 2 (along Y or Z axis).

Ligand Geometry Analysis:

The dimensions and aspect ratio of geometry optimized ligands as well as the crystallographically determined geometry is shown in the table below. This was performed on the ligands based on their crystallographic data as well as a geometry optimized ligand. Geometry optimization was performed using the geometry optimization option in the Forcite module of Accelrys Materials Studio V 6.1. The dimensions were determined using Accelrys Materials Studio V 6.1 by measuring the distance between the carbon atoms of the carboxylates. The aspect ratio is the width to height ratio based on these measurements.

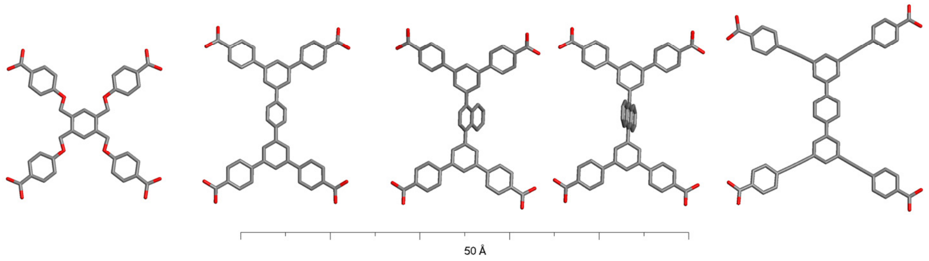

	**TetPOMB**	**TCPT**	**TCDPN**	**TCDPA**	**TCEPT**
**Geometry Optimized Width (Å)**	13.026	12.491	12.481	12.486	16.994
**Geometry Optimized Length (Å)**	13.523	15.943	15.733	15.791	18.901
**Geometry Optimized Aspect Ratio**	0.96:1	0.78:1	0.79:1	0.79:1	0.90:1
**Crystallographically Determined Width (Å)**	12.738	12.311	12.309	N/A	16.462
**Crystallographically Determined Length (Å)**	12.738	15.531	N/A	N/A	18.702
**Crystallographically Determined Aspect Ratio**	1:1	0.79:1	N/A	N/A	0.88:1

Table S2: Crystallographically determined and geometry optimized ligand dimensions and aspect ratios.

Thermal Gravimetric Analysis:

Thermal Gravimetric Analysis (TGA) was performed on a TA Instruments hi-res TGA Q5000IR with High Resolution TGA (Hi-Res TGA) capability. Experiments were performed under an N_2 atmosphere with balance and sample purge flow rates of 10ml min^{-1} and 25ml min^{-1}, respectively. Samples were placed in 100µl high temperature platinum crucibles and heated in Hi-Res TGA mode with a heating rate of 5°C min^{-1} and a resolution index of 3 and a sensitivity index of 5.

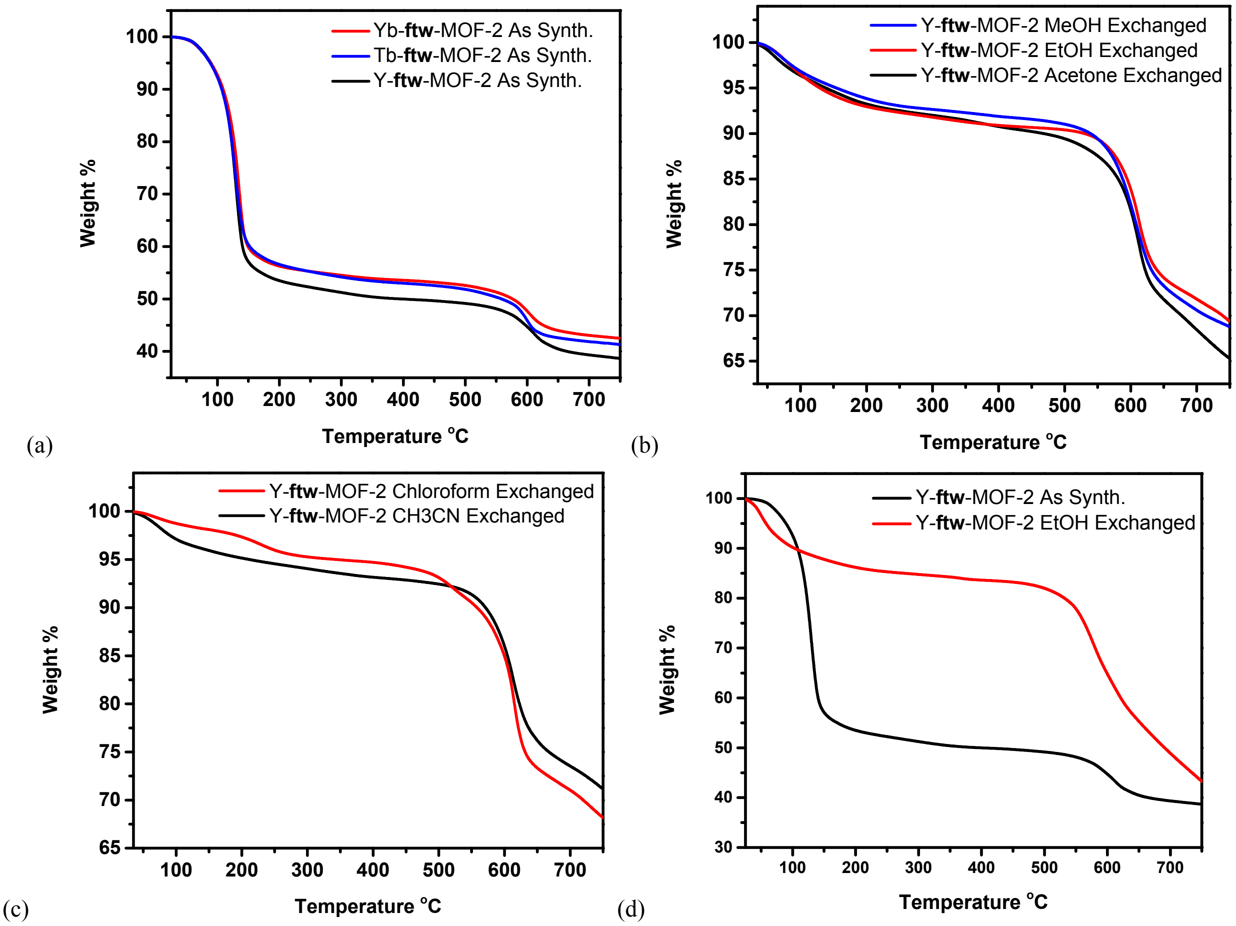

Figure S17. (a) TGA of the DMF washed Y, Tb, and Yb **ftw**-MOF-2 variants. (b) TGA of the MeOH, EtOH and Acetone Exchanged Y-**ftw**-MOF-2. (c) TGA of the Chloroform and CH_3CN Exchanged Y-**ftw**-MOF-2. (d) TGA of the DMF washed and EtOH Exchanged Y-**ftw**-MOF-2.

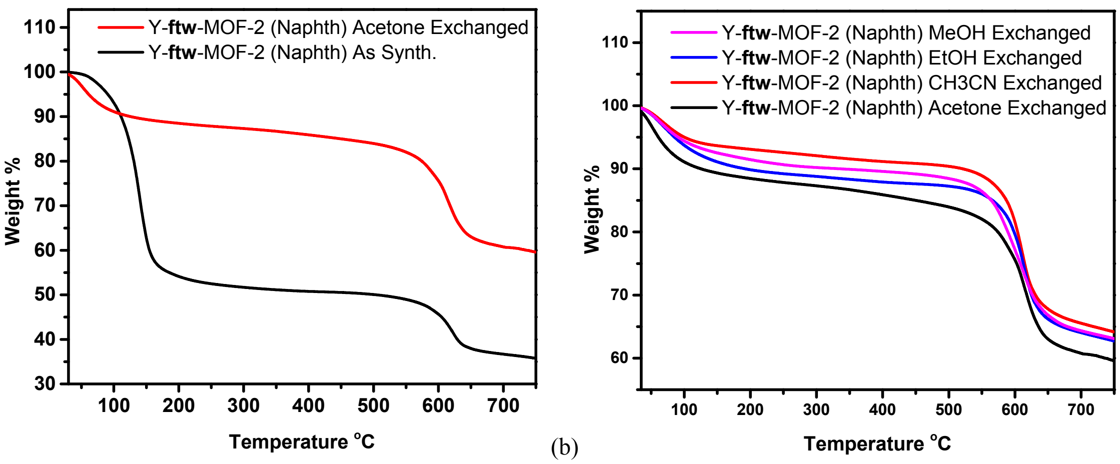

(a) (b)

Figure S18. (a) TGA of the DMF washed Y-**ftw**-MOF-2 (Naphth). (b) TGA comparison of the solvent exchanged Y-**ftw**-MOF-2 (Naphth) samples.

Gas Sorption Analysis:

Low Pressure Gas Adsorption Measurements:

High resolution low pressure gas adsorption measurements were performed on a fully automated Autosorb-1C gas sorption analyzer (Quantachrome Instruments) at relative pressures up to 1 atm. The cryogenic temperature was controlled using liquid nitrogen and argon baths at 77 K and 87 K, respectively. The bath temperature for collecting the CO_2 sorption isotherms (258-278K) was controlled using a circulating bath containing an ethylene glycol/water mixture. The apparent surface areas were determined from the argon adsorption isotherms collected at 87 K by applying the Brunauer-Emmett-Teller (BET) and Langmuir models. Pore size analyses were performed using a cylindrical/spherical NLDFT pore model system by assuming an oxidic (zeolitic) surface. The determination of the isosteric heats of adsorption (Qst) for H_2 and CO_2 was estimated by applying the Clausius-Clapeyron expression using the H_2 sorption isotherms measured at 77 K and 87 K and the CO_2 isotherms measured at 258, 273 and 298 K. Homogenous microcrystalline samples of Y-**ftw**-MOF-2 were activated by washing the as-synthesized crystals with 3 x 20 ml DMF over a period of 3 days. This was followed by solvent exchange in ethanol for 3 days. The solution was refreshed several times daily during this time period. Homogenous microcrystalline samples of Y-**ftw**-MOF-2 (Naphth) were activated by washing the as-synthesized crystals with 3 x 20 ml DMF over a period of 3 days. This was followed by solvent exchange in acetone for 3 days. The solution was refreshed several times daily during this time period. Homogenous microcrystalline samples of Y-**ftw-**MOF-2 (Anth)were activated by washing the as-synthesized crystals with 3 x 20 ml DMF over a period of 3 days. This was followed by solvent exchange in methanol or ethanol for 3 days. The solution was refreshed several times daily during this time period. In a typical experiment, 30 to 40 mg of each exchanged sample was transferred to a 6-mm large bulb glass sample cell and the sample is dried under a flow of N_2 overnight. The samples were then evacuated at room temperature using a turbo molecular vacuum pump and then gradually heated to 433 K (Y-**ftw**-MOF-2), 413 K (Y-**ftw**-MOF-2(Naphth)), and 378 K (Y-**ftw**-MOF-2(Anth)) (increasing at a rate of 1°C/min), held for 16 h and cooled to room temperature.

Compound	**BET (m^2g^{-1})[a]**	**Langmuir (m^2g^{-1})[a]**	**Calculated Density (g cc^{-1})[b]**	**% Free Volume [b]**	**Experimental P. V.(cm^3g^{-1})[a]**	**Calculated P.V. (cm^3g^{-1})[c]**	**Reference**
Y-**ftw**-MOF-2	3790	3730	0.575	72%	1.26	1.26	This Work
Y-**ftw**-MOF-2 (Naphth)	3040	3100	0.615	68%	1.05	1.11	This Work
Y-**ftw**-MOF-2 (Anth)	2100	2500	0.646	66%	0.79	1.03	This Work
Y-**ftw**-MOF-3	n/a	n/a	0.366	81%	n/a	2.41	This Work
Tb UTSA-61a	770	1510,1900	0.474	79%	0.67	1.66	Ref[9]
Y-FTZBP	2410	n/a	0.638	66%	0.94	1.03	Ref[10]

Table S3: Porosity summary for reported **ftw**-MOFs and select rare earth MOFs.

[a] Based on Ar Isotherms collected at 87K.

[b] Calculated from crystal structure in Materials Studio. Counter-ions and solvents omitted.
[c] Potential solvent accessible volumes were calculated using Accelrys Materials Studio, “Atom Volumes and Surfaces”

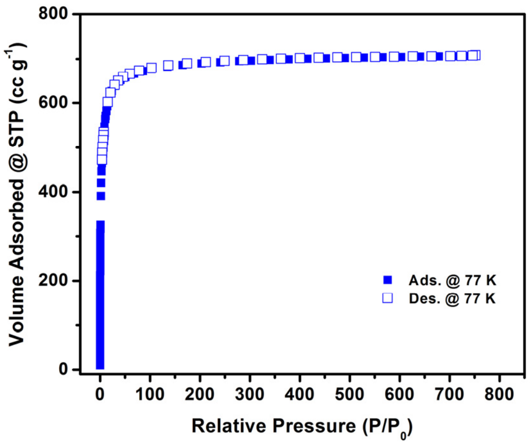

Figure S19. (a) N_2 adsorption isotherms at 77 K on Y-**ftw**-MOF-2.

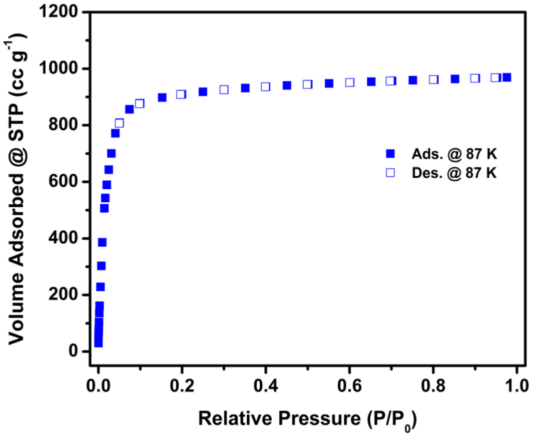

(a)

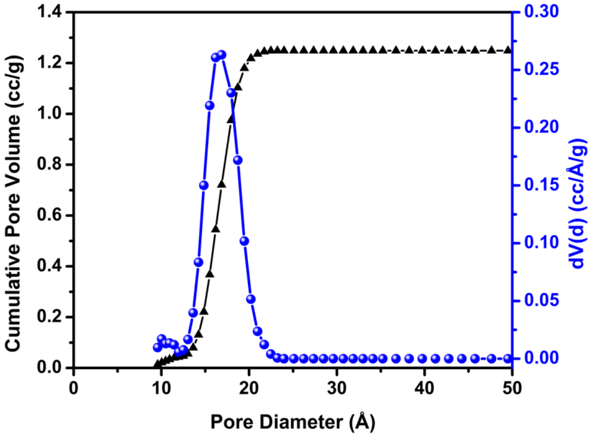

(b)

Figure S20. (a) Ar adsorption isotherms at 87 K on Y-**ftw**-MOF-2. (b) Pore size distribution for Y-**ftw**-MOF-2 determined from Ar adsorption at 87 K.

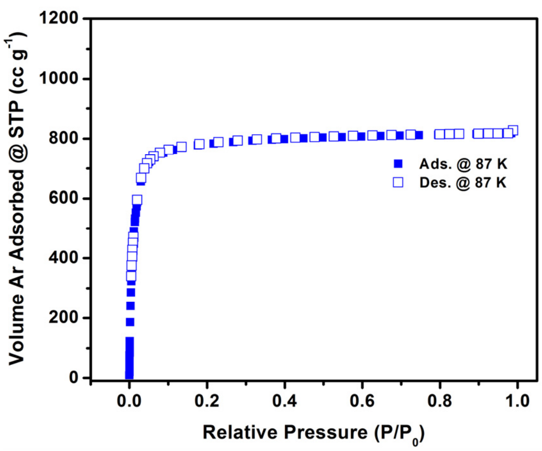

(a)

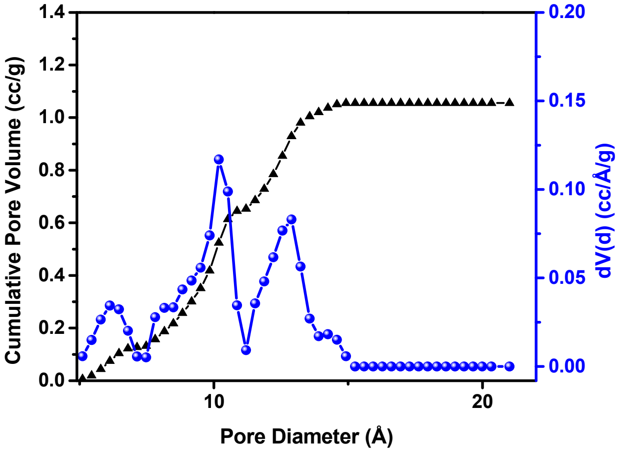

(b)

Figure S21. (a) Ar adsorption isotherms at 87 K on Y-**ftw**-MOF-2 (Naphth). (b) Pore size distribution for Y-**ftw**-MOF-2 (Naphth) determined from Ar adsorption at 87 K.

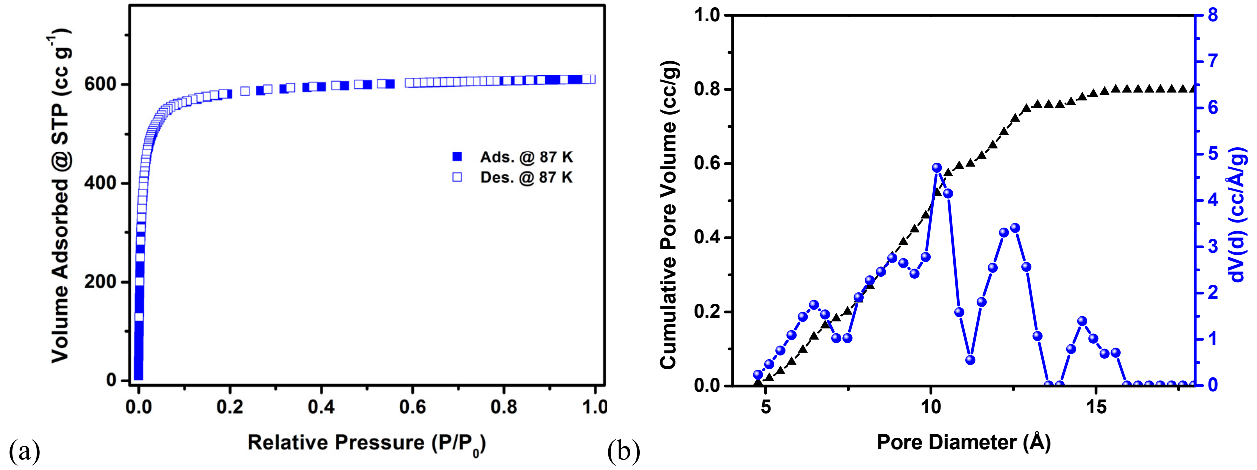

Figure S22. (a) Ar adsorption isotherms at 87 K on Y-**ftw**-MOF-2 (Anth). (b) Pore size distribution for Y-**ftw**-MOF-2 (Anth) determined from Ar adsorption at 87 K.

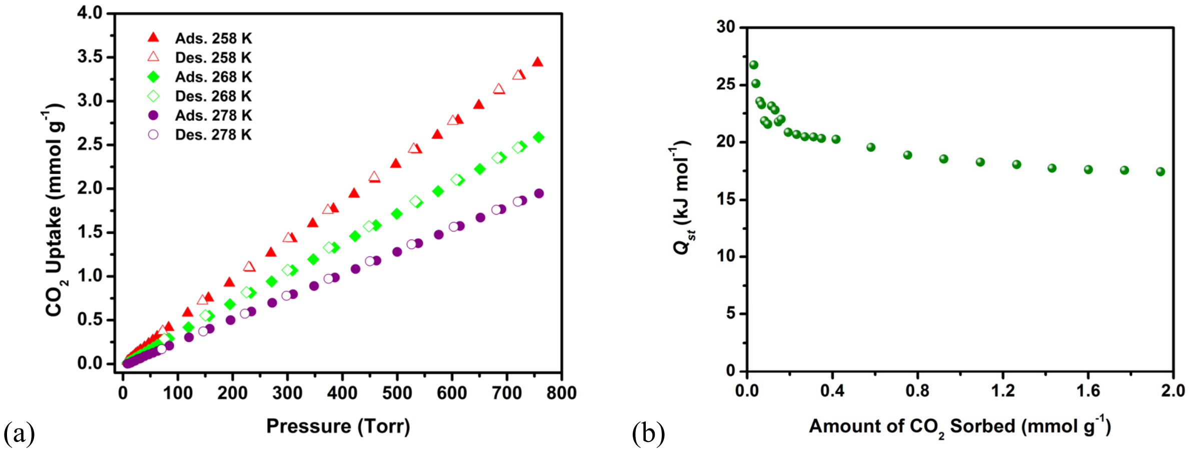

Figure S23. (a) Variable temperature CO_2 adsorption isotherms for Y-**ftw**-MOF-2. (b) *Qst* of CO_2 adsorption for Y-**ftw**-MOF-2.

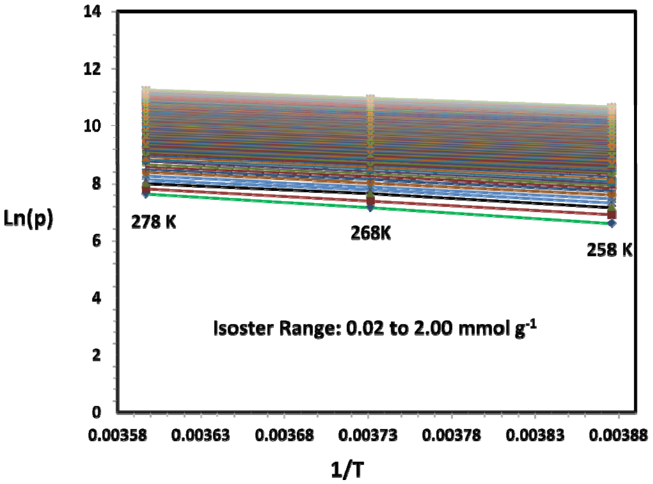

Figure S24. CO_2 isosters for Y-**ftw**-MOF-2.

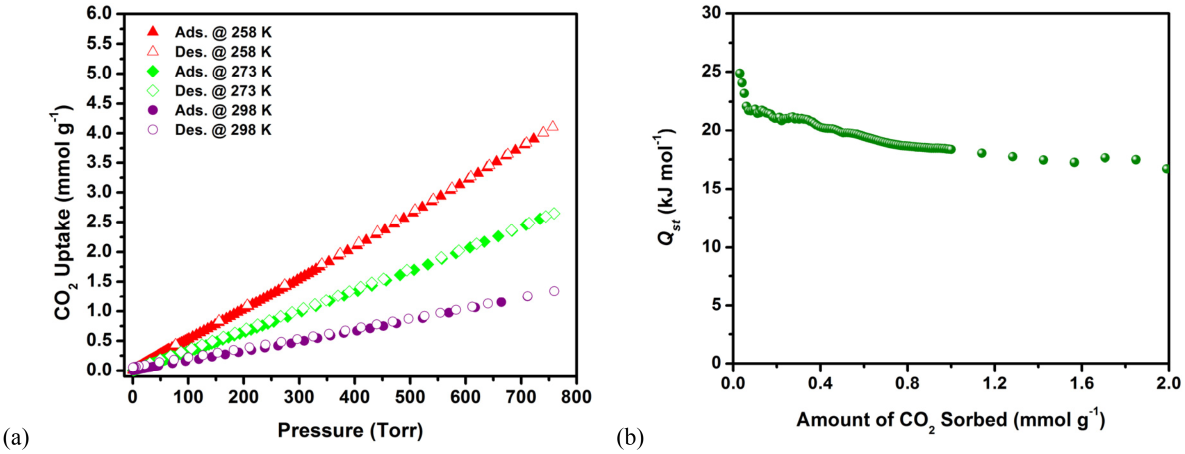

Figure S25. (a) Variable temperature CO_2 adsorption isotherms for Y-**ftw**-MOF-2 (Naphth). (b) *Qst* of CO_2 adsorption for Y Y-**ftw**-MOF-2 (Naphth).

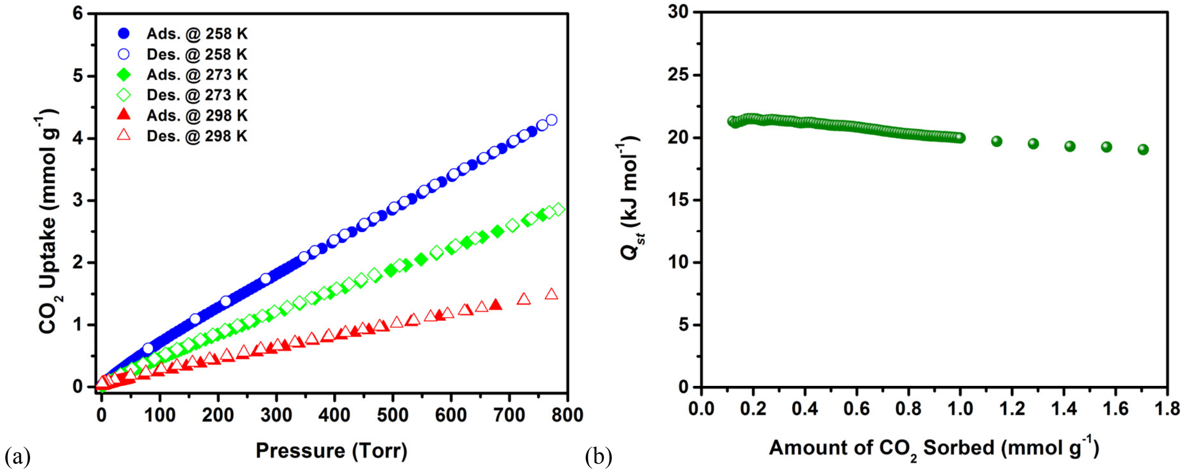

Figure S26. (a) Variable temperature CO_2 adsorption isotherms for Y-**ftw**-MOF-2 (Anth). (b) *Qst* of CO_2 adsorption for Y-**ftw**-MOF-2 (Anth).

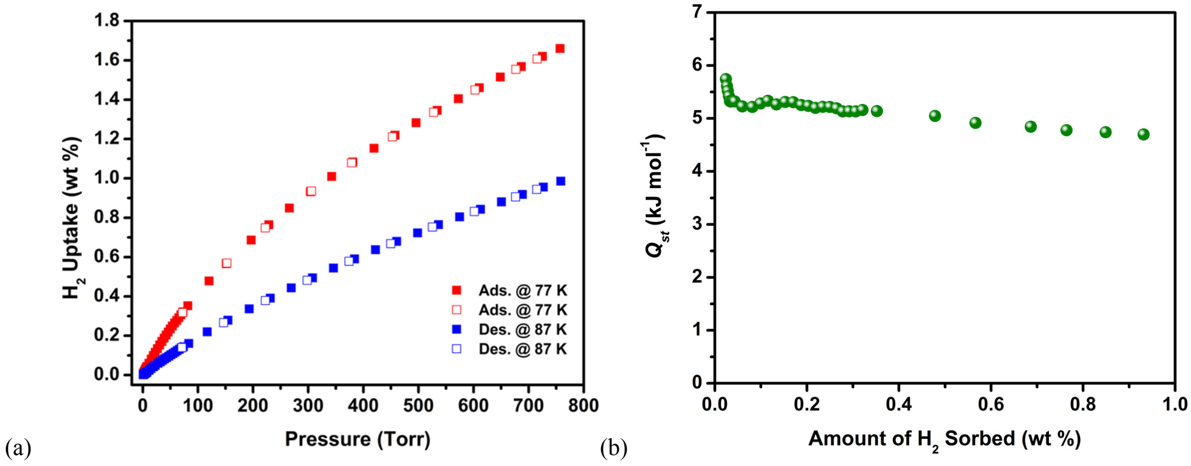

Figure S27. (a) H_2 adsorption isotherms for Y-**ftw**-MOF-2 at 87 and 77 K. (b) *Qst* for H_2 adsorption for Y-**ftw**-MOF-2.

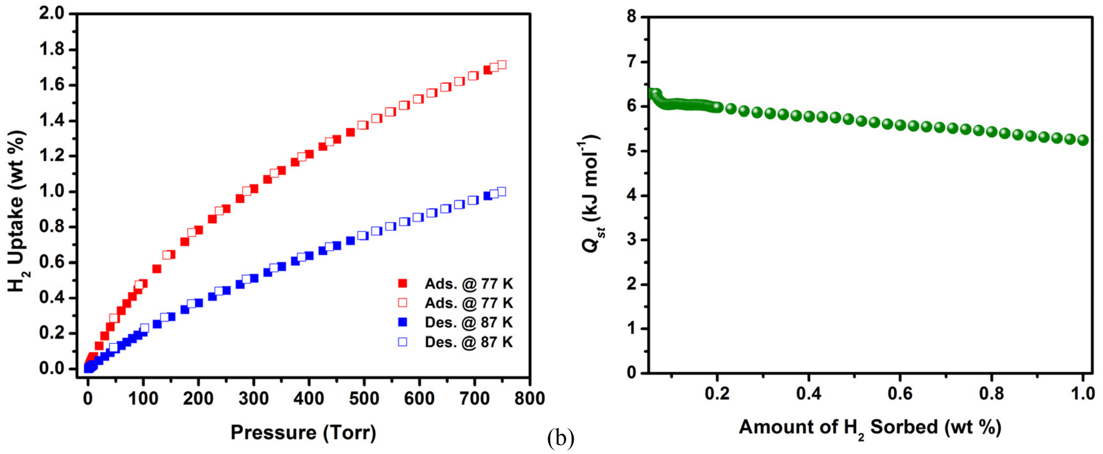

(a) (b)

Figure S28. (a) H_2 adsorption isotherms for Y-**ftw**-MOF-2 (Naphth) at 87 and 77 K. (b) *Qst* for H_2 adsorption for Y-**ftw**-MOF-2 (Naphth).

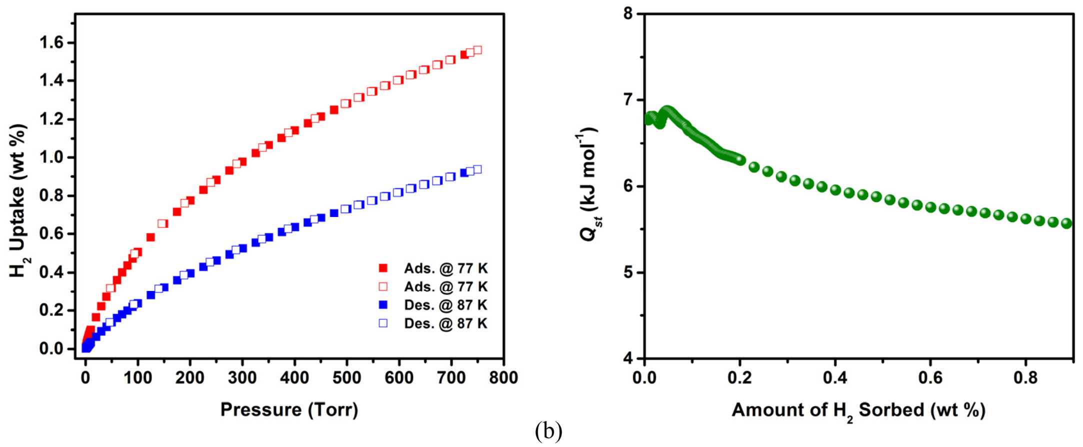

(a) (b)

Figure S29. (a) H_2 adsorption isotherms for Y-**ftw**-MOF-2 (Anth)at 87 and 77 K. (b) *Qst* for H_2 adsorption for Y-**ftw**-MOF-2 (Anth).

High-Pressure Gas Adsorption Measurements

Adsorption equilibrium measurements of single gases were performed using a Rubotherm gravimetric-densimetric apparatus (Bochum, Germany) (Scheme S1), composed mainly of a magnetic suspension balance (MSB) and a network of valves, mass flowmeters and temperature and pressure sensors. In a typical adsorption experiment, the adsorbent is precisely weighed and placed in a basket suspended by a permanent magnet through an electromagnet. The cell in which the basket is housed is then closed and vacuum or high pressure is applied.

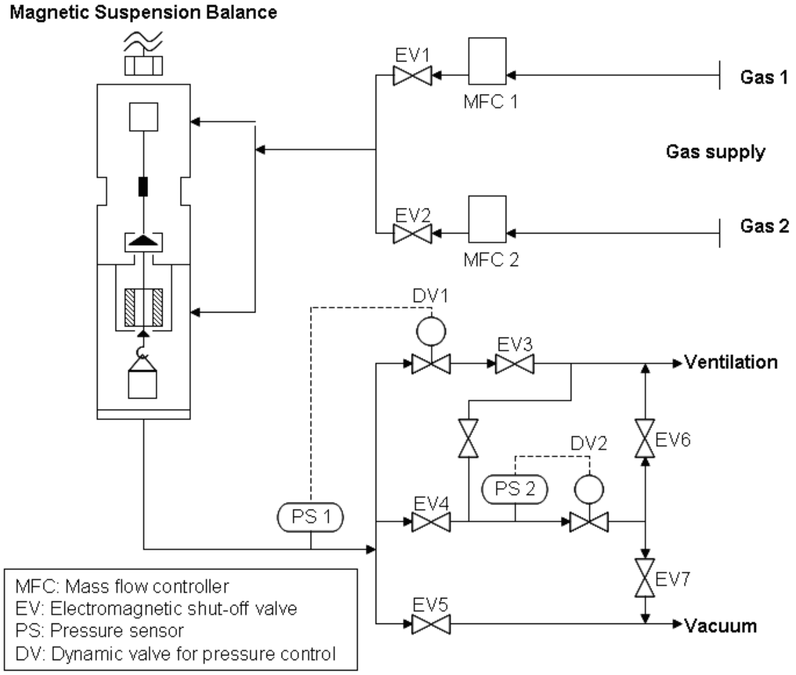

Scheme S1. Representation of the Rubotherm gravimetric-densimetric apparatus.

The evacuated (*in-situ*) adsorbent is then exposed to a continuous gas flow (typically 50 ml/min) or static mode at a constant temperature. The gravimetric method allows the direct measurement of the reduced gas adsorbed amount Ω. Correction for the buoyancy effect is required to determine the excess adsorbed amount using equation 1, where Vadsorbent and Vss refer to the volume of the adsorbent and the volume of the suspension system, respectively. These volumes are determined using the helium isotherm method by assuming that helium penetrates in all open pores of the materials without being adsorbed. The density of the gas is determined experimentally using a volume-calibrated titanium cylinder. By weighing this calibrated volume in the gas atmosphere, the local density of the gas is also determined. Simultaneous measurement of adsorption capacity and gas phase density as a function of pressure and temperature is therefore possible. The excess uptake is the only experimentally accessible quantity and there is no reliable experimental method to determine the absolute uptake. The absolute uptake was calculated assuming the adsorbed phase volume equal to the pore volume calculated using Ar adsorption at 87K.

$$\Omega = m_{excess} - \rho_{gas}(V_{adsorbent} + V_{ss}) \qquad (1)$$

The pressure is measured using two Drucks high pressure transmitters ranging from 0.5 to 34 bar and 1 to 200 bar, respectively, and one low pressure transmitter ranging from 0 to 1 bar. Prior to each adsorption experiment, about 100 mg to 300 mg sample is outgassed at 433 K at a residual pressure 10^{-6} mbar. The temperature during adsorption measurements is held constant by using a thermostated circulating fluid.

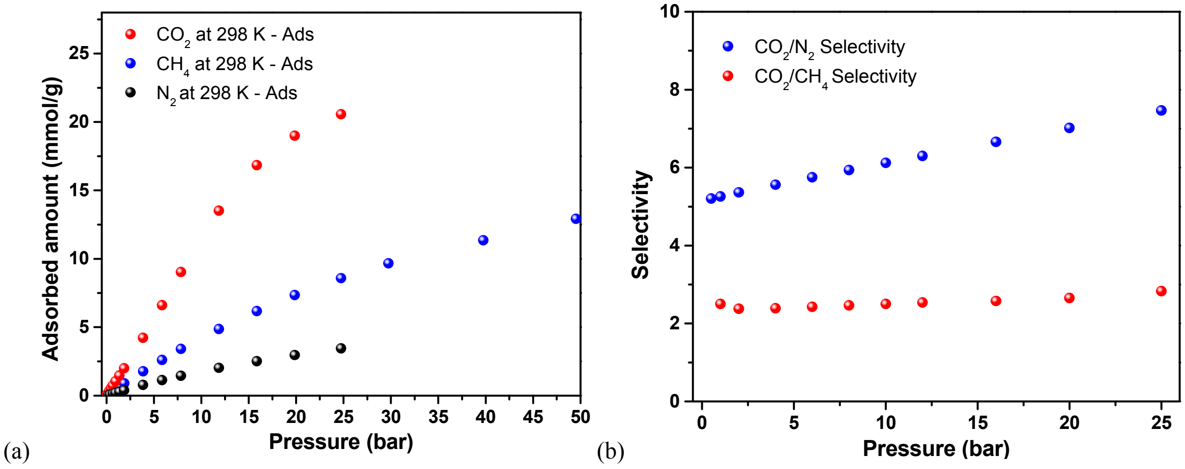

Figure S30. (a) Adsorption of CO_2, CH_4 and N_2 on Y-**ftw**-MOF-2. (b) CO_2/CH_4: 05/95 and CO_2/N_2: 10/90 on Y-**ftw**-MOF-2 determined using IAST.

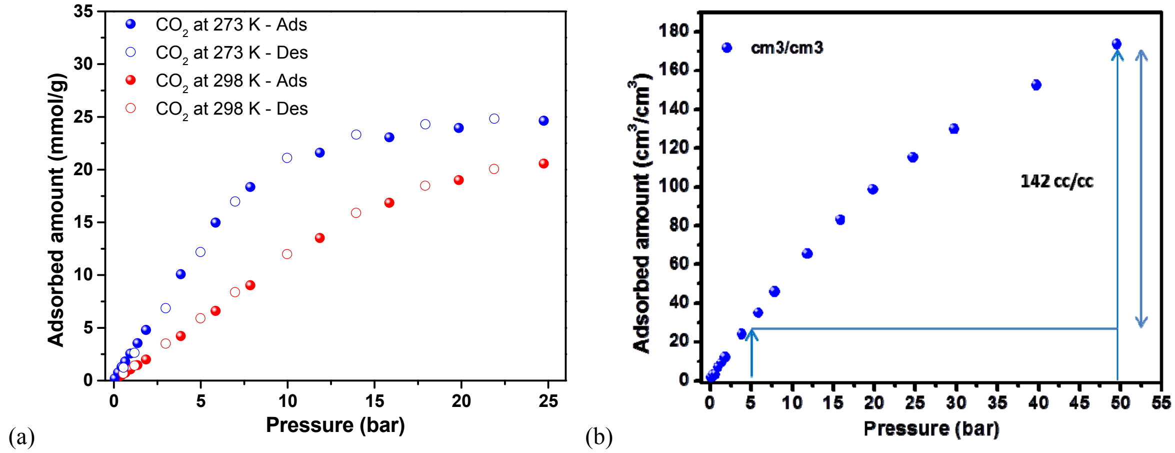

Figure S31. (a) Adsorption isotherm of CO_2 on Y-**ftw**-MOF-2 at 298 and 273 K. (b) Volumetric absolute CH_4 uptake for Y-**ftw**-MOF-2 at 298 K as a function of pressure.

Adsorbent	Estimated CH_4 adsorption (Total) uptake at 5 bar cm^3 (STP)/cm^3	Estimated CH_4 adsorption (Total)uptake at 50 bar cm^3 (STP)/cm^3	Working storage (Total) uptake (5 bar – 50 bar) cm^3 (STP)/cm^3	Total uptake at 35 bar cm^3 (STP)/cm^3	Working Capacity 5-35 bar	Reference
Y-**ftw**-MOF-2	32	174	142	145	113	This Work
**gea**-MOF-1	40	162	122	140	100	Ref[11]
**Y-pek**-MOF-1	40	175	135	150	110	Ref[12]
**Yb-pek**-MOF-2	43	166	123	140	97	Ref[12]
**UTSA-76a**	60	240	180	211	151	Ref[13]
**UTSA-20**	90	178*	80*	178	88	Ref[14]
**PCN-14**	90	220*	130*	220	130	Ref[15]
**NU-125**	50	215	165	180	130	Ref[15]
**NU-111**	30	175	145	140	110	Ref[15]
**HKUST-1**	90	242	152	225	135	Ref[15]
**MOF-519**	49	240	191	200	151	Ref[16]

Table S4. Volumetric CH_4 working capacity using adsorption and desorption at 5 bar and 50 bar, respectively for Y-**ftw**-MOF-2 and other relevant MOFs.

*Data only available to 35 Bar.

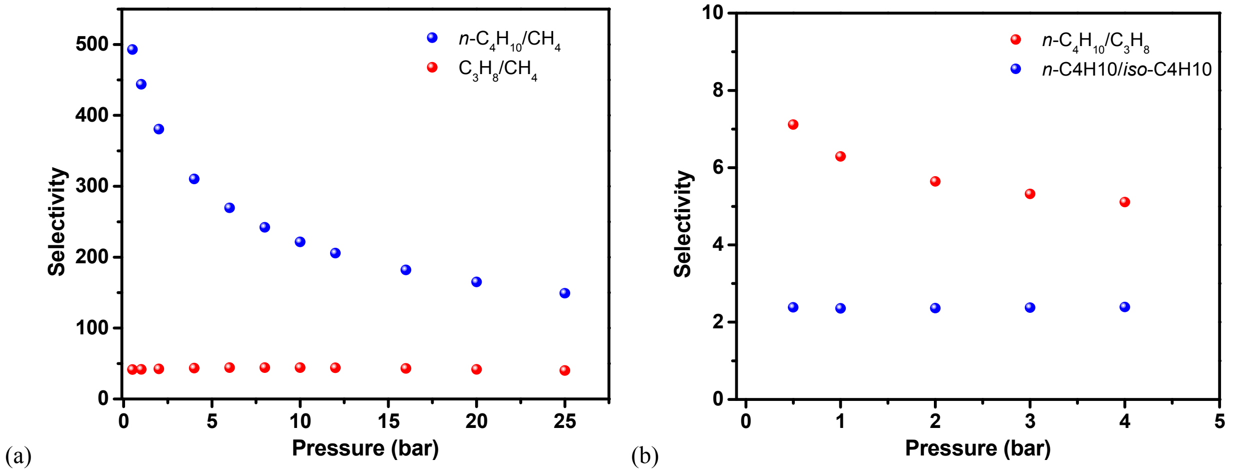

Figure S32. (a) IAST prediction of n-C_4H_{10}/CH_4: 05/95 and C_3H_8/CH_4: 05/95 selectivity for Y-**ftw**-MOF-2. (b) IAST selectivity prediction for Y-**ftw**-MOF-2 for n-C_4H_{10}/C_3H_8: 50/50 and n-C_4H_{10}/ iso-C_4H_{10}: 50/50 mixtures.

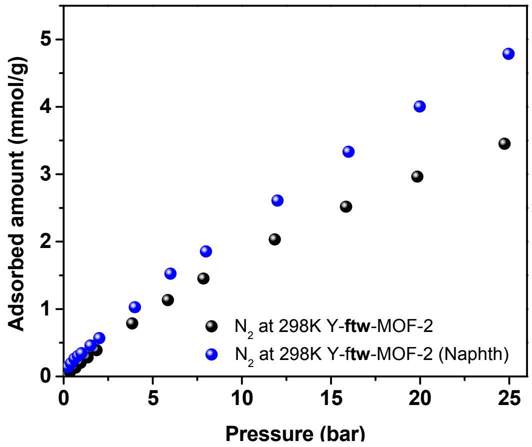

Figure S33. N_2 adsorption isotherm comparison of Y-**ftw**-MOF-2 (black) and Y-**ftw**-MOF-2 (Naphth) (blue) at 298 K.

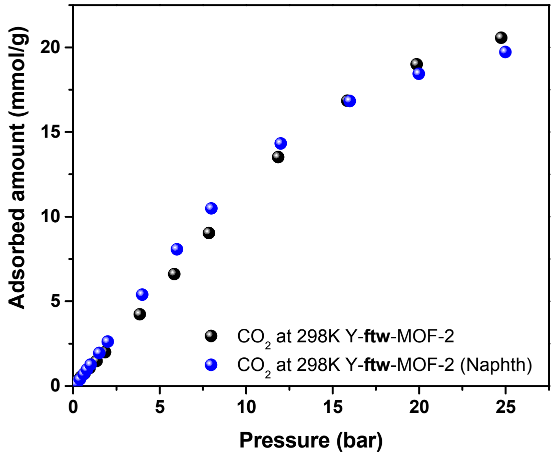

Figure S34. CO_2 adsorption isotherm comparison of Y-**ftw**-MOF-2 (black) and Y-**ftw**-MOF-2 (Naphth) (blue) at 298 K.

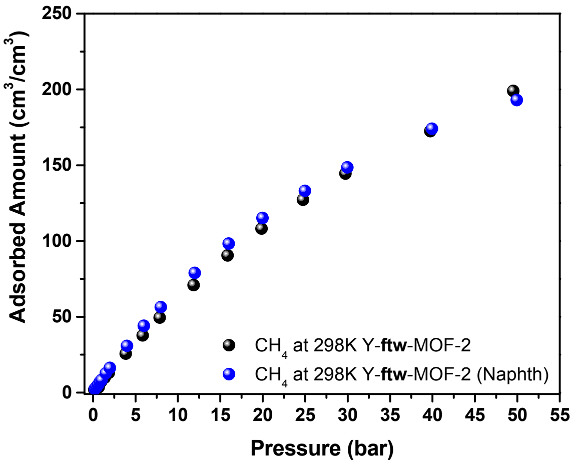

Figure S35. CH_4 adsorption isotherm comparison of Y-**ftw**-MOF-2 (black) and Y-**ftw**-MOF-2 (Naphth) (blue) at 298 K.

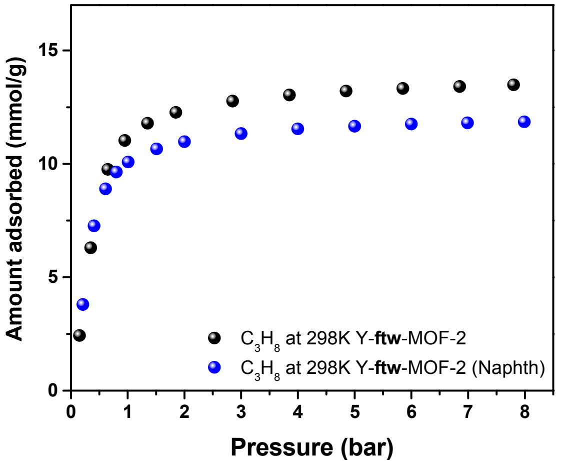

Figure S36. C_3H_8 adsorption isotherm comparison of Y-**ftw**-MOF-2 (black) and Y-**ftw**-MOF-2 (Naphth) (blue) at 298 K.

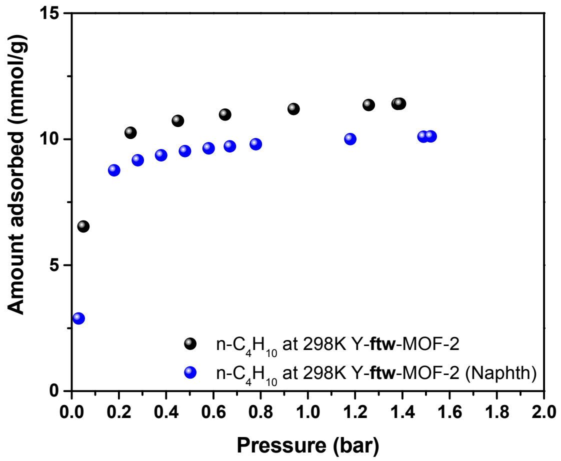

Figure S37. *n*-C_4H_{10} adsorption isotherm comparison of Y-**ftw**-MOF-2 (black) and Y-**ftw**-MOF-2 (Naphth) (blue) at 298 K.

Organic Synthesis:

Preparations: 1,2,4,5-Tetrakis[(4-carboxy)phenoxymethyl]benzene (TetPOMB), 3,3'',5,5''-tetrakis(4-carboxyphenyl)-p-terphenyl (TCPT), 3',3'',5',5''-tetrakis(4-carboxyphenyl)-1,4-diphenylnaphthalene (TCDPN), 3',3'',5',5''-tetrakis(4-carboxyphenyl)-9,10-diphenylanthracene (TCDPA), and 3,3'',5,5''-tetrakis[2-(4-carboxyphenyl)ethynyl]-p-terphenyl (TCEPT) were synthesized in our lab as described below. All other chemicals and solvents were used as received unless otherwise stated from Fisher Scientific, Acros Organics, Combi-Blocks, Sigma-Aldrich, or TCI America. DMF was dried over CaH_2. DI water = deionized water. ^1H and ^{13}C NMR spectra were recorded at room temperature with Bruker Avance 500 and 600 MHz spectrometers using $CDCl_3$ or DMSO-d_6 as the solvents, and referenced to the corresponding solvent peaks (7.26 and 77.16 ppm for $CDCl_3$, and 2.50 and 39.52 ppm for DMSO-d_6, respectively).

Synthesis of 1,2,4,5-Tetrakis[(4-carboxy)phenoxymethyl]benzene (TetPOMB):

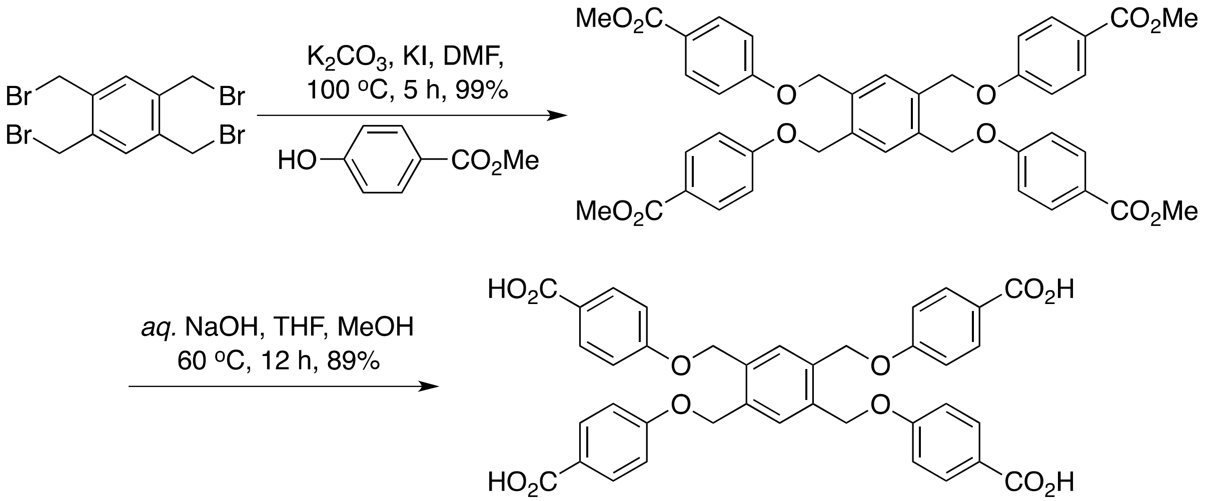

Scheme S1: Synthesis of 1,2,4,5-Tetrakis[(4-carboxy)phenoxymethyl]benzene (TetPOMB)

Preparation of 1,2,4,5-tetrakis[(4-methoxycarbonyl)phenoxymethyl]benzene: The product was synthesized according to the reported procedure.[17] Methyl 4-hydroxybenzoate (9.13 g, 60 mmol) was dissolved in DMF (250 ml). A catalytic amount of KI (74 mg, 4.4 mmol) was added to the solution followed by K_2CO_3 (36 g, 260 mmol). The solution was then heated to 100 °C for 1 hour. 1,2,4,5-Tetrakis(bromomethyl)benzene (3.6 g, 8 mmol) was dissolved in DMF (20 ml), added dropwise to the mixture and heated at 100 °C for 4 hours and then cooled to room temperature. Approximately 800 ml of water was added to the solution to produce a precipitate, which was filtered, washed thoroughly with water and dried on air at suction to give 5.8 g (99 %) of the white solid. ^1H NMR (600 MHz, $CDCl_3$): δ = 7.97 (dd, *J*=2, *J*=6.9, 8H), 7.67 (s, 2H), 6.95 (dd, *J*=2, *J*=6.9, 8H), 5.23 (s, 8H), 3.89 (s, 3H).

Preparation of 1,2,4,5-tetrakis[(4-carboxy)phenoxymethyl]benzene: The above tetramethyl ester (5.8 g, 7.9 mmol) was added to a round bottom flask containing tetrahydrofuran (150 ml) and methanol (50 mL). An aq. NaOH solution (4.9 g, 120 mmol in 200 ml H_2O) was added to this mixture and then heated at 60 °C for 12 h. The solution was concentrated, diluted with water to dissolve the salts, washed with 2 x 70 ml AcOEt (discarded), then acidified to pH = 1 using 6N HCl. The precipitate was separated by filtration, washed thoroughly with water and dried on air at suction to yield 5.0 g (89 %) of white solid. ^1H NMR (600 MHz, DMSO-d_6): δ = 12.6 (bs, 4H), 7.88 (d, *J*=8.9, 8H), 7.74 (s, 2H), 7.10 (d, *J*=8.9, 8H), 5.33 (s, 8H). ^{13}C NMR (150 MHz, DMSO-d_6): δ = 166.9 (Cq), 161.7 (Cq), 134.8 (Cq), 131.3, 129.0, 123.4 (Cq), 114.6, 66.9 (CH_2).

Synthesis of 3,3'',5,5''-tetrakis(4-carboxyphenyl)-p-terphenyl (TCPT):

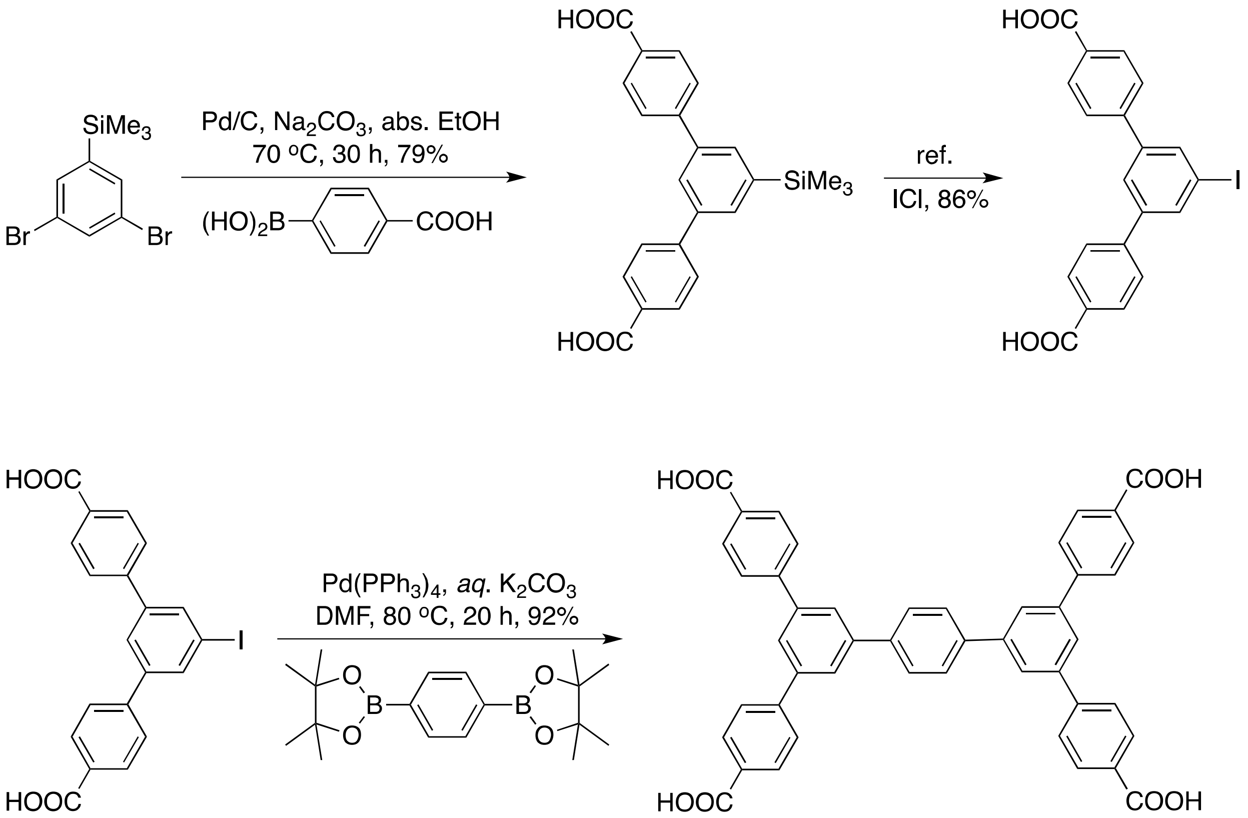

Scheme S2: Synthesis of 3,3'',5,5''-tetrakis(4-carboxyphenyl)-p-terphenyl (TCPT)

Preparation of 1,3-bis(4-carboxyphenyl)-5-trimethylsilylbenzene: Absolute EtOH (180 ml) was placed in a 500 ml round-bottom flask sealed with septum, the flask was evacuated/backfilled with argon 3x. 1,3-Dibromo-5-trimethylsilylbenzene (3.08 g; 10 mmol), 4-carboxyphenylboronic acid (3.66 g; 22 mmol), 5% Pd/C (1.4 g) and sodium carbonate (8.48 g; 80 mmol) were then added, the flask was evacuated/backfilled with argon 2x and heated at 70 °C for 30 h with vigorous stirring.

It was cooled to room temperature and the mixture was diluted with water (500 ml) to dissolve salts, then filtered through paper, filter cake was washed with water, the filtrate washed once with hexane (100 ml, discarded), then acidified to pH = 1 with 2 N HCl (effervescence!) and the precipitate was filtered, washed with water, dried at suction overnight to give 3.11 g (79%) of white powder in good purity. The NMR data match the reported values.[18]

Preparation of 1,3-bis(4-carboxyphenyl)-5-iodobenzene: It was obtained by treatment of 1,3-bis(4-carboxyphenyl)-5-trimethylsilylbenzene with iodine monochloride in DMF according to the reported procedure in up to 86% yield.[18]

Preparation of 3,3'',5,5''-tetrakis(4-carboxyphenyl)-p-terphenyl: General procedure for Suzuki coupling under aqueous conditions:[18] The mixture of DMF (110 ml) and aq. potassium carbonate (9.7 g, 70 mmol in 30 ml H_2O) was placed in a 250 ml round-bottom flask sealed with septum, the flask was evacuated/backfilled with argon 3x, then bubbled with argon for 1.5 h. To the mixture were added 1,3-bis(4-carboxyphenyl)-5-iodobenzene (2.23 g; 5 mmol), 1,4-benzenediboronic acid bis(pinacol) ester (0.829 g; 2.5 mmol) and tetrakis(triphenylphosphine)palladium (0) (0.29 g; 0.25 mmol), the flask was evacuated/backfilled with argon 3x and heated at 80 °C for 20 h with vigorous stirring.

It was cooled to room temperature and the mixture was diluted with water to 400 ml total volume, filtered through paper if necessary, the filtrate washed with ethyl acetate (2x 60 ml, discarded). Then it was acidified with 6N HCl and the precipitate collected by centrifugation (6000 rpm). It was washed several times by the repeated centrifugation with water (2x), then with ethanol or acetone (2x). Finally it was suspended in ethanol, concentrated using rotavap and the residue was dried at 65 °C overnight to yield 1.64 g (92 %) of the product as a tan solid in sufficient purity. ^{1}H NMR (600 MHz, DMSO-d6) δ = 13.0 (b, 4H), 8.09-8.05 (m, 26H)· ^{13}C NMR (125 MHz, DMSO-d6) δ = 167.2 (Cq), 144.1 (Cq), 141.4 (Cq), 140.7 (Cq), 139.3 (Cq), 130.0 (Cq), 129.9, 127.9, 127.4, 125.4, 125.0. Additionally, the peaks of solvents used for centrifugation (ethanol and acetone) could be seen in the spectra.

Synthesis of 3',3'',5',5''-tetrakis(4-carboxyphenyl)-1,4-diphenylnaphthalene (TCDPN):

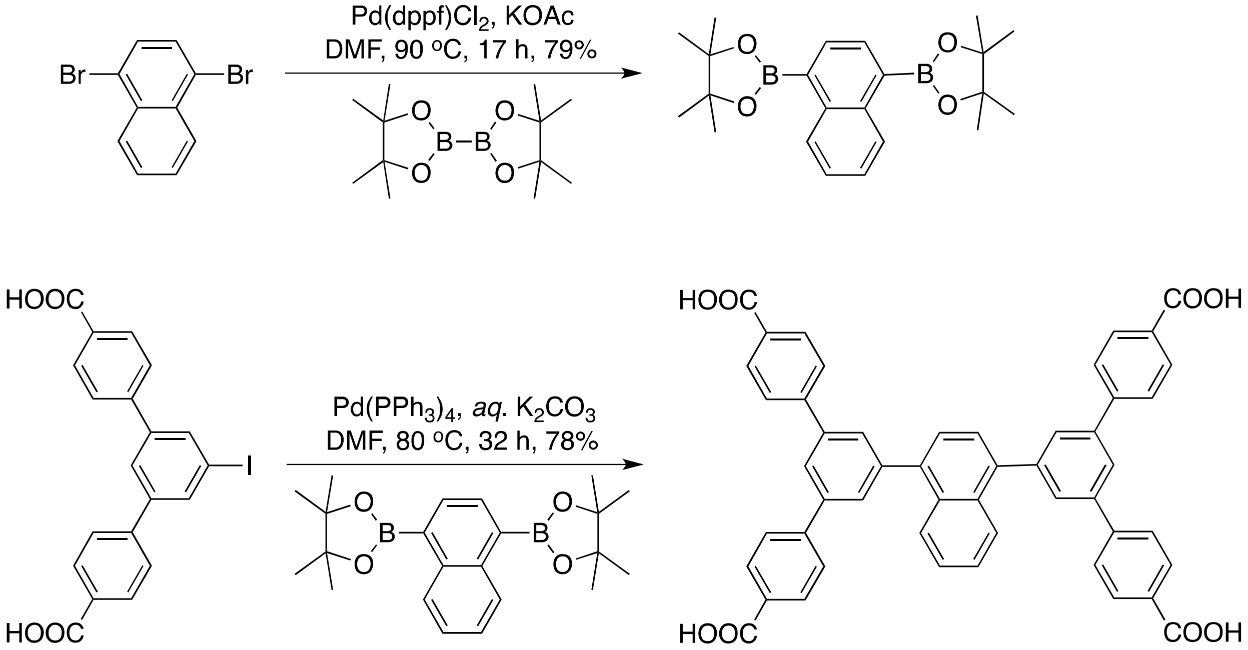

Scheme S3: Synthesis of 3',3'',5',5''-tetrakis(4-carboxyphenyl)-1,4-diphenylnaphthalene (TCDPN)\

Preparation of 1,4-naphthalenediboronic acid bis(pinacol) ester: The compound was synthesized according to the reported general procedure.[19] Dried DMF (20 ml) was placed in a 150 ml oven-dried round-bottom flask sealed with septum, the flask was evacuated/backfilled with argon 3x, then bubbled with argon for 0.5 h. 1,4-Dibromonaphthalene (1 g; 3.5 mmol), bis(pinacolato)diboron (2.13 g; 8.4 mmol), [1,1′-bis(diphenylphosphino)ferrocene]dichloropalladium(II) (0.256 g; 0.35 mmol) and anhydrous potassium acetate (2.06 g; 21 mmol) were added, the flask was evacuated/backfilled with argon 3 x and heated at 90 °C for 17 h with vigorous stirring.

It was cooled to room temperature and the mixture was diluted with 70 ml ethyl acetate and poured on 100 ml DI water. The phases were separated, and the water phase was backextracted with 50 ml EtOAc. The combined organics were washed with 2x 50 ml brine (filtered through paper if necessary to facilitate separation of the phases), and dried with $MgSO_4$. After filtration, the residue was concentrated with a small amount of silica gel, applied on top of a silica gel column and chromatographed, eluting with hexane to 20% ethyl acetate in hexane to yield 1.05 g (79%) of the product as a white powder. ^{1}H NMR (600 MHz, $CDCl_3$) δ = 8.76-8.74 (m, 2H), 8.02 (s, 2H), 7.53-7.50 (m, 2H), 1.43 (s, 24H). ^{13}C NMR (150 MHz, $CDCl_3$) δ = 136.7 (Cq), 134.5, 128.8, 126.0, 84.0 (Cq), 25.1.

Preparation of 3',3'',5',5''-tetrakis(4-carboxyphenyl)-1,4-diphenylnaphthalene: The general procedure for Suzuki-Miyaura coupling under aqueous conditions using 1,4-naphthalenediboronic acid bis(pinacol) ester (1.33 g, 3.5 mmol) was applied to give 2.13 g (78%) of the product as a cream solid. ^{1}H NMR (500 MHz, DMSO-d_6) δ = 13.1 (b, 4H), 8.18 (s, 2H), 8.08-8.02 (m, 18H), 7.92-7.91 (d, *J*=1.3, 4H), 7.76 (s, 2H), 7.58 (m, 2H). ^{13}C NMR (125 MHz, DMSO-d_6) δ = 167.2 (Cq), 143.9 (Cq), 141.6 (Cq), 140.3 (Cq), 139.0 (Cq), 131.4 (Cq), 130.1, 128.4, 127.5, 127.0, 126.8, 126.0, 125.1. Additionally, the peaks of solvents used for centrifugation (ethanol and acetone) could be seen in the spectra.

Synthesis of 3',3'',5',5''-tetrakis(4-carboxyphenyl)-9,10-diphenylanthracene (TCDPA):

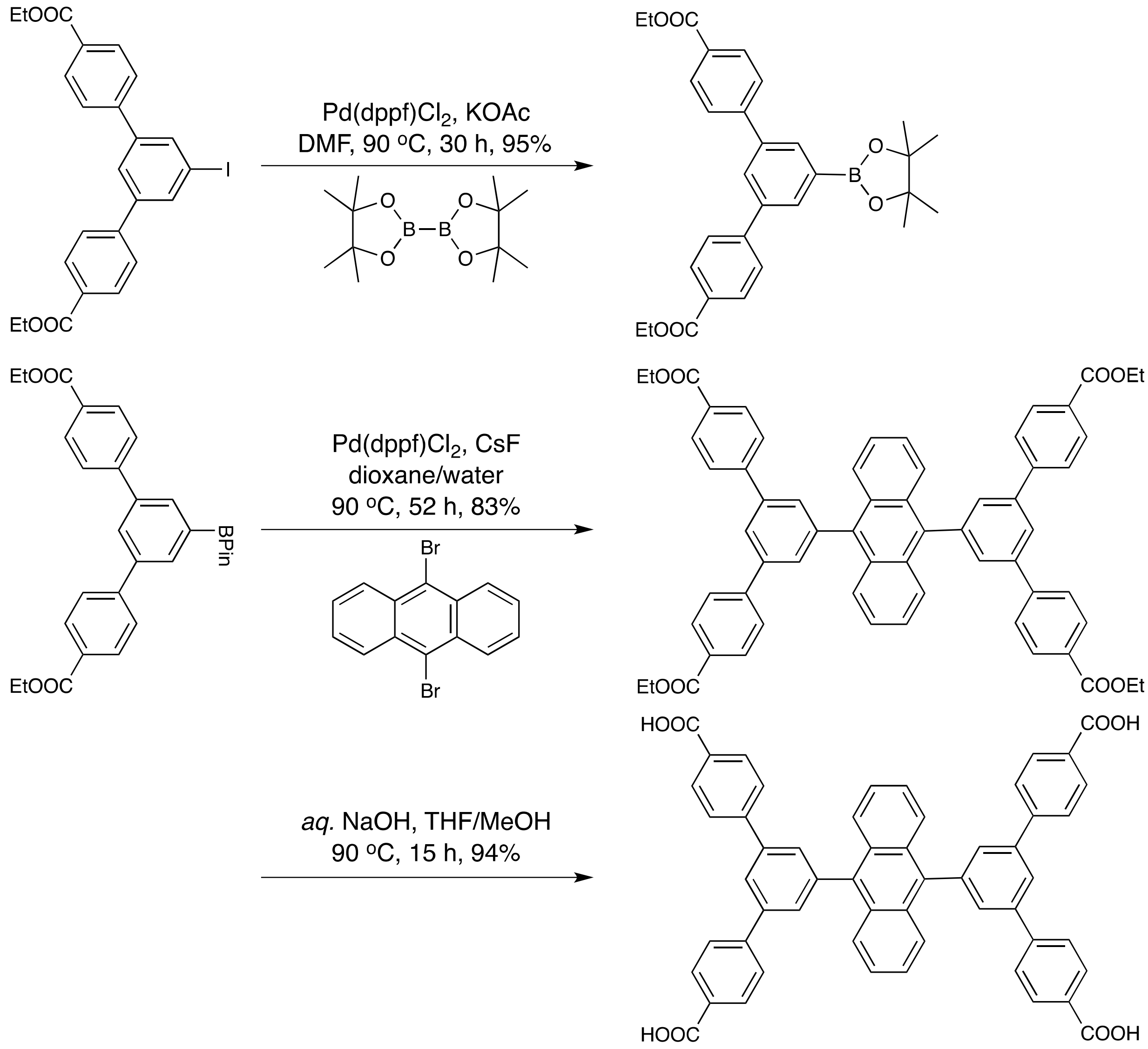

Scheme S4: Synthesis of 3',3'',5',5''-tetrakis(4-carboxyphenyl)-9,10-diphenylanthracene (TCDPA)

Preparation of 1,3-bis(4-ethoxycarbonylphenyl)-5-iodobenzene: It was obtained according to the reported procedure[20] and purified by column chromatography (hexane to ethyl acetate) to give an orange solid, 4.74 g, 95%. ^{1}H NMR (600 MHz, $CDCl_3$) δ = 8.13 (d, *J*=8.4, 4H), 7.97 (d, *J*=1.6, 2H), 7.77 (t, *J*=1.6, 1H), 7.66 (d, *J*=8.4, 4H), 4.41 (q, *J*=7.1, 4H), 1.43 (t, *J*=7.1, 6H). ^{13}C NMR (150 MHz, $CDCl_3$) δ = 166.4 (Cq), 143.8 (Cq), 142.9 (Cq), 135.9, 130.4, 130.2 (Cq), 127.3, 125.9, 95.5 (Cq), 61.3 (CH_2), 14.5 (CH_3).

Preparation of 3,5-bis(4-ethoxycarbonylphenyl)benzeneboronic acid pinacol ester: It was obtained in similar manner as 1,4-naphthalenediboronic acid bis(pinacol) ester. Dry DMF (45 ml) was placed in a 150 ml oven-dried round-bottom flask sealed with septum, and the flask was evacuated/backfilled with argon 3x. 1,3-Bis(4-ethoxycarbonylphenyl)-5-iodobenzene (4.72 g; 9.44 mmol), bis(pinacolato)diboron (2.88 g; 11.3 mmol), [1,1′-bis(diphenylphosphino)ferrocene]dichloropalladium(II) (0.345 g; 0.47 mmol) and anhydrous potassium acetate (2.78 g; 28.3 mmol) were added, the flask was evacuated/backfilled with argon 3x and heated at 90 °C for 30 h with vigorous stirring.

It was cooled to room temperature and the mixture was diluted with 200 ml ethyl acetate and poured on 100 ml DI water. The phases were separated, organics were washed with brine (3 x 100 ml), and dried with $MgSO_4$. After filtration, the residue was applied on top of a silica gel column and chromatographed, eluting with hexane to 50% ethyl acetate in hexane to yield 4.49 g (95%) of the product as a greenish solid in sufficient purity (contains some bis(pinacolato)diboron as an impurity). ^{1}H NMR (600 MHz, $CDCl_3$) δ = 8.11 (d, *J*=8.3, 4H), 8.08 (d, *J*=1.7, 2H), 7.93 (t, *J*=1.6, 1H), 7.75 (d, *J*=8.3, 4H), 4.41 (q, *J*=7.1, 4H), 1.42 (t, *J*=7.1, 6H), 1.39 (s, 12H). ^{13}C NMR (150 MHz, $CDCl_3$) δ = 166.6 (Cq), 145.3 (Cq). 140.4 (Cq), 133.4, 130.2, 129.6 (Cq), 129.1, 127.4, 84.3 (Cq), 61.2 (CH_2), 25.0 (CH_3), 14.5 (CH_3).

Preparation of 3',3'',5',5''-tetrakis(4-ethoxycarbonylphenyl)-9,10-diphenylanthracene: The compound was synthesized according to the modified literature procedure.[21,22] In a 500 ml Schlenk tube, a mixture of 3,5-bis(4-ethoxycarbonylphenyl)benzeneboronic acid pinacol ester (1.62 g, 3.25 mmol) and CsF (1.36 g, 8.9 mmol) in dioxane (70 ml) and DI water (30 ml) was prepared and the tube was evacuated/backfilled with argon 3x, then 9,10-dibromoanthracene (0.5 g, 1.48 mmol) and [1,1'-bis(diphenylphosphino)ferrocene]dichloropalladium(II) (0.054 g, 0.07 mmol) were added, the tube was evacuated/backfilled with argon 2x, then sealed and heated at 90 °C (oil bath) for 52 h with vigorous stirring. Yellow suspension is quickly forming. The reaction mixture was then cooled and left overnight. The precipitated solid was filtered on paper, washed with small volume of dioxane, followed by hexane and water, then dried at suction overnight to give greyish solid in sufficient purity, 1.14 g (83%). ^{1}H NMR (600 MHz, $CDCl_3$) δ = 8.16 (d, J=8.4, 8H), 8.09 (m, 2H), 7.86 (m, 4H), 7.82 (m, 12H), 7.42 (m, 4H), 4.41 (q, *J*=7.1, 8H), 1.43 (t, *J*=7.1,12H). ^{13}C NMR (150 MHz, $CDCl_3$) δ = 166.6 (Cq), 144.9 (Cq), 141.2 (Cq), 140.6 (Cq), 136.7 (Cq), 130.4, 130.05 (Cq), 129.99, 129.87 (Cq), 127.4, 127.0, 125.7, 125.6, 61.2 (CH_2), 14.5 (CH_3).

Preparation of 3',3'',5',5''-tetrakis(4-carboxyphenyl)-9,10-diphenylanthracene:[23] It was obtained in a similar manner as 1,2,4,5-tetrakis[(4-carboxy)phenoxymethyl]benzene. The intermediate tetraester (1.12 g, 1.2 mmol) was added to a round bottom flask containing tetrahydrofuran (80 ml) and methanol (10 ml). An aq. NaOH solution (0.6 g, 14.6 mmol in 30 ml H_2O) was added to this mixture and then heated at 90 °C for 15 h. The solution was cooled, diluted with water (200 ml), washed with Et_2O (50 ml, discarded) and EtOAc (50 ml, discarded), and filtered through Celite®, washing with additional water. The filtrate was acidified using 2N HCl, and the precipitate was separated by filtration, washed thoroughly with water and dried at suction to give yellow solid in sufficient purity, 0.9 g (94%). ^{1}H NMR (600 MHz, DMSO-d_6): δ = 13.0 (bs, 4H), 8.35 (s, 2H), 8.06 (s, 16H), 7.89 (s, 4H), 7.78 (m, 4H), 7.49 (m, 4H). ^{13}C NMR (150 MHz, DMSO-d_6): δ = 167.1 (Cq), 143.6 (Cq), 140.4 (Cq), 139.9 (Cq), 136.2 (Cq), 130.1 (Cq), 130.0, 129.4 (Cq), 129.2, 127.4, 126.5, 126.0, 125.2.

Synthesis of 3,3'',5,5''-tetrakis[2-(4-carboxyphenyl)ethynyl]-p-terphenyl (TCEPT):

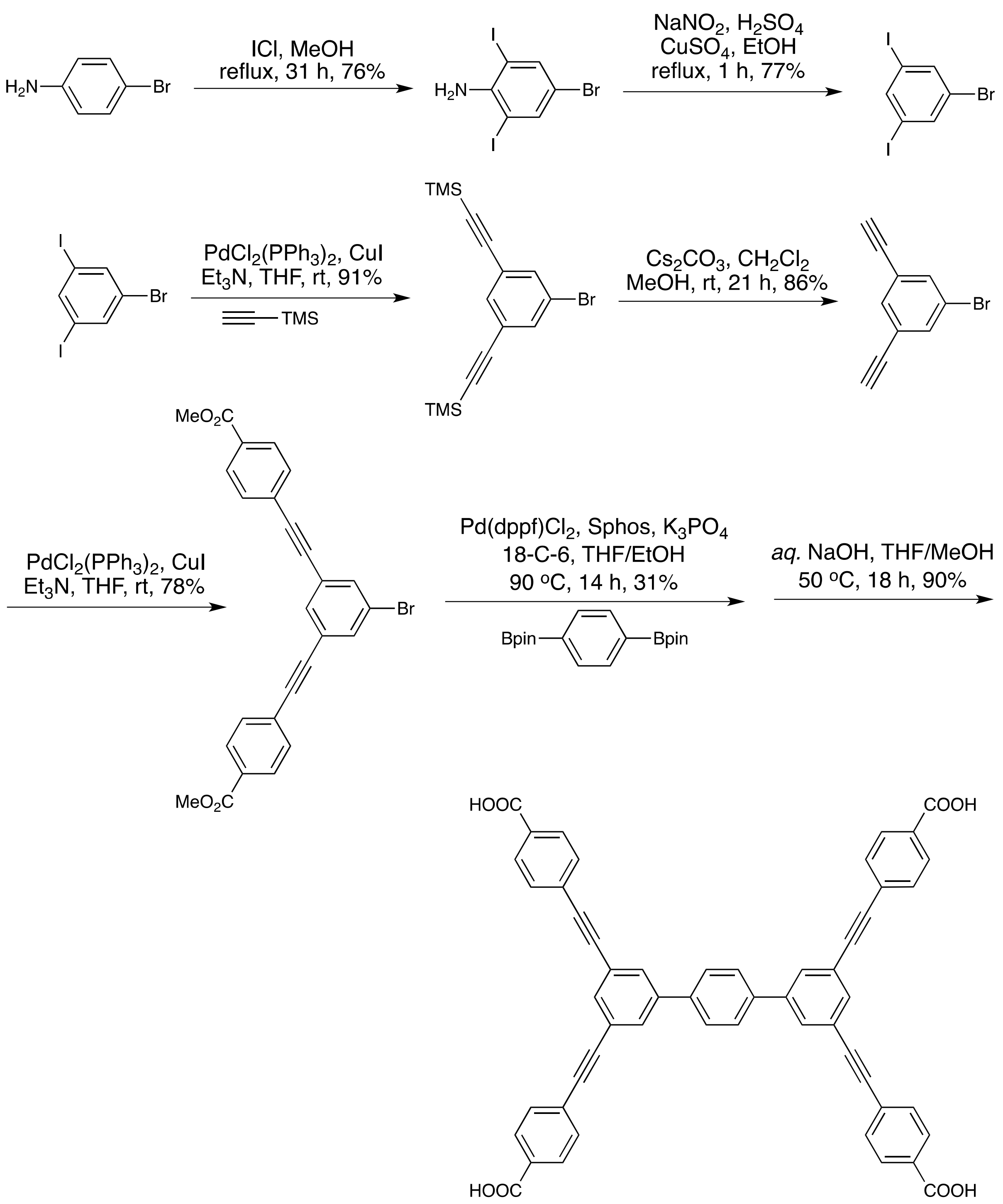

Scheme S5: Synthesis of 3,3'',5,5''-tetrakis[2-(4-carboxyphenyl)ethynyl]-p-terphenyl (TCEPT)

Preparation of 4,6-bromo-2,6-diiodoaniline: It was obtained according to the modified literature procedure.[24] 4-Bromoaniline (6 g, 34.9 mmol) and iodine monochloride (17 g, 104.6 mmol) were refluxed in methanol (140 ml) for 31 h. The mixture was cooled, orange precipitate was filtered on paper, washed with MeOH (2 x 10 ml), then aq. $Na_2S_2O_3$ (100 ml), then thoroughly with DI water and dried on air at suction overnight to give cream solid, 11.32 g (76%) in good purity. NMR data match the reported values.[24]

Preparation of 5-bromo-1,3-diiodobenzene: It was obtained according to the modified literature procedure.[25] 4-Bromo-2,6-diiodoaniline (6 g, 14.2 mmol) was taken in ethanol abs. (100 ml), conc. H_2SO_4 (2 ml) was added, followed by $NaNO_2$ (2.44 g, 35.4 mmol), $CuSO_4$*5H_2O (0.35 g, 1.42 mmol) and heated to reflux with vigorous stirring for 1 h. The mixture was cooled, the precipitate filtered on paper, washed with EtOH abs. (2 x 10 ml), then thoroughly with DI water and dried on air at suction overnight to give cream solid, 4.47 g (77 %) in good purity. NMR data match the reported values.[25]

Preparation of 1-bromo-3,5-bis(trimethylsilylethynyl)benzene: It was obtained according to the reported procedure in sufficient purity as a colorless oil (3.1 g, 91%).[24]

Preparation of 1-bromo-3,5-diethynylbenzene: 1-Bromo-3,5-bis(trimethylsilylethynyl)benzene (3.1 g, 8.9 mmol) was dissolved in 30 ml CH_2Cl_2/60 ml MeOH, Cs_2CO_3 (2.9 g, 8.9 mmol) was added and the whole stirred at rt for 21 h. It was diluted with 100 ml CH_2Cl_2, followed by 80 ml 0.5 N HCl, mixture was shaken and phases were separated, extraction repeated twice with 40 ml CH_2Cl_2, combined organics were dried with Na_2SO_4. It was filtered and concentrated to give yellowish solid, 1.59 g (86%). NMR data match the reported values.[26]

Preparation of 1-bromo-3,5-bis[2-(4-methoxycarbonylphenyl)ethynyl]benzene: It was obtained in a similar manner as 1-bromo-3,5-bis(trimethylsilylethynyl)benzene. A solution of 1-bromo-3,5-diethynylbenzene (1.58 g, 7.74 mmol) in 30 ml THF (stored over CaH_2)/10 ml Et_3N in a 250 ml round-bottom flask sealed with septum was evacuated/backfilled with Ar 3x, then bubbled with Ar for 30 min. Methyl 4-iodobenzoate (4.05 g, 15. 47 mmol), $Pd(PPh_3)_2Cl_2$ (326 mg, 0.46 mmol) and CuI (88 mg, 0.46 mmol) were added, the flask was evacuated/backfilled 3x with Ar, then stirred at rt for 22 h. The mixture was diluted with 150 ml CH_2Cl_2, washed with 3 x 100 ml of std. aq. NH_4Cl, dried with $MgSO_4$. It was filtered and concentrated with a little silica and loaded on top of a silica gel column and eluted with 100% hexane to 100% ethyl acetate to give light brown solid, 2.86 g (78 %). ^{1}H NMR (600 MHz, $CDCl_3$) δ = 8.03 (d, *J*=8.5, 4H), 7.66 (d, *J*=1.3, 2H), 7.64 (t, *J*=1.3, 1H), 7.58 (d, *J*=8.4, 4H), 3.93 (s, 6H). ^{13}C NMR (150 MHz, $CDCl_3$) δ = 166.6 (Cq), 134.6, 133.4, 131.8, 130.2 (Cq), 129.7, 127.2 (Cq), 125.0 (Cq), 122.2 (Cq), 90.6 (Cq), 89.8 (Cq), 52.4 (CH_3).

Preparation of 3,3'',5,5''-tetrakis[2-(4-ethoxycarbonylyphenyl)ethynyl]-p-terphenyl: It was obtained according to the reported general procedure.[20] In a Schlenk tube under argon atmosphere, a mixture of 1-bromo-3,5-bis[2-(4-methoxycarbonylphenyl)ethynyl]benzene (0.502 g, 1.06 mmol), 1,4-benzenediboronic acid bis(pinacol) ester (0.175 g, 0.53 mmol), [1,1'-bis(diphenylphosphino)ferrocene]dichloropalladium(II) (0.038 g, 0.052 mmol), 2-dicyclohexylphosphino-2',6'-dimethoxybiphenyl (Sphos) (0.040 g, 0.1 mmol), finely grinded K_3PO_4 (0.45 g, 2.12 mmol), and 18-crown-6 ether (0.07 g, 0.265 mmol) in a mixture of degassed THF (20 ml) and EtOH (10 ml) was prepared and reacted at 90 °C for 14 h with vigorous stirring. The reaction mixture was then filtered through Celite® and the volatiles removed. The residue was concentrated with a little silica and loaded on top of the silica gel column and eluted with 100% hexane to 100% ethyl acetate to give light brown solid, 0.153 g (31 %) in sufficient purity (contains some pinacol). ^{1}H NMR (600 MHz, $CDCl_3$) δ = 8.05 (d, *J*=8.2, 8H), 7.81 (m, 2H), 7.74 (s, 6H), 7.62 (d, *J*=8.2, 8H), 4.40 (q, *J*=7.1, 8H), 1.41 (t, *J*=7.1, 12H). ^{13}C NMR (150 MHz, $CDCl_3$) δ = 166.1 (Cq), 141.2 (Cq), 139.1 (Cq), 133.8, 131.7, 130.5, 130.3 (Cq), 129.7, 127.8, 127.5 (Cq), 124.0 (Cq), 91.2 (Cq), 89.8 (Cq), 61.3 (CH_2), 14.5 (CH_3).

Preparation of 3,3'',5,5''-tetrakis[2-(4-carboxyphenyl)ethynyl]-p-terphenyl: It was obtained in a similar manner as 1,2,4,5-tetrakis[(4-carboxy)phenoxymethyl]benzene. The intermediate tetraester (0.15 g, 0.16 mmol) was added to a round bottom flask containing tetrahydrofuran (35 ml) and methanol (15 ml). An aq. NaOH solution (0.205 g, 5.13 mmol in 20 ml H_2O) was added to this mixture and then heated at 50 °C for 18 h. The solution was concentrated, diluted with water (100 ml), washed with 50 ml AcOEt (discarded), then acidified using 2N HCl. The precipitate was collected by centrifugation (4500 rpm/3 min). It was washed by a repeated centrifugation with water (2 x). Finally it was suspended in ethanol, and concentrated on rotavap to give brown solid in sufficient purity, 0.12 g (90 %). ^{1}H NMR (500 MHz, DMSO-d_6): δ = 13.2 (bs, 4H), 8.05 (s, 4H), 8.00 (d, *J*=8.2, 8H), 7.96 (m, 4H), 7.83 (m, 2H), 7.73 (d, *J*=8.2, 8H). ^{13}C NMR (125 MHz, DMSO-d_6): δ = 166.8 (Cq), 140.6 (Cq), 137.8 (Cq), 133.3, 131.8, 130.9 (Cq), 130.2, 129.7, 127.7, 126.3 (Cq), 123.4 (Cq), 90.9 (Cq), 89.7 (Cq). Additionally, the peaks of solvent used for centrifugation (ethanol) could be seen in the spectra.

Selected copies of ^{1}H and ^{13}C spectra.

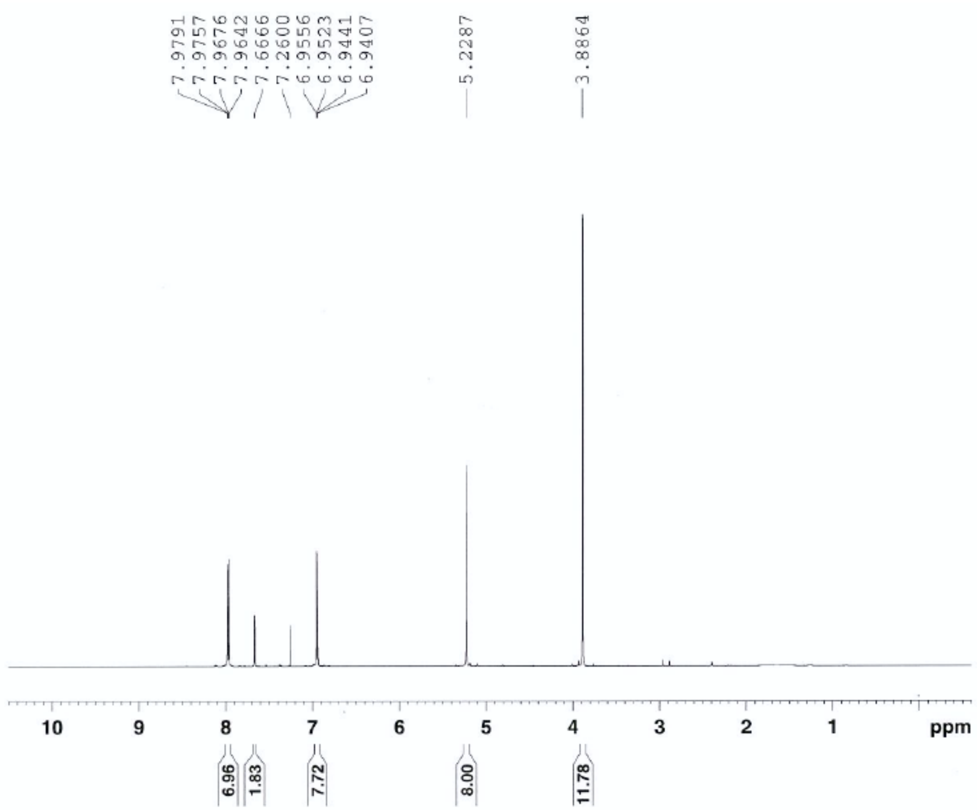

Figure S38: ^{1}H NMR spectrum of 1,2,4,5-tetrakis[(4-methoxycarbonyl)phenoxymethyl]benzene (600 MHz, $CDCl_3$).

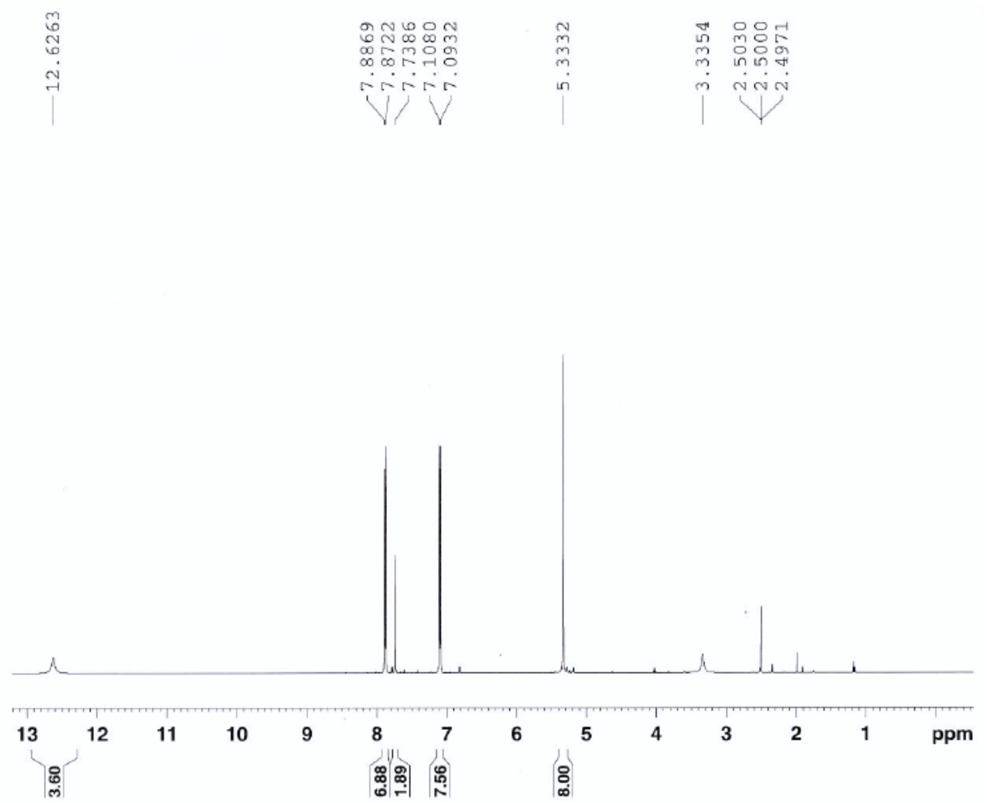

Figure S39: ^{1}H NMR spectrum of 1,2,4,5-tetrakis[(4-carboxy)phenoxymethyl]benzene (600 MHz, DMSO-d_6).

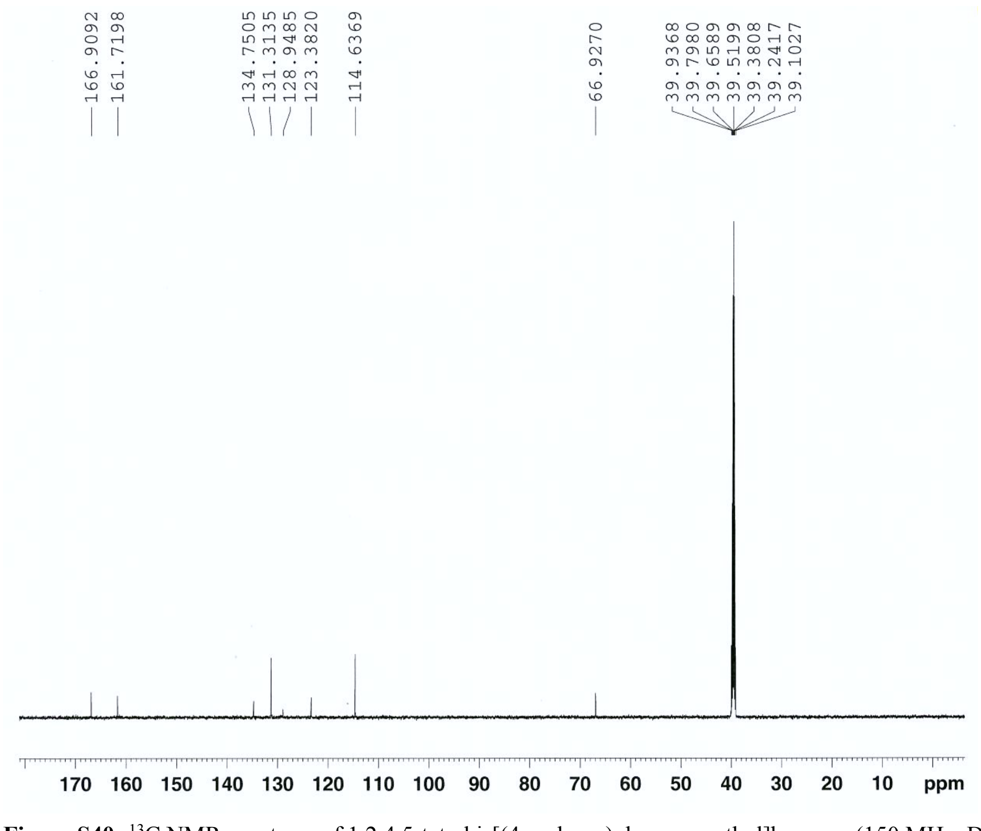

Figure S40: ^{13}C NMR spectrum of 1,2,4,5-tetrakis[(4-carboxy)phenoxymethyl]benzene (150 MHz, DMSO-d_6).

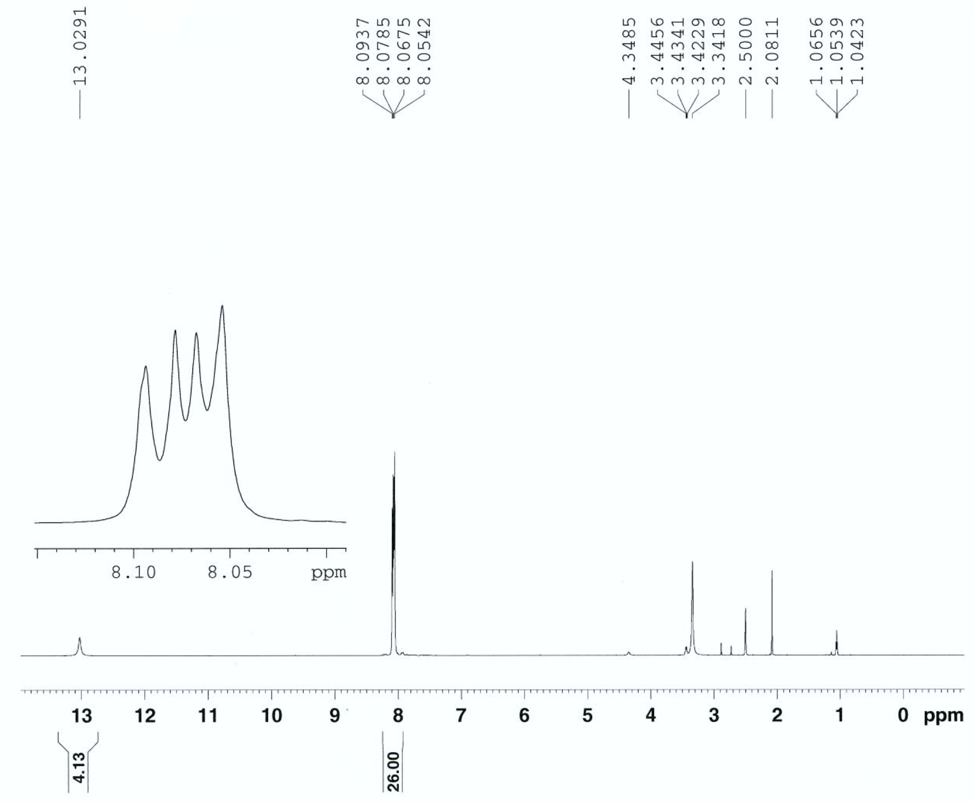

Figure S41: ^{1}H NMR spectrum of 3,3'',5,5''-tetrakis(4-carboxyphenyl)-p-terphenyl (600 MHz, DMSO-d_6).

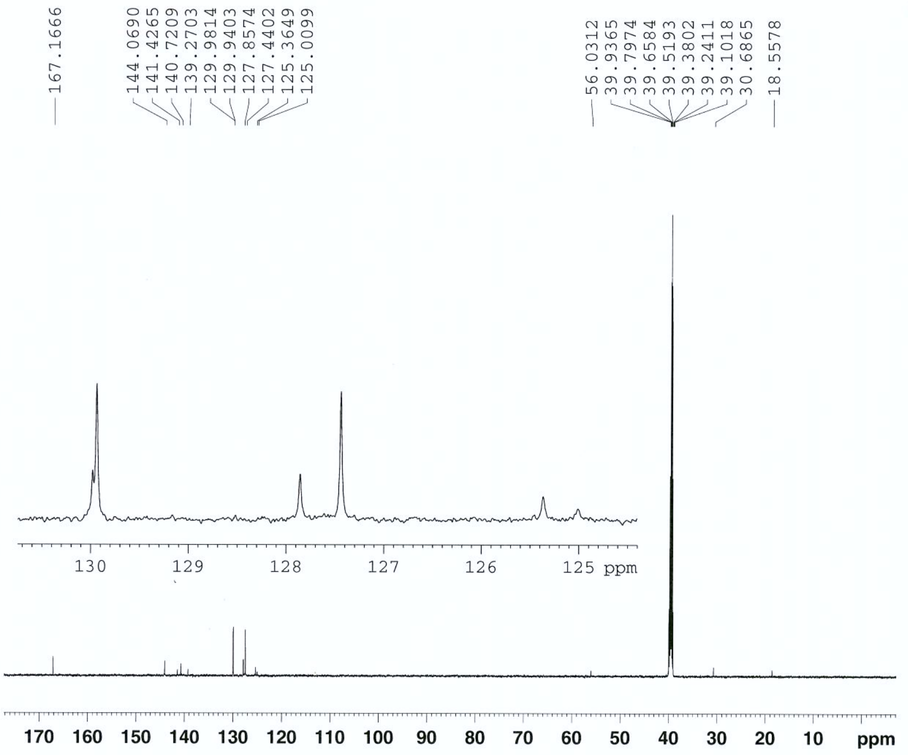

Figure S42: ^{13}C NMR spectrum of 3,3'',5,5''-tetrakis(4-carboxyphenyl)-p-terphenyl (150 MHz, DMSO-d_6).

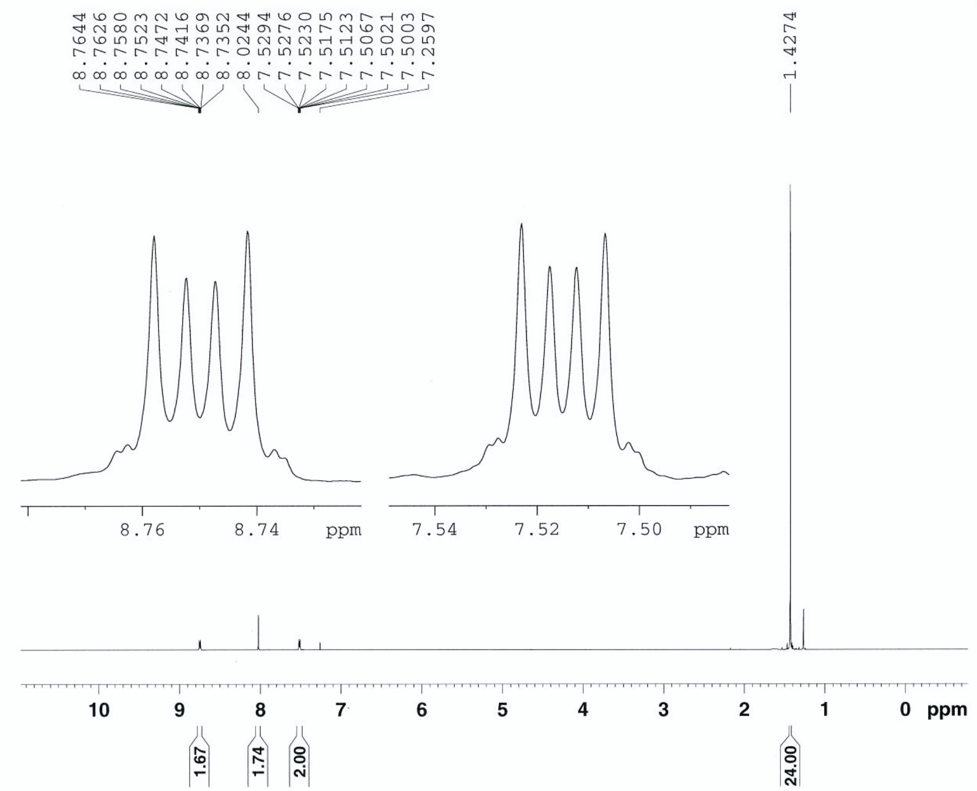

Figure S43: ^{1}H NMR spectrum of 1,4-naphthalenediboronic acid bis(pinacol) ester (500 MHz, $CDCl_3$).

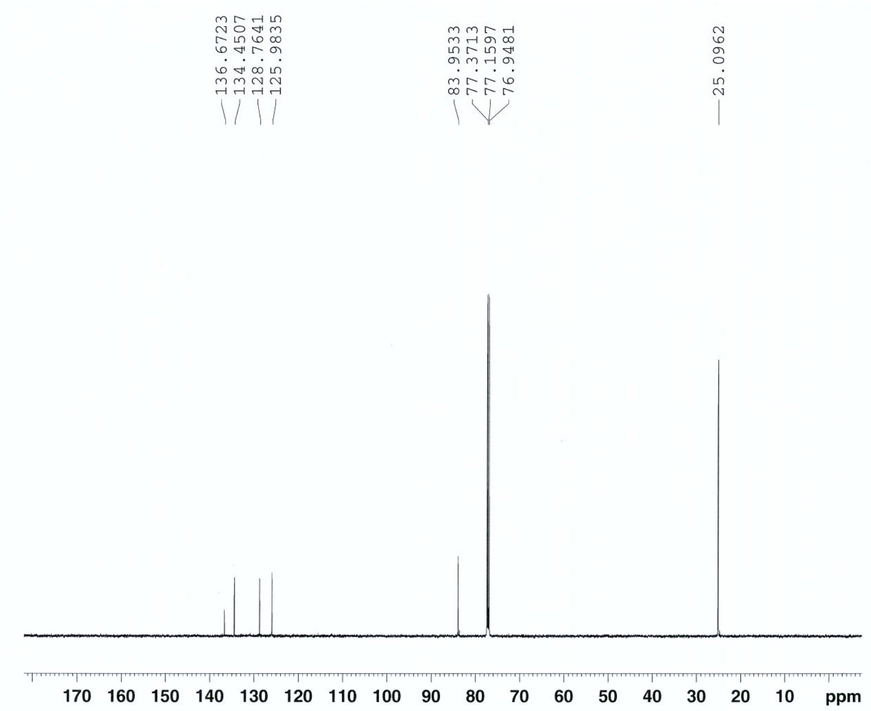

Figure S44: ^{13}C NMR spectrum of 1,4-naphthalenediboronic acid bis(pinacol) ester (125 MHz, $CDCl_3$).

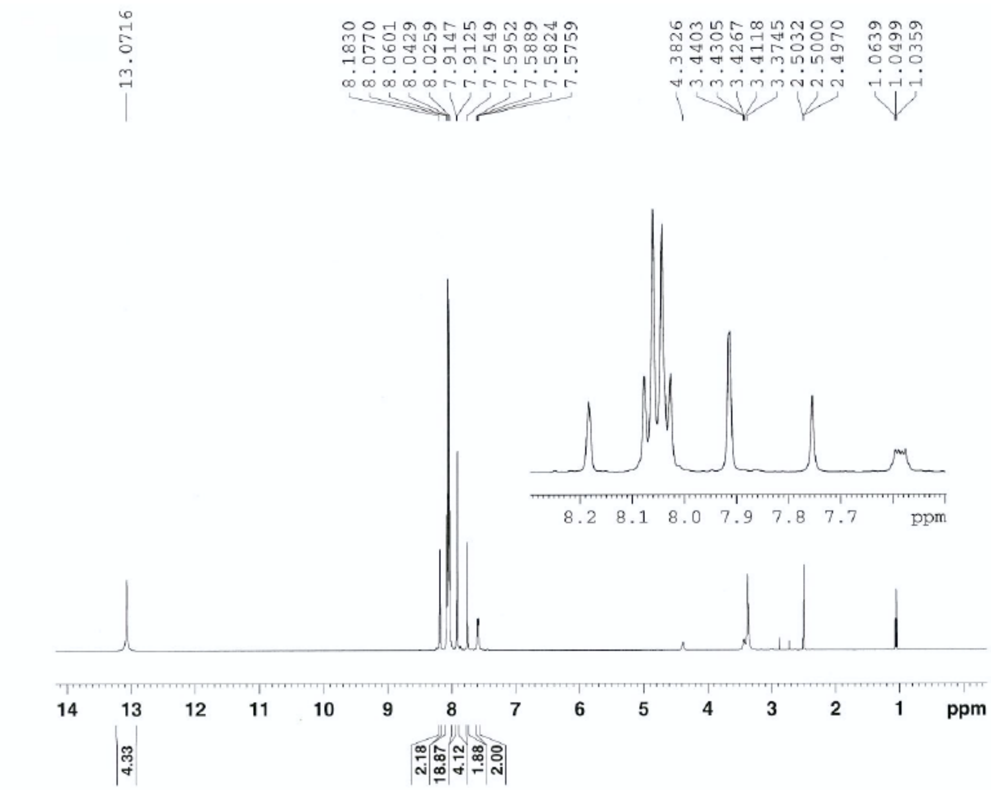

Figure S45: ^{1}H NMR spectrum of 3',3'',5',5''-tetrakis(4-carboxyphenyl)-1,4-diphenylnaphthalene (500 MHz, DMSO-d_6).

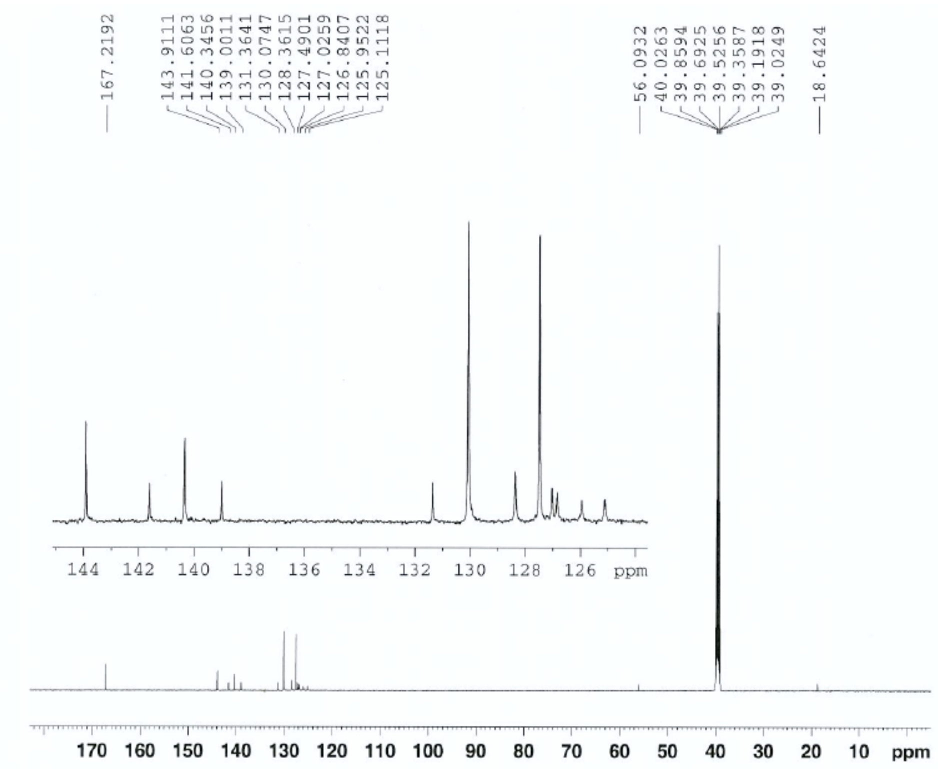

Figure S46: ^{13}C NMR spectrum of 3',3'',5',5''-tetrakis(4-carboxyphenyl)-1,4-diphenylnaphthalene (125 MHz, DMSO-d_6).

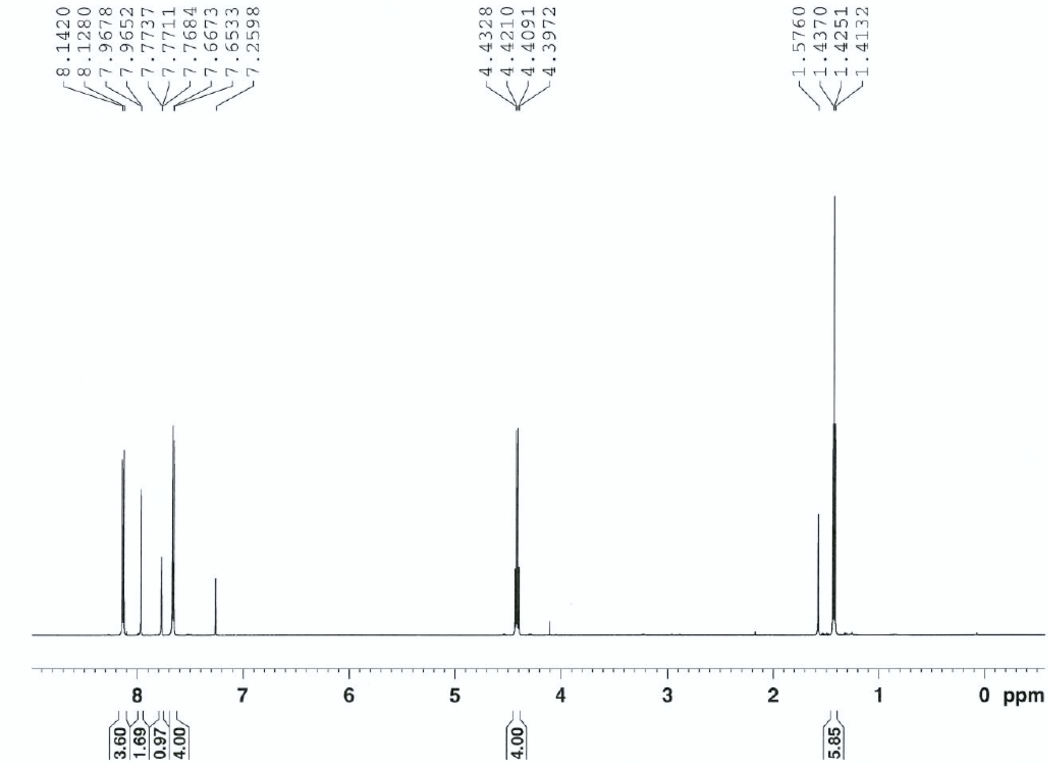

Figure S47: ^{1}H NMR spectrum of 1,3-bis(4-ethoxycarbonylphenyl)-5-iodobenzene (600 MHz, $CDCl_3$).

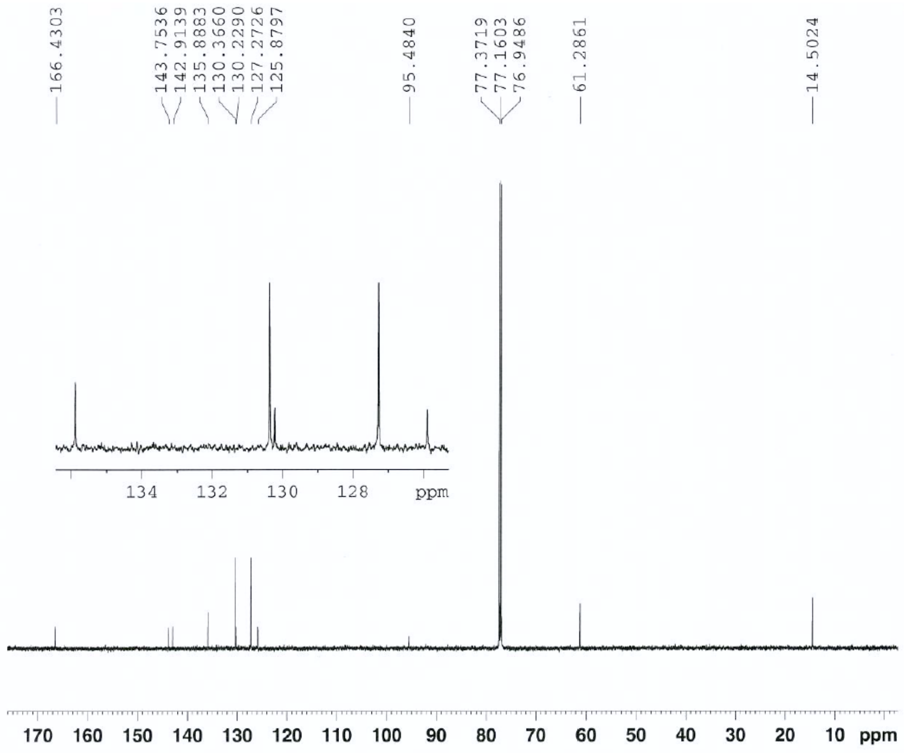

Figure S48: ^{13}C NMR spectrum of 1,3-bis(4-ethoxycarbonylphenyl)-5-iodobenzene (150 MHz, $CDCl_3$).

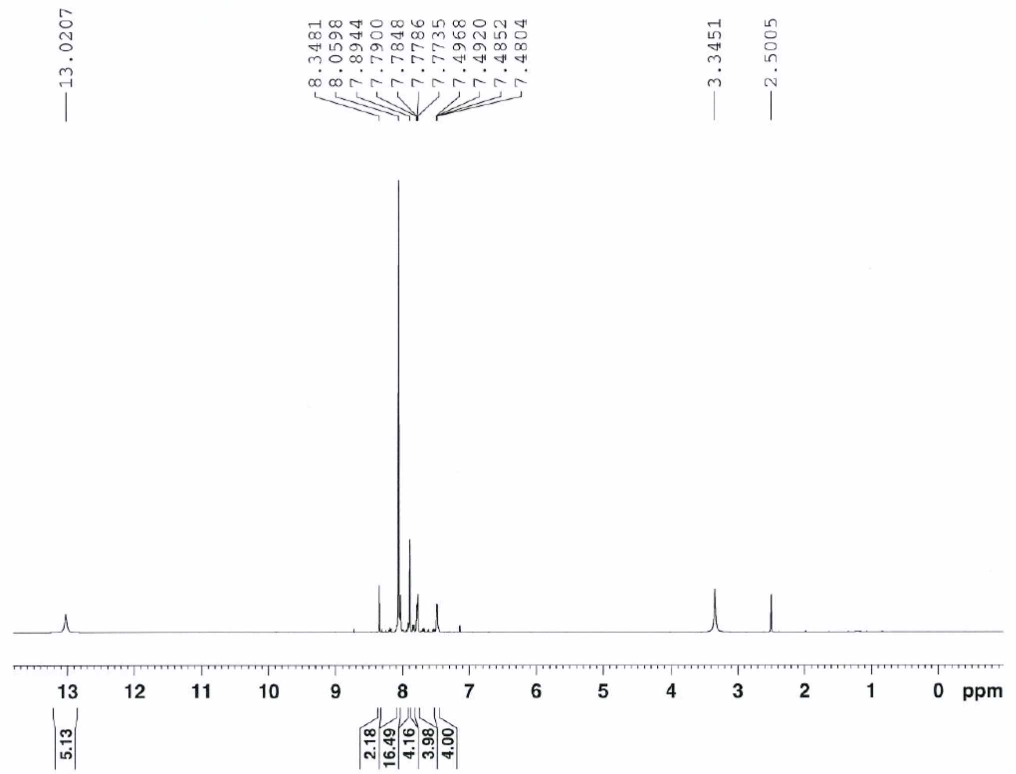

Figure S49: ^{1}H NMR spectrum of 3',3'',5',5''-tetrakis(4-carboxyphenyl)-9,10-diphenylanthracene (600 MHz, DMSO-d_6).

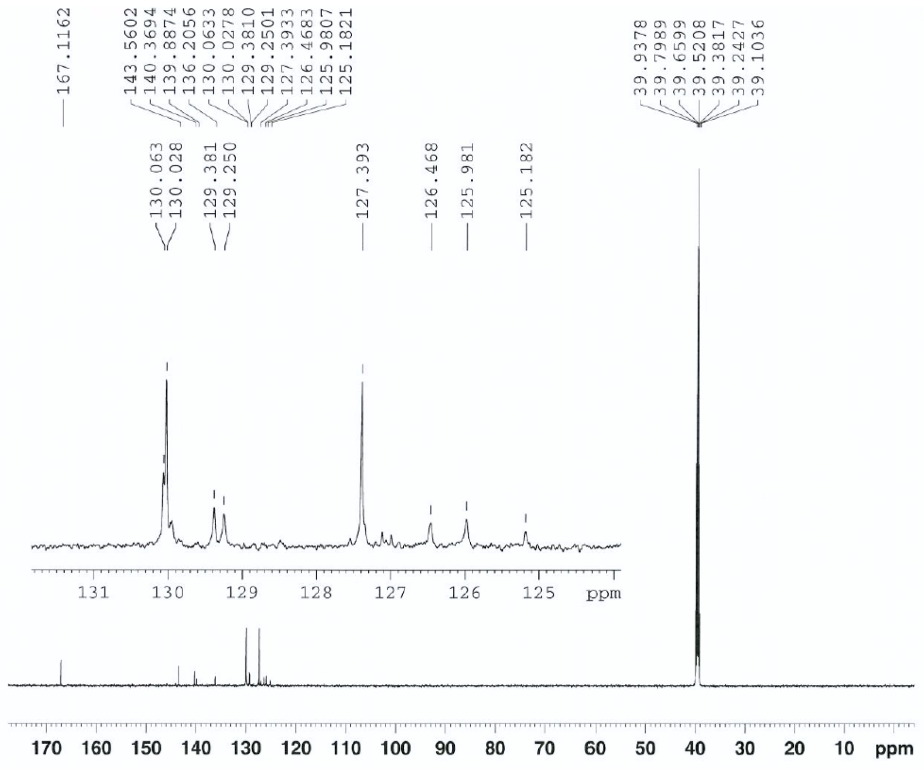

Figure S50: ^{13}C NMR spectrum of 3',3'',5',5''-tetrakis(4-carboxyphenyl)-9,10-diphenylanthracene (150 MHz, DMSO-d_6).

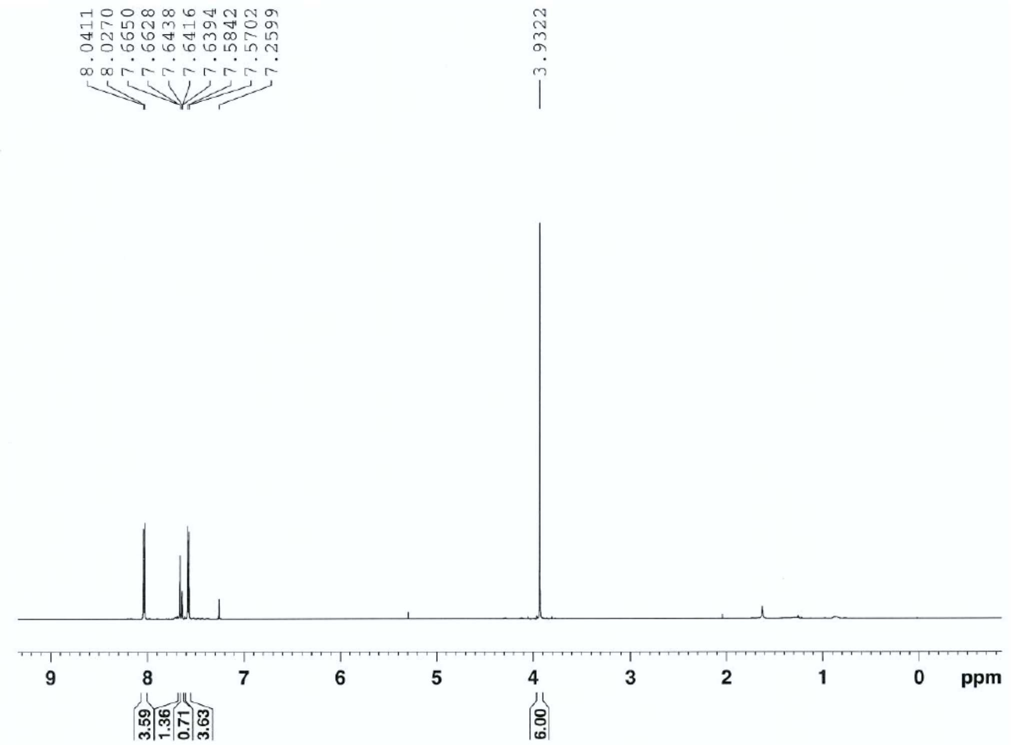

Figure S51: ^{1}H NMR spectrum of 1-bromo-3,5-bis[2-(4-methoxycarbonylphenyl)ethynyl]benzene (600 MHz, $CDCl_3$).

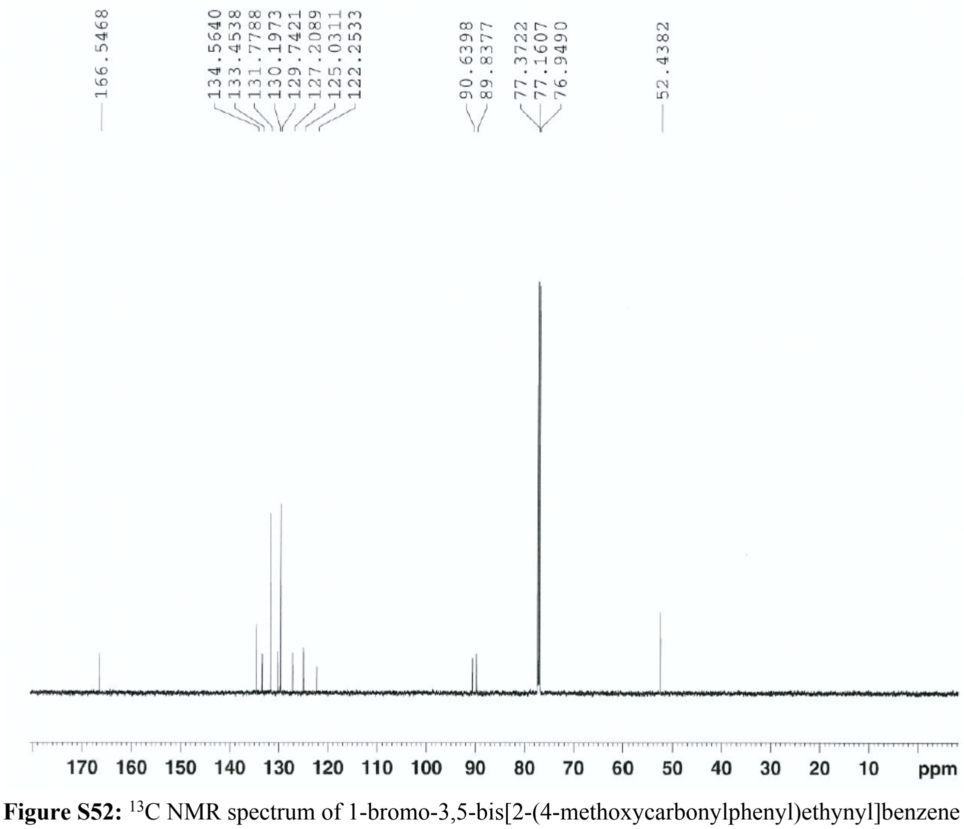

Figure S52: ^{13}C NMR spectrum of 1-bromo-3,5-bis[2-(4-methoxycarbonylphenyl)ethynyl]benzene (150 MHz, $CDCl_3$).

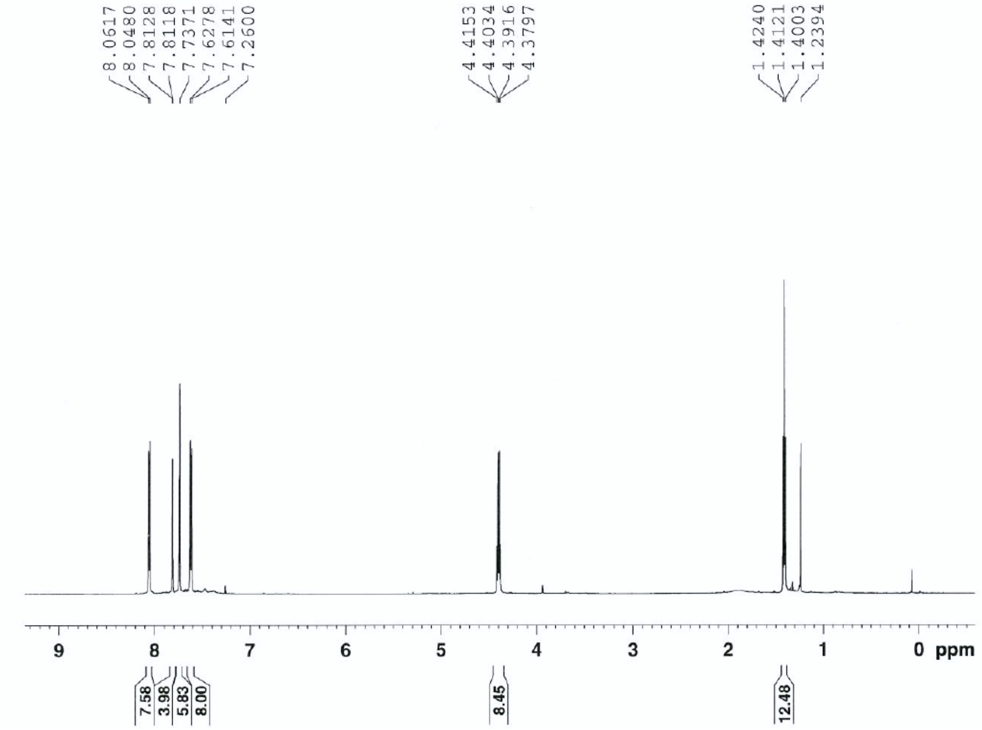

Figure S53: ^{1}H NMR spectrum of 3,3'',5,5''-tetrakis[2-(4-ethoxycarbonylphenyl)ethynyl]-p-terphenyl (600 MHz, $CDCl_3$).

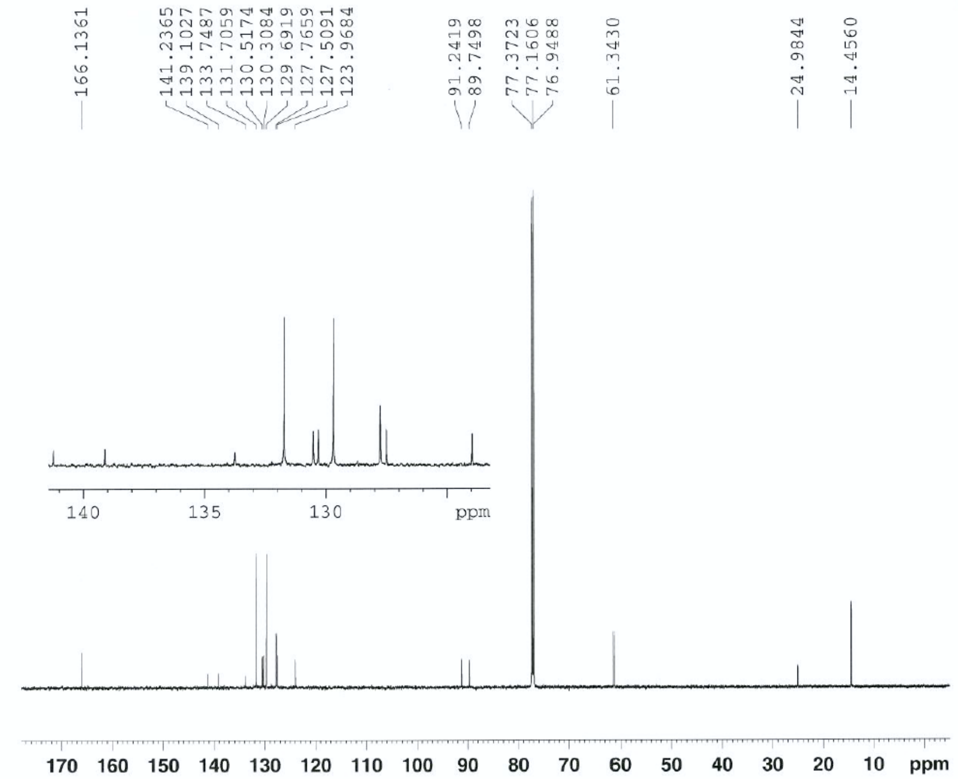

Figure S54: ^{13}C NMR spectrum of 3,3'',5,5''-tetrakis[2-(4-ethoxycarbonylphenyl)ethynyl]-p-terphenyl (150 MHz, $CDCl_3$).

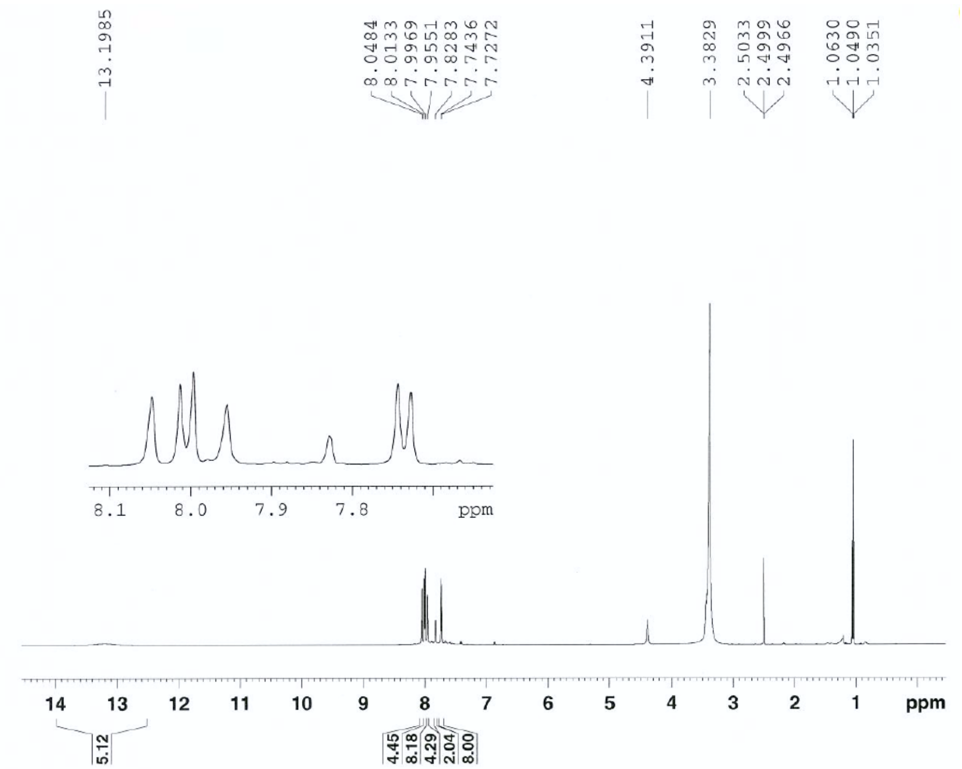

Figure S55: ^{1}H NMR spectrum of 3,3",5,5"-tetrakis[2-(4-carboxylphenyl)ethynyl]-p-terphenyl (500 MHz, DMSO-d_6).

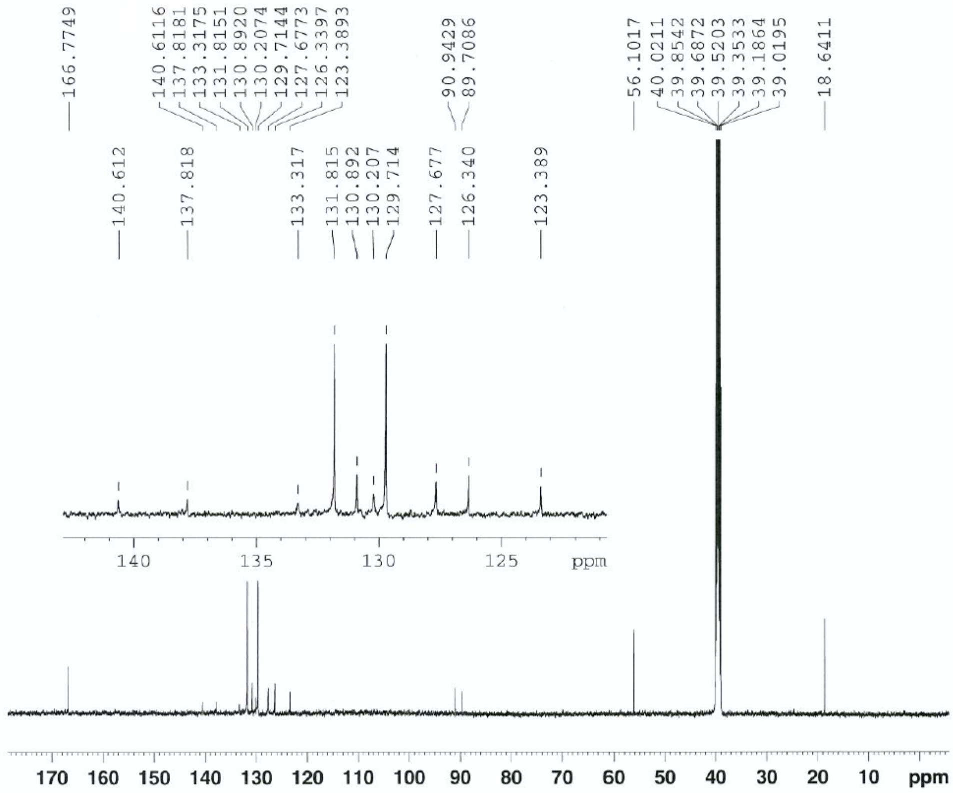

Figure S56: ^{13}C NMR spectrum of 3,3",5,5"-tetrakis[2-(4-carboxyphenyl)ethynyl]-p-terphenyl (125 MHz, DMSO-d_6).

References:

1. V. A. Blatov, A. P. Schevchenko and D. M. Proserpio, *Cryst. Growth. Des.*, 2014, **14**, 3576-3586.
2. A. Bruker, *Bruker AXS, Inc., Madison, WI, USA*, 2009.
3. A. Bruker, *Inc.: Madison, WI*, 1999.
4. G. Sheldrick, University of Göttingen, Germany Program for Empirical Absorption Correction of Area Detector Data, 1996.
5. L. J. Farrugia, *J. Appl. Crystallogr.*, 1999, **32**, 837-838.
6. G. Sheldrick, *Shelx*, 1997.
7. O. V. Dolomanov, L. J. Bourhis, R. J. Gildea, J. A. Howard and H. Puschmann, *J. Appl. Crystallogr.*, 2009, **42**, 339-341.
8. A. Spek, *J. Appl. Crystallogr.*, 2003, **36**, 7-13.
9. Y. He, H. Furukawa, C. Wu, M. O'Keeffe and B. Chen, *CrystEngComm*, 2013, **15**, 9328-9331.
10. D.-X. Xue, A. J. Cairns, Y. Belmabkhout, L. Wojtas, Y. Liu, M. H. Alkordi and M. Eddaoudi, *J. Am. Chem. Soc.*, 2013, **135**, 7660-7667.
11. V. Guillerm, Ł. J. Weseliński, Y. Belmabkhout, A. J. Cairns, V. D'Elia, Ł. Wojtas, K. Adil and M. Eddaoudi, *Nat. Chem.*, 2014, **6**, 673-680.
12. M. Eddaoudi, et.al. *Unpublished Results.*
13. B. Li, H.-M. Wen, H. Wang, H. Wu, M. Tyagi, T. Yildirim, W. Zhou and B. Chen, *J. Am. Chem. Soc.*, 2014, **136**, 6207-6210.
14. Z. Guo, H. Wu, G. Srinivas, Y. Zhou, S. Xiang, Z. Chen, Y. Yang, W. Zhou, M. O'Keeffe and B. Chen, *Angew. Chem. Int. Ed.*, 2011, **50**, 3178-3181.
15. Y. Peng, V. Krungleviciute, I. Eryazici, J. T. Hupp, O. K. Farha and T. Yildirim, *J. Am. Chem. Soc.*, 2013, **135**, 11887-11894.
16. F. Gándara, H. Furukawa, S. Lee and O. M. Yaghi, *J. Am. Chem. Soc.*, 2014, **136**, 5271-5274.
17. J. F. Eubank, H. Mouttaki, A. J. Cairns, Y. Belmabkhout, L. Wojtas, R. Luebke, M. Alkordi and M. Eddaoudi, *J. Am. Chem. Soc.*, 2011, **133**, 14204-14207.
18. E. J. F. Klotz, T. D. W. Claridge and H. L. Anderson, *J. Am. Chem. Soc.*, 2006, **128**, 15374-15375.
19. T. Ishiyama, M. Murata and N. Miyaura, *J. Org. Chem.*, 1995, **60**, 7508-7510.
20. J. F. Eubank, F. Nouar, R. Luebke, A. J. Cairns, L. Wojtas, M. Alkordi, T. Bousquet, M. R. Hight, J. Eckert, J. P. Embs, P. A. Georgiev and M. Eddaoudi, *Angew. Chem. Int. Ed.*, 2012, **51**, 10099-10103.
21. A. M. Fracaroli, H. Furukawa, M. Suzuki, M. Dodd, S. Okajima, F. Gándara, J. A. Reimer and O. M. Yaghi, *J. Am. Chem. Soc.*, 2014, **136**, 8863-8866.
22. S. Grunder, C. Valente, A. C. Whalley, S. Sampath, J. Portmann, Y. Y. Botros and J. F. Stoddart, *Chem. Eur. J.*, 2012, **18**, 15632-15649.
23. B. Gole, A. K. Bar, A. Mallick, R. Banerjee and P. S. Mukherjee, *Chem. Commun.*, 2013, **49**, 7439-7441.
24. K. Cantin, S. Rondeau-Gagne, J. R. Neabo, M. Daigle and J.-F. Morin, *Org. Biomol. Chem.*, 2011, **9**, 4440-4443.
25. Y. Zhang, H.-H. Shi and Y. Cao, *Chin. J. Chem.*, 2006, **24**, 1631-1638.
26. J. M. A. Robinson, B. M. Kariuki, K. D. M. Harris and D. Philp, *J. Chem. Soc., Perkin Trans. 2*, 1998, 2459-2470.
